# Two‐Terminal Molecular Memory through Reversible Switching of Quantum Interference Features in Tunneling Junctions

**DOI:** 10.1002/anie.201807879

**Published:** 2018-10-30

**Authors:** Marco Carlotti, Saurabh Soni, Sumit Kumar, Yong Ai, Eric Sauter, Michael Zharnikov, Ryan C. Chiechi

**Affiliations:** ^1^ Stratingh Institute for Chemistry University of Groningen Nijenborgh 4 9747 AG Groningen The Netherlands; ^2^ Zernike Institute for Advanced Materials Nijenborgh 4 9747 AG Groningen The Netherlands; ^3^ Applied Physical Chemistry Heidelberg University Im Neuenheimer Feld 253 Heidelberg 69120 Germany

**Keywords:** eutectic gallium–indium, memory, molecular electronics, quantum interference, switching

## Abstract

Large‐area molecular tunneling junctions comprising self‐assembled monolayers of redox‐active molecules are described that exhibit two‐terminal bias switching. The as‐prepared monolayers undergo partial charge transfer to the underlying metal substrate (Au, Pt, or Ag), which converts their cores from a quinoid to a hydroquinoid form. The resulting rearomatization converts the bond topology from a cross‐conjugated to a linearly conjugated π system. The cross‐conjugated form correlates to the appearance of an interference feature in the transmission spectrum that vanishes for the linearly conjugated form. Owing to the presence of electron‐withdrawing nitrile groups, the reduction potential and the interference feature lie close to the work function and Fermi level of the metallic substrate. We exploited the relationship between conjugation patterns and quantum interference to create nonvolatile memory in proto‐devices using eutectic Ga–In as the top contact.

Quantum interference (QI) is a collection of phenomena related to fermions, whose wave functions can interfere with themselves; in molecular tunneling junctions, destructive QI can lower the transmission probability between the electrodes, significantly lowering conductance without altering the tunneling distance.^1^ Thus, compounds that produce destructive QI could act as molecular switches, memory devices, or transistors.^2^,^3^ Destructive QI effects have been studied both theoretically and in multiple experimental platforms. In π‐conjugated molecules, they are generally ascribed to cross‐conjugation,^4^, ^5^, ^6^, ^7^ *meta*‐substitution,^8^,^9^ or particular spatial arrangements.^10^,^11^ Of particular interest are systems capable of toggling QI effects through external inputs;^12^, ^13^, ^14^ however, control over QI effects is currently limited to transient, single‐molecule junctions and/or comparisons of different compounds in different environments,^15^, ^16^, ^17^ for example, the ex operando (electro)chemical interconversion between a cross‐conjugated quinone and linearly conjugated hydroquinone.^18^,^19^ Herein we show that self‐assembled monolayers (SAMs) of a cross‐conjugated compound incorporating a tetracyanoquinodimethane (TCNQ) unit, **TCNAQ** (Figure 1), on different metal substrates can be switched between, and addressed in, two conductance states (ON and OFF) in a two‐terminal proto‐device using eutectic Ga–In (EGaIn) top contacts. We ascribe the different conductance states to the modulation of the bond topology of the molecule; **TCNAQ**—just as TCNQ—can readily accept an electron (see Figure S4 in the Supporting Information) and form a stable (di)anion that converts cross‐conjugated pathways to linearly conjugated pathways, thus altering the transmission probability similarly to the interconversion of quinones and hydroquinones (Figure 1).^20^ A low‐lying LUMO brings the reduction potential of **TCNAQ** close to the oxidation potential of Au, Ag, and Pt electrodes, thus eliminating the need for a third electrode or redox agents.

**Figure 1:**
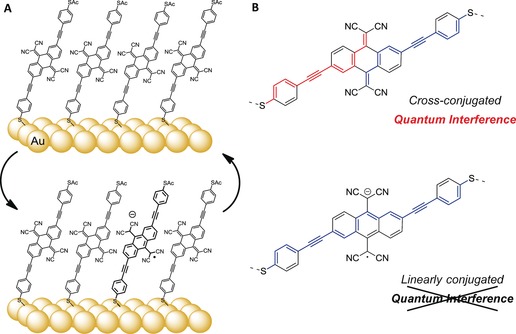
A) A pure monolayer of **TCNAQ** in the cross‐conjugated quinoid form is reversibly switched to a mixed monolayer in which a fraction of the molecules are reduced to a linearly conjugated, hydroquinoid form. B) Difference in conjugation pathways between the cross‐conjugated neutral form (top), which gives rise to destructive QI, and the linearly conjugated reduced form.

We prepared SAMs of **TCNAQ** on Au, Ag, and Pt surfaces from the thioacetate precursor through in situ deprotection.^21^ X‐ray photoelectron spectroscopy (XPS) spectra were consistent with upright‐standing molecules attached to the surface through a single thiolate bond (Figure 2). Synchrotron measurements provide additional evidence that the nitrile groups were oriented predominantly parallel to the substrate (see the Supporting Information for details). The N 1s region of the XPS spectrum of the SAM features an additional peak at lower binding energy (398.5 eV) that is not present in spectra of **TCNAQ** powder, which we ascribe to the spontaneous (partial) reduction of **TCNAQ** by the metal substrate. Similar shifts are common in monolayers of TCNQ that are spontaneously reduced by the underlying metal.^22^ There, TCNQ is directly adsorbed to the metal substrate, whereas in SAMs of **TCNAQ**, the redox‐active core is bound through a phenylacetylene arm that is coupled to the surface through a covalent S−Au bond. Thus, charge transfer (redox) can still occur in a geometry that is compatible with the formation of metal–molecule–metal junctions. In the XPS spectra, about 14 % of **TCNAQ** molecules in the SAM are in a reduced state.

**Figure 2:**
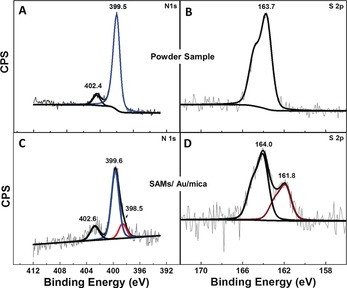
XPS spectra of N 1s and S 2p2/3 core levels for powder samples of **TCNAQ** (top) and SAMs of **TCNAQ** on Au‐on‐mica (bottom). The peak at 398.5 eV, which is present only in the monolayer, indicates the presence of a nonstoichiometric, reduced nitrogen‐containing species.

We investigated the electrical properties of **TCNAQ** in Au‐on‐mica/SAM//EGaIn junctions (where “/” and “//” denote covalent and Van der Waals interaction, respectively). EGaIn is a liquid metal that can be used to form stable, conformal, nondamaging contacts with SAMs with a diameter of about 20 μm.^23^, ^24^, ^25^ This methodology enables the formation of junctions in multiple areas of a substrate rapidly and reproducibly, thus allowing the collection of statistically significant data and spectroscopic investigation of the SAM after *J*/*V* cycling.^26^ As controls, we measured junctions comprising hexadecanethiol (**C16SH**) and analogues of **TCNAQ** bearing an anthraquinone core (**AQ**) or a linearly conjugated, non‐redox‐active anthracene core (**AC**; see Figure S1).

Figure 3 shows forward and reverse *J*/*V* traces for junctions comprising **TCNAQ** (A), **AC** (B), **C16SH** (C), and **AQ** (D). While the *J*/*V* traces of the latter three junctions overlap perfectly, **TCNAQ** exhibits a hysteresis loop at negative bias; that is, after being biased at positive voltages, the conductance at negative bias decreases (OFF) and then recovers its initial conductance (ON) after reaching −1.00 V. A maximum ratio of *J* between forward and reverse scans of 2.6 occurred at −0.65 V. The hysteresis and magnitude of switching was reproducible across junctions comprising **TCNAQ** on Au‐on‐mica and template‐stripped^27^ (TS) Au^TS^ and Ag^TS^ (Figure 4). The effect was present but diminished on Pt^TS^ (see Figures S27 and S28). No hysteresis or switching was present on any substrate for **AC**, **AQ**, or **C16SH**.

**Figure 3:**
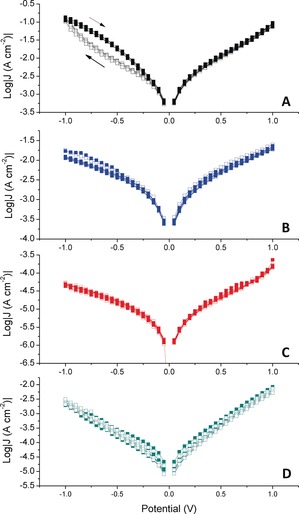
Examples of log |*J*| versus *V* of junctions comprising SAMs of **TCNAQ** on Au^TS^ (A, black), **AC** (B, blue), **C16SH** (C, red), and **AQ** (on Au‐on‐mica, D, cyan). Solid dots represent data acquired during five forward scans ranging from −1.00 to 1.00 V (in 0.05 V steps, acquired every 0.1 s), while open dots represent data acquired during five subsequent reverse scans from 1.00 to −1.00 V.

**Figure 4:**
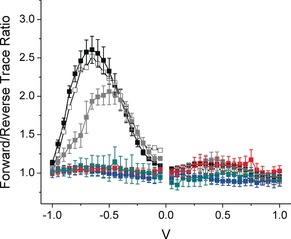
Plot of trace over retrace ratio for junctions comprising: SAMs of **TCNAQ** on Au^TS^ (black), Au‐on‐mica (hollow), and Ag^TS^ (gray), **C16SH** on Ag^TS^ (red), **AC** on Au^TS^ (blue), **AQ** on Au‐on‐mica (cyan). Error bars represent confidence intervals with *α*=0.05 (for **TCNAQ** on Au‐on‐mica they were omitted for clarity).

Given sufficient trace/retrace stability, hysteresis is a form of two‐terminal bias switching,^28^, ^29^, ^30^, ^31^, ^32^, ^33^, ^34^, ^35^, ^36^, ^37^, ^38^, ^39^, ^40^, ^41^, ^42^, ^43^ but to translate it into a memory effect, the state of a static, two‐terminal junction must be switched reversibly between at least two conductance states in operando. To characterize the memory effects of metal/SAM//EGaIn junctions, we performed write operations (W) by applying a −1.50 V bias, which puts the junction in the high‐conductance ON state, and erase operations (E) by applying 1.00 V, which puts it in the low‐conductance OFF state. We read the state at −0.50 V, measuring current densities *J* of 0.10–0.01 A cm^−2^. Figure 5 A compares the resulting ON/OFF ratios for junctions comprising SAMs of **TCNAQ**, **AC**, **AQ**, and **C16SH** on Au over four switching cycles. As expected, the ON/OFF ratio for the controls (**AC**, **AQ**, and **C16SH**) was 1, indicating no effect. However, **TCNAQ** exhibited ratios as high as 6±2. The memory effect was identical for Au‐on‐mica and Au^TS^, but the peak switching ratio decreased and shifted to less negative bias on Ag^TS^ (Figure 5 A). On Pt^TS^, the peak decreased and shifted further, and the hysteresis became noticeable only over a larger bias window (see the Supporting Information for details). This trend is consistent with the proposed mechanism because the magnitude of the suppression of conductance scales with the proximity of the destructive QI feature to the Fermi level.^44^

**Figure 5:**
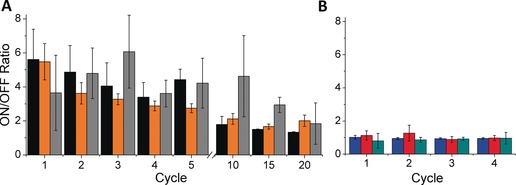
ON/OFF ratio per *n*th write–erase cycle, calculated as the ratio of the current after and before the *n*th write operation for: A) SAMs of **TCNAQ** on Au^TS^ (black), Au‐on‐mica (orange), and Ag^TS^ (gray), and B) SAMs of C16SH on Ag^TS^ (red), AC on Au^TS^ (blue), and AQ on Au‐on‐mica (cyan). The error is calculated as the confidence interval with *α*=0.05.

As shown in Figure 5, the ON/OFF ratio of **TCNAQ** slowly decreased, approaching 2 after about 10 cycles; however, even after 30 cycles, applying a −2.00 V bias restored the initial ON/OFF ratio (see Figure S34). The conductance of **TCNAQ** decreased with each W–E cycle (see Table S3 in the Supporting Information), which is typical for repeated switching cycles in molecular tunneling junctions.^15^ For junctions comprising **TCNAQ**, such damping could be the result of irreversible reactions, structural modification of the SAMs, or a change in the EGaIn contact. However, because the conductance remained constant during *J*/*V* sweeps (Figure 3, as opposed to R–E–W cycles, i.e., Figure 5), the underlying phenomenon is specific to large, rapid changes in bias.

In its pristine state, a destructive QI feature is clearly present in the transmission spectrum near *E*_f_ (see Figure S44 A).^45^ In single‐molecule STM break‐junctions comprising **TCNAQ**, this QI feature manifests as a low conductance, comparable to that of **AQ**, which is known to exhibit strong destructive QI effects in both single‐molecule and SAM‐based junctions (see Figure S41).^6^,^46^ However, ensemble junctions comprising SAMs of **TCNAQ** exhibit a relatively high conductance, comparable to that of the linearly conjugated analogue **AC** (see Figure S10), which does not exhibit any QI features.

We ascribe the difference between **TCNAQ** in SAMs and in single‐molecule junctions to the presence of reduced **TCNAQ** in the SAM (Figure 2 C).^47^ Tunneling charge transport through SAMs is sensitive to the entire supramolecular structure of the monolayer, which comprises molecules in different conformations and, in the case of **TCNAQ**, redox states.^11^,^48^ Figure 1 B shows the bond topology of **TCNAQ** in the pristine and reduced states. The addition of one or two electrons converts the cross‐conjugated quinoid core into a linearly conjugated, fully aromatic hydroquinoid. (The driving force of rearomatization is the reason that TCNQ is an exceptional electron acceptor.) Treating each molecule in a SAM as a resistor in parallel, it follows from the Kirchoff rules that a small fraction of reduced **TCNAQ** molecules can dominate charge transport through the SAM owing to the exponential difference in the conductance of **TCNAQ** in the cross‐ and linearly conjugated (quinoid/hydroquinoid) forms.^49^ Specifically, if two pathways in a SAM differ in conductance by two orders of magnitude (similar to **AQ** and **AC** at 0.50 V), the presence of only 1–2 % of the more conductive pathways is sufficient to dominate the conductivity of the SAM.^50^ Thus, the hysteresis and switching phenomenon are most likely caused by a shift in the equilibrium between the low‐ and high‐conductance states of **TCNAQ**; applying a bias to the junction affects the fraction of molecules in the junction that exhibit destructive QI.

If the proposed mechanism is correct, the ON state is metastable and should slowly relax to lower conductance, since the thermodynamic minimum is the neutral, low‐conductance state. Indeed, the ON state current decreases in time with multiple read cycles, whereas the OFF state only shows small, stochastic fluctuations, which are discussed further in the Supporting Information. The initial ON/OFF ratio is restored after a new W cycle; that is, the application of a pulse at negative bias restores the SAM to the initial state, in which a greater fraction of **TCNAQ** is in the reduced state. We ascribe this observation to the slow kinetics of the relaxation (reoxidation) process. When a charge is placed in a SAM by the reduction of a molecule, the local environment in the SAM reorganizes to minimize the free energy of the system, which is a much slower process than the initial electron transfer. Within 5 min, without any applied bias, the ON/OFF ratio decreases to 70 % of its initial value; after 20 min it has decreased to 60 %.

Metal/**TCNAQ**//EGaIn junctions are a form of nonvolatile memory; their state is retained when the power (bias) is removed. It is difficult to contextualize **TCNAQ** further. There are examples of memory effects in molecular tunneling junctions, each demonstrating a salient feature: Some exhibit high switching ratios as single molecules, but not in (proto‐)device platforms;^37^,^42^ some require prohibitively complex fabrication;^35^ some only switch at low temperature;^28^ some are resistant to fatigue when switched with light, but not with bias.^51^ In simple, two‐terminal proto‐devices, **TCNAQ** exhibits reasonably high ON/OFF ratios that are stable for tens of minutes and that can be refreshed or rewritten over at least dozens of cycles. The switching mechanism is phenomenologically unique, exploiting the coincidental alignment of a destructive QI feature and facile reduction with the Fermi level and work function of Au to enable the shift of a dynamic equilibrium of molecules in high‐conductance states lacking QI features and low‐conductance states with strong QI features near the Fermi level. The switching effect is molecular in nature, and further investigation and optimization could feasibly exploit this type of QI‐based switching to achieve switching ratios of orders of magnitude.

## Conflict of interest

The authors declare no conflict of interest.

## Supporting information

As a service to our authors and readers, this journal provides supporting information supplied by the authors. Such materials are peer reviewed and may be re‐organized for online delivery, but are not copy‐edited or typeset. Technical support issues arising from supporting information (other than missing files) should be addressed to the authors.

Supplementary

## References

[1] M. H. Garner , H. Li , Y. Chen , T. A. Su , Z. Shangguan , D. W. Paley , T. Liu , F. Ng , H. Li , S. Xiao , C. Nuckolls , L. Venkataraman , G. C. Solomon , Nature 2018, 406, 1. 10.1038/s41586-018-0197-9 29875407

[2] F. Schwarz , M. Koch , G. Kastlunger , H. Berke , R. Stadler , K. Venkatesan , E. Lörtscher , Angew. Chem. Int. Ed. 2016, 55, 11781–11786; 10.1002/anie.201605559 27553767

[3] S. Chen , W. Zhou , Q. Zhang , Y. Kwok , G. Chen , M. A. Ratner , J. Phys. Chem. Lett. 2017, 8, 5166–5170. 28974091 10.1021/acs.jpclett.7b02214

[4] Y. Tsuji , R. Hoffmann , R. Movassagh , S. Datta , J. Chem. Phys. 2014, 141, 224311. 25494753 10.1063/1.4903043

[5] T. Markussen , R. Stadler , K. S. Thygesen , Nano Lett. 2010, 10, 4260–4265. 20879779 10.1021/nl101688a

[6] D. Fracasso , H. Valkenier , J. C. Hummelen , G. C. Solomon , R. C. Chiechi , J. Am. Chem. Soc. 2011, 133, 9556–9563. 21561156 10.1021/ja202471m

[7] K. G. L. Pedersen , A. Borges , P. Hedegård , G. C. Solomon , M. Strange , J. Phys. Chem. C 2015, 119, 26919–26924.

[8] D. Z. Manrique , C. Huang , M. Baghernejad , X. Zhao , O. a. Al-Owaedi , H. Sadeghi , V. Kaliginedi , W. Hong , M. Gulcur , T. Wandlowski , M. R. Bryce , C. J. Lambert , Nat. Commun. 2015, 6, 6389. 25731608 10.1038/ncomms7389

[9] M. Gantenbein , L. Wang , A. A. Al-jobory , A. K. Ismael , C. J. Lambert , M. R. Bryce , Sci. Rep. 2017, 7, 1794. 28496118 10.1038/s41598-017-01903-0 PMC5431762

[10] G. C. Solomon , C. Herrmann , J. Vura-Weis , M. R. Wasielewski , M. A. Ratner , J. Am. Chem. Soc. 2010, 132, 7887–7889. 20486660 10.1021/ja102434m

[11] M. Carlotti , A. Kovalchuk , T. Wächter , X. Qiu , M. Zharnikov , R. C. Chiechi , Nat. Commun. 2016, 7, 13904. 27996036 10.1038/ncomms13904 PMC5187444

[12] H. Jeong , D. Kim , D. Xiang , T. Lee , ACS Nano 2017, 11, 6511–6548. 28578582 10.1021/acsnano.7b02967

[13] Y. Selzer , L. Cai , M. A. Cabassi , Y. Yao , J. M. Tour , T. S. Mayer , D. L. Allara , Nano Lett. 2005, 5, 61–65. 15792413 10.1021/nl048372j

[14] C. Jia , et al., Science 2016, 352, 1443–1445. 27313042 10.1126/science.aaf6298

[15] Z. Liu , S. Ren , X. Guo , Top. Curr. Chem. 2017, 375, 56. 10.1007/s41061-017-0144-5 28493206

[16] G. Yang , et al., Chem. Sci. 2017, 8, 7505–7509. 29163904 10.1039/c7sc01014a PMC5676185

[17] J. Liu , X. Zhao , Q. Al-Galiby , X. Huang , J. Zheng , R. Li , C. Huang , Y. Yang , J. Shi , D. Z. Manrique , C. J. Lambert , M. R. Bryce , W. Hong , Angew. Chem. Int. Ed. 2017, 56, 13061–13065; 10.1002/anie.201707710 28771925

[18] M. Koole , J. M. Thijssen , H. Valkenier , J. C. Hummelen , H. S. J. van der Zant , Nano Lett. 2015, 15, 5569–5573. 26182342 10.1021/acs.nanolett.5b02188

[19] M. Baghernejad , X. Zhao , K. Baruël Ørnsø , M. Füeg , P. Moreno-García , A. V. Rudnev , V. Kaliginedi , S. Vesztergom , C. Huang , W. Hong , P. Broekmann , T. Wandlowski , K. S. Thygesen , M. R. Bryce , J. Am. Chem. Soc. 2014, 136, 17922–17925. 25494539 10.1021/ja510335z

[20] T. Markussen , J. Schiotz , K. S. Thygesen , J. Chem. Phys. 2010, 132, 224104. 20550387 10.1063/1.3451265

[21] H. Valkenier , E. H. Huisman , P. A. van Hal , D. M. de Leeuw , R. C. Chiechi , J. C. Hummelen , J. Am. Chem. Soc. 2011, 133, 4930–4939. 21384876 10.1021/ja110358t

[22] T.-C. Tseng , et al., Nat. Chem. 2010, 2, 374. 20414237 10.1038/nchem.591

[23] R. C. Chiechi , E. A. Weiss , M. D. Dickey , G. M. Whitesides , Angew. Chem. Int. Ed. 2008, 47, 142–144; 10.1002/anie.200703642 18038438

[24] M. D. Dickey , R. C. Chiechi , R. J. Larsen , E. A. Weiss , D. A. Weitz , G. M. Whitesides , Adv. Funct. Mater. 2008, 18, 1097–1104.

[25] L. Cademartiri , M. M. Thuo , C. A. Nijhuis , W. F. Reus , S. Tricard , J. R. Barber , R. N. S. Sodhi , P. Brodersen , C. Kim , R. C. Chiechi , G. M. Whitesides , J. Phys. Chem. C 2012, 116, 10848–10860.

[26] S. Kumar , J. T. van Herpt , R. Y. N. Gengler , B. L. Feringa , P. Rudolf , R. C. Chiechi , J. Am. Chem. Soc. 2016, 138, 12519–12526. 27602432 10.1021/jacs.6b06806 PMC5053170

[27] E. A. Weiss , G. K. Kaufman , J. K. Kriebel , Z. Li , R. Schalek , G. M. Whitesides , Langmuir 2007, 23, 9686–9694. 17696377 10.1021/la701919r

[28] J. Chen , M. A. Reed , A. M. Rawlett , J. M. Tour , Science 1999, 286, 1550. 10567259 10.1126/science.286.5444.1550

[29] J. Chen , M. Reed , Chem. Phys. 2002, 281, 127–145.

[30] A. S. Blum , J. G. Kushmerick , D. P. Long , C. H. Patterson , J. C. Yang , J. C. Henderson , Y. Yao , J. M. Tour , R. Shashidhar , B. R. Ratna , Nat. Mater. 2005, 4, 167. 15654344 10.1038/nmat1309

[31] N. Gergel-Hackett , N. Majumdar , Z. Martin , N. Swami , L. R. Harriott , J. C. Bean , G. Pattanaik , G. Zangari , Y. Zhu , I. Pu , Y. Yao , J. M. Tour , J. Vac. Sci. Technol. A 2006, 24, 1243–1248.

[32] Y. Chen , G.-Y. Jung , D. A. A. Ohlberg , X. Li , D. R. Stewart , J. O. Jeppesen , K. A. Nielsen , J. F. Stoddart , R. S. Williams , Nanotechnology 2003, 14, 462.

[33] C. P. Collier , G. Mattersteig , E. W. Wong , Y. Luo , K. Beverly , J. Sampaio , F. M. Raymo , J. F. Stoddart , J. R. A. Heath , Science 2000, 289, 1172. 10947980 10.1126/science.289.5482.1172

[34] E. W. Wong , C. P. Collier , M. Běhloradský , F. M. Raymo , J. F. Stoddart , J. R. Heath , J. Am. Chem. Soc. 2000, 122, 5831–5840.

[35] J. E. Green , J. Wook Choi , A. Boukai , Y. Bunimovich , E. Johnston-Halperin , E. DeIonno , Y. Luo , B. A. Sheriff , K. Xu , Y. Shik Shin , H.-R. Tseng , J. F. Stoddart , J. R. A. Heath , Nature 2007, 445, 414. 17251976 10.1038/nature05462

[36] K. Seo , A. V. Konchenko , J. Lee , G. S. Bang , H. Lee , J. Am. Chem. Soc. 2008, 130, 2553–2559. 18251540 10.1021/ja077089u

[37] J. Lee , H. Chang , S. Kim , G. Bang , H. Lee , Angew. Chem. Int. Ed. 2009, 48, 8501–8504; 10.1002/anie.200902990 19795425

[38] B. Pradhan , S. Das , Chem. Mater. 2008, 20, 1209–1211.

[39] K. Seo , A. V. Konchenko , J. Lee , G. S. Bang , H. Lee , J. Mater. Chem. 2009, 19, 7617–7624.

[40] C. Li , W. Fan , B. Lei , D. Zhang , S. Han , T. Tang , X. Liu , Z. Liu , S. Asano , M. Meyyappan , J. Han , C. Zhou , Appl. Phys. Lett. 2004, 84, 1949–1951.

[41] C. Li , J. Ly , B. Lei , W. Fan , D. Zhang , J. Han , M. Meyyappan , M. Thompson , C. Zhou , J. Phys. Chem. B 2004, 108, 9646–9649.

[42] S. Seo , J. Lee , S.-Y. Choi , H. Lee , J. Mater. Chem. 2012, 22, 1868–1875.

[43] M. Min , S. Seo , S. M. Lee , H. Lee , Adv. Mater. 2013, 25, 7045–7050. 24133048 10.1002/adma.201303335

[44] Y. Zhang , G. Ye , S. Soni , X. Qiu , T. L. Krijger , H. T. Jonkman , M. Carlotti , E. Sauter , M. Zharnikov , R. C. Chiechi , Chem. Sci. 2018, 9, 4414–4423. 29896382 10.1039/c8sc00165k PMC5961448

[45] H. Valkenier , C. M. Guedon , T. Markussen , K. S. Thygesen , S. J. van der Molen , J. C. Hummelen , Phys. Chem. Chem. Phys. 2014, 16, 653–662. 24270575 10.1039/c3cp53866d

[46] V. Kaliginedi , P. Moreno-García , H. Valkenier , W. Hong , V. M. García-Suárez , P. Buiter , J. L. H. Otten , J. C. Hummelen , C. J. Lambert , T. Wandlowski , J. Am. Chem. Soc. 2012, 134, 5262–5275. 22352944 10.1021/ja211555x

[47] R. García , M. Ángeles Herranz , E. Leary , M. T. Gonz‘alez , G. R. Bollinger , M. Bu“rkle , L. A. Zotti , Y. Asai , F. Pauly , J. C. Cuevas , N. Agraít , N. Mart’ın , Beilstein J. Org. Chem. 2015, 11, 1068–1078. 26199662 10.3762/bjoc.11.120 PMC4505095

[48] A. Kovalchuk , D. A. Egger , T. Abu-Husein , E. Zojer , A. Terfort , R. C. Chiechi , RSC Adv. 2016, 6, 69479–69483.

[49] P. Pourhossein , R. K. Vijayaraghavan , S. C. J. Meskers , R. C. Chiechi , Nat. Commun. 2016, 7, 11749. 27272394 10.1038/ncomms11749 PMC4899853

[50] E. A. Weiss , R. C. Chiechi , G. K. Kaufman , J. K. Kriebel , Z. Li , M. Duati , M. A. Rampi , G. M. Whitesides , J. Am. Chem. Soc. 2007, 129, 4336–4349. 17358061 10.1021/ja0677261

[51] S. Seo , M. Min , S. M. Lee , H. Lee , Nat. Commun. 2013, 4, 1920. 23715279 10.1038/ncomms2937

