# Supplementary material for: Two‐Terminal Molecular Memory through Reversible Switching of Quantum Interference Features in Tunneling Junctions
Source: Angew Chem Int Ed Engl. 2018 Oct 30;57(48):15681–5. doi: 10.1002/anie.201807879 (PMC6283355; doi:10.1002/anie.201807879)
Supplement: Supplementary file 1 — Supplementary [file ANIE-57-15681-s001.pdf]

Angewandte Chemie
Eine Zeitschrift der Gesellschaft Deutscher Chemiker

Supporting Information

Two-Terminal Molecular Memory through Reversible Switching of Quantum Interference Features in Tunneling Junctions

*Marco Carlotti, Saurabh Soni, Sumit Kumar, Yong Ai, Eric Sauter, Michael Zharnikov, and Ryan C. Chiechi**

anie_201807879_sm_miscellaneous_information.pdf

Contents

1 Materials **S-3**

1.1 Synthesis of **TCNAQ** S-5

2 Preparation the SAMs **S-10**

2.1 X-ray Photoelectron Spectroscopy S-10

2.2 Experiments at the synchrotron S-15

3 Acquisition and processing of data **S-19**

3.1 $J-V$ curves S-19

3.2 Write-Read-Erase-Read (WRER) cycles S-38

4 Stability of TCNAQ junctions during WRER operations **S-47**

5 Relations between the fraction of reduced molecules and the increase in conductance **S-50**

6 Stability of TCNAQ SAMs in different environments **S-52**

7 Properties of junctions comprising SAMs of TCNAQ on Pt^{TS} **S-55**

8 Single molecule conductance measurement **S-57**

9 Calculations **S-60**

9.0.1 Geometry Optimization S-60

9.0.2 Single Point Energy Calculations S-61

9.1 Transport Properties S-61

9.1.1 XYZ Coordinates S-66

References **S-75**

1 Materials

All reagents were purchased from Sigma-Aldrich, Acros, or TCI Europe and used as received unless otheriwse stated. Triethylamine and $CHCl_3$ were distilled over CaH and P_2O_5 respectively, and used within 10 days. Acetonitrile, dichloromethane (DCM), and tetrahydrofuran (THF) were obtained anhydrous from a house system. Hexadecanethiol (**C16SH**) was purified by coloumn cromatography (silica, hexane, R_f= 0.85). NMR spectra were recorded on a Varian AMX400 (400MHz) and referenced to the solvent peak ($CDCl_3$: H, 7.26 ppm; C, 77 ppm) relative to tetramethylsilane. (4-iodo)phenyl-*tert*-butyl-thioether (**1**) was prepared elsewhere.[S1] S,S'-(((9,10-bis(dicyanomethylene)-9,10-dihydroanthracene-2,6-diyl)bis(ethyne-2,1-diyl))bis(4,1-phenylene)) diethanethioate (**TCNAQ**) was prepared according to the scheme showed in figure S2. S,S'-((anthracene-2,6-diylbis(ethyne-2,1-diyl))bis(4,1-phenylene)) diethanethioate (**AC**) and S,S'-(((9,10-anthraquinone-2,6-diyl)bis(ethyne-2,1-diyl))bis(4,1-phenylene)) diethanethioate (**AQ**) were prepared elsewere.[S1] Their structures are reported in Figure S1. Template stripped metal substrates were prepared by depositing a 100 nm-thick layer of metal on a Si wafer in a metal evaporator (Au and Ag) or by e-beam (Pt). 1x1x0.3 cm glass slides were glued to the deposited metal using an UV-curable optical adhesive (Norland series 60). The samples were cleaved from the wafer with the help of a razor and immediately used. Au-on-mica (1x1 cm, 200 nm thick Au) was obtained from Phasis and kept in the original packing in a glovebox until use.

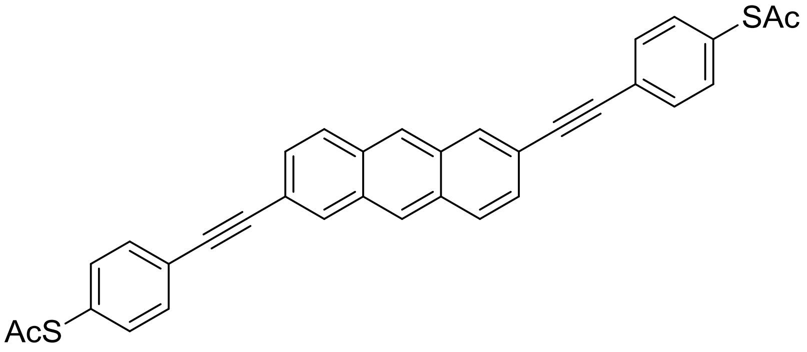

AC

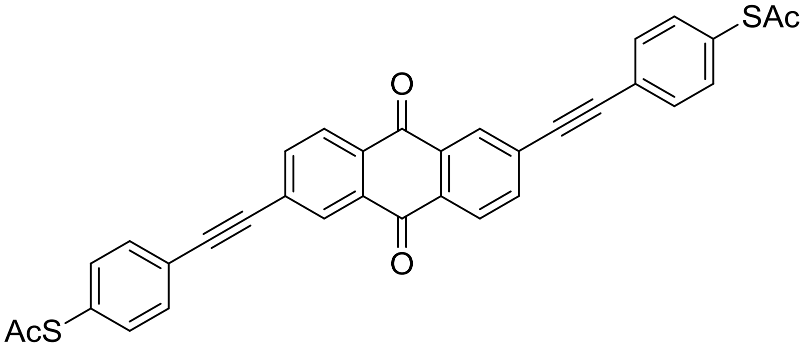

AQ

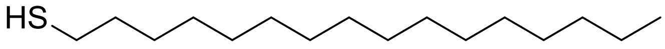

C16SH

Figure S1: Structures of **AC**, **AQ**, and **C16SH**

1.1 Synthesis of TCNAQ

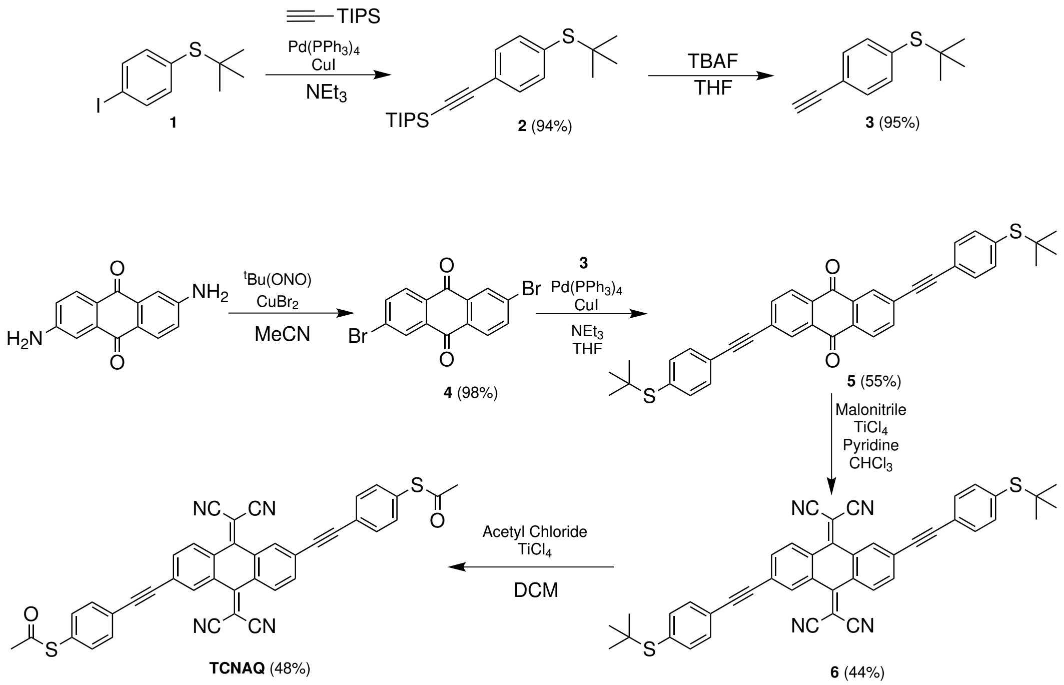

Figure S2: Synthetic scheme for **TCNAQ**.

((4-(*tert*-butylthio)phenyl)ethynyl)triisopropylsilane, 2

In an oven dry Schlenk under N_2, 1.56 g of **1** (6.36 mmol), 1.65 mL of (triisopropylsilyl)acetylene (7.34 mmol), 210 mg of $Pd(PPh_3)_4$, and 76 mg of CuI were dissolved in 15 mL of NEt_3. The solution was heated to 65 °C and left under stirring 16 hours. The solution was then poured in 100 mL of water and 40 mL of hexane kept under vigorous stirring. 20 mL of 6 M HCl were added and the two phases eparated. The water phase was extracted with once with hexane and the united organic phases three times with water. The hexane solution was reduced under vacuum and filtered over a plug of silica. The solvent was finally removed under vacuum and the product was obtained as a pale yellow oil (1.99g, 94% yield). ^{1}H NMR ($CDCl_3$, 400MHz): 7.48-7.42 (m, 4H), 1.28 (s, 9H), 1.14 (s, 21H).

***tert*-butyl(4-ethynylphenyl)sulfide, 3**

In a 250 mL flask, 3 g of **2** were dissolved in 150 mL of THF and the solution placed in an ice bath. 17.3 mL of a tetrabutylammonium fluoride solution 1 M in THF (containing 5%

wt. water) were added dropwise over the period of an hour. After 30 additional minutes, the ice bath was removed and the solution left under stirring for 4 hours. The solution was then dried over Na_2SO_4 and adsorbed on silica. The product was purified trough a small silica coloumn using hexane as eluent. After removing the solvent under vacuum, the product was recovered as a colourless oil (1.57 g, 95% yield). ^{1}H NMR ($CDCl_3$, 400MHz): 7.49-7.42 (m, 4H), 3.12 (s, 1H), 1.25 (s, 9H).

2,6-dibromoanthracene-9,10-dione, 4

In a oven dry 250 mL flask under N_2, 6.87 g of 2,6-diaminoanthraquinone (28.6 mmol) and 15.63 g of $CuBr_2$ (71.5 mmol) were dissolved in 125 mL of dry acetonitrile. 8 mL of *tert*-butyl nitrite were addedo dropwise over a period of 30 minutes. the system was heated up to 70 °C for 4 hours. Formation of a brown precipitate was evident. The reaction mixture was allowed to room temperature and poured in 1 L of stirring HCl 6 M. The precipitate was then filtered, washed with water until neutrality, and with 100 mL of acetonitrile. The precipitate was then recrystallized from dioxane to yield a dark yello solid (7.87 g, 98% yield). ^{1}H NMR ($CDCl_3$, 400MHz): 8.44 (d, J= 2.0 Hz, 2H), 8.17 (d, J= 8.3 Hz, 2H), 7.94 (dd, J= 2.0, 8.3 Hz, 2H). ^{13}C NMR ($CDCl_3$, 400MHz): 179.66, 135.89, 132.97, 130.41, 128.83, 128.68, 127.60.

2,6-bis((4-(*tert*-butylthio)phenyl)ethynyl)anthracene-9,10-dione, 5

In an oven dry Schlenk under N_2, 0.77 g of **4** (2.1 mmol), 1.20 g of **3** (6.31 mmol), 121 mg of $Pd(PPh_3)_4$, and 40 mg of CuI, and 10 mL of distilled NEt_3 were dissolved in 15 mL of dry THF. The solution was heated to 85 °C and left under stirring for 24 hours. the solution was allowed to cool down and poured in 100 mL of water. 50 mL of DCM were added and the water phase acidified with 30 mL of HCl 6 M. The water phase was extracted with DCM and the combined organic phases were filtered, extracted three times with water, and passed trough a plug of silica. The solvent was removed under vacuum and the obtained solid recrystallized from toluene to yield yellow crystals (594 mg, 55% yield). ^{1}H NMR ($CDCl_3$, 400MHz): 8.44 (d, J= 1.6 Hz, 2H), 8.32 (d, J= 8.1 Hz, 2H), 7.91 (dd, J= 1.6 8.1 Hz, 2H), 7.57-7.52 (m, 8H), 1.32 (s, 18H). ^{13}C NMR ($CDCl_3$, 400MHz): 181.92, 137.28, 136.56,

134.58, 133.50, 132.27, 131.74, 130.33, 129.61, 127.53, 122.48, 93.95, 89.35, 46.72, 31.02.

2,2'-(2,6-bis((4-(tert-butylthio)phenyl)ethynyl)anthracene-9,10-diylidene)- dimalononitrile, 6

In a dry 100 mL flask equipped with a cooler under N_2, 133 mg of **5** (0.23 mmol) and 65 mg of malononitrile (0.93 mmol) were dissolved in 50 mL of dry $CHCl_3$. The solution was brought to reflux and 0.10 mL of $TiCl_4$ (0.93 mmol) and 0.15 mL of pyridine (1.89 mmol) were added. The system was left at reflux for 18 hours. The reaction mixture was quenched with 50 mL of water. The water phase was extracted with chloroform thrice and the combined organic phases were extracted twice with water. They were then dried over sodium sulfate and the solvent removed in vacuum. The residue was adsorbed on silica and purified via coloumn chromatography (SiO_2, hexane:ethyl acetate 6:1, R_f= 0.35). A red solid is obtained (73 mg, 44% yield). ^{1}H NMR ($CDCl_3$, 400MHz): 8.32 (d, J= 1.5 Hz, 2H), 8.22 (d, J= 8.2 Hz, 2H), 7.82 (dd, J= 1.5, 8.2 Hz, 2H), 7.56-7.51 (m, 8H), 1.31 (s, 18H). ^{13}C NMR ($CDCl_3$, 400MHz): 161.38, 139.87, 137.82, 137.58, 134.51, 133.19, 132.65, 131.52, 131.00, 130.41, 124.56, 115.51, 115.39, 97.86, 90.98, 86.16, 49.46, 33.69.

S,S'-(((9,10-bis(dicyanomethylene)-9,10-dihydroanthracene-2,6-diyl)-bis (ethyne-2,1-diyl))bis(4,1-phenylene)) diethanethioate, TCNAQ

In a dry 50 mL flask under N_2, 89 mg of **6** (0.15 mmol) were dissolved in 25 mL of dry DCM. The solution was placed in an ice bath and 3 mL of acetyl chloride (42 mmol) were added. Then 0.03 mL of $TiCl_4$ (0.32 mmol) were added slowly. The reaction was left under stirring in the ice bath for 3.5 hours, then 20 mL of water were added. The water phase was extracted once with DCM, then the combined organic phases were extracted with water four times. The solutes were adsorbed on silica and the product purified via column cromatography (SiO_2, hexane:ethyl acetate 3:1, R_f= 0.1, slowly increasing to 1:1). The product was finally recrystallized from toluene to form opaque red crystals (47 mg (48% yield). ^{1}H NMR ($CDCl_3$, 400MHz): 8.33 (d, J= 1.6 Hz, 2H), 8.23 (d, J= 8.2 Hz, 2H), 7.84 (dd, J= 1.6, 8.2 Hz, 2H), 7.60 (d, J= 7.9, 4H), 7.45 (d, J= 7.9, 4H), 2.45 (s, 6H). ^{13}C NMR ($CDCl_3$,

400MHz): . Elemental analysis, exp. (theo.): C 73.42% (73.60%), H 3.24% (3.09%), N 8.18% (8.58%), S 9.36% (9.82%), O 5.80% (4.91%).

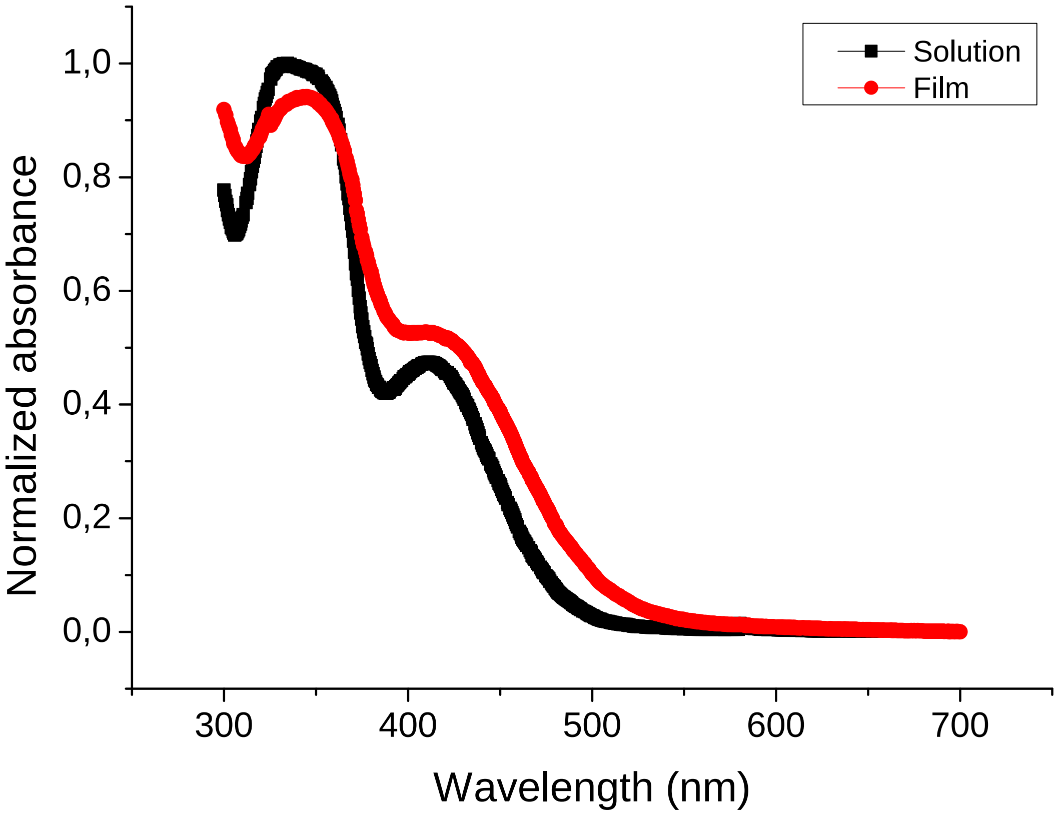

Figure S3: Normalized absorption spectra of **TCNAQ** : 50 μM in $CHCl_3$ (black), and drop-cast thin-film (red).

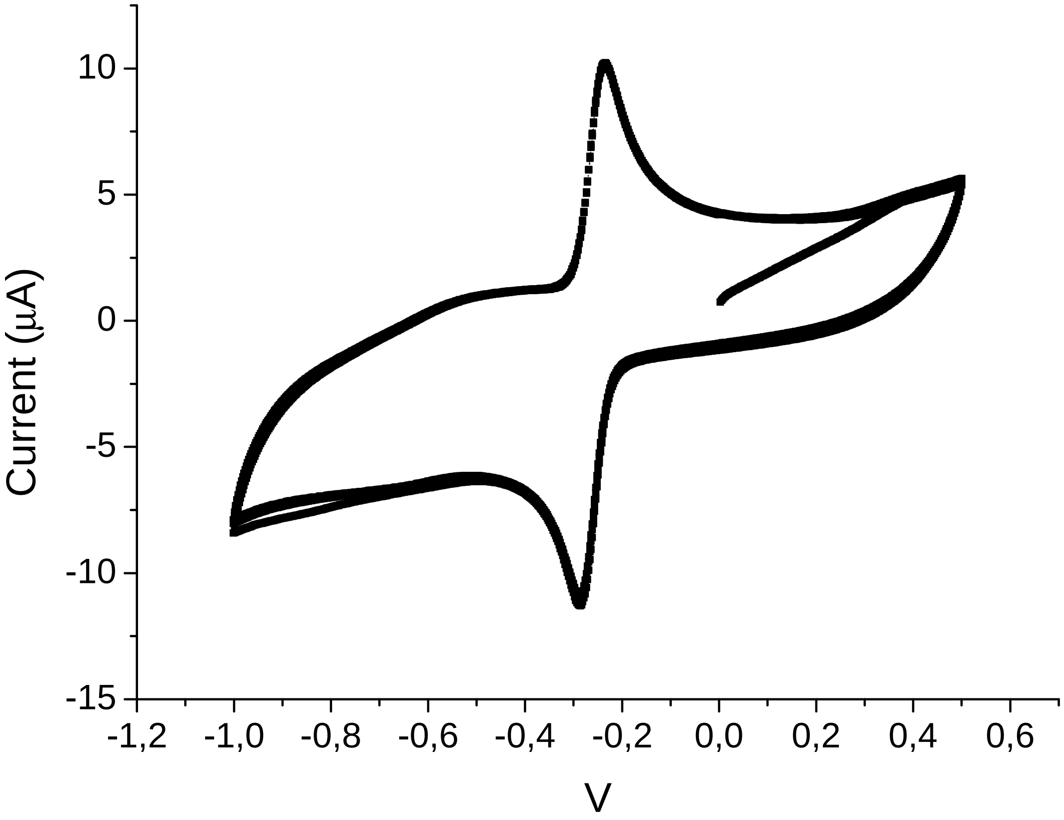

Figure S4: Cyclic voltammetry of **TCNAQ** 0.1 mM in acetonitrile vs. Ag/AgCl. A LUMO energy of -4.3 eV can be extracted from the plot. From the data we can extrapolate a LUMO for **TCNAQ** of -4.5 eV using the procedure reported by Huo *et al.*.[S2]

2 Preparation the SAMs

All the SAMs were prepared under nitrogen atmosphere. For Au-on-mica substrates, the metal surfaces (1x1 cm, 200 nm thick Au, obtained from Phasis, Switzerland) were incubated for two days in a 0.5 mM solutions of **TCNAQ** in dry chloroform to which 0.3 mL of distilled triethylamine were added according to a know procedure.[S1] The SAMs were finally rinsed with dry chloroform and let dry for 20 minutes before the measurement. The same procedure was used to prepare SAMs of **AQ** and **AC**. For template-stripped metal substrates (Au^{TS}, Ag^{TS}, Pt^{TS}), the SAMs were formed by incubating a 1x1 cm template-stripped metal surface (100 nm-thick) overnight in 3 mL of a 50 µmol solution of **TCNAQ** in dry toluene followed by addition of 0.05 mL of 17 mM diazabicycloundec-7-ene (DBU) solution in dry toluene 1.5 hours prior the measurement, according to a known procedure.[S3] The substrates were then rinsed with ethanol and let to dry for 30 minutes before performing the measurements. SAMs of **AC** on Au^{TS} were prepared and measured elsewhere.[S4] SAMs of **C16SH** on Au^{TS} were prepared by immersion of the metal substrates in 3 mL of a 3 mM solution of **C16SH** in degassed ethanol overnight. The samples were then rinsed with ethanol and let to dry for 30 minutes before performing the measurements.

2.1 X-ray Photoelectron Spectroscopy

X-ray Photoelectron Spectroscopy (XPS) of **TCNAQ** SAMs on Au-on-mica and Au^{TS} was performed using a Surface Science SSX-100 ESCA instrument with a monochromatic Al K_α X-ray source ($h\nu$ = 1486.6 eV). The pressure in the measurement chamber was below 1×10^{-9} mbar during data acquisition. The electron take-off angle with respect to the surface normal was 37°. XPS spectra were analyzed using a least-squares curve fitting program (Winspec) developed in the LISE laboratory of the Facultés Universitaires Notre-Dame de la Paix (Namur, Belgium). The diameter of the analyzed area was 1 mm yielding a total experimental energy resolution of 1.1 eV. Deconvolution of the spectra included a

Shirley baseline subtraction and fitting with a minimum number of peaks consistent with the structure of the molecules on a surface, taking into account the experimental resolution.[S5] The profile of the peaks was taken as a convolution of Gaussian and Lorentzian functions. Binding energies are reported ±0.1 eV and referenced to the Au 4f7/2 photoemission peak originating from the substrate, centered at a binding energy of 84 eV.[S6] The uncertainty in the peak intensity determination is 2% for N and 1% S. All measurements were carried out on freshly prepared samples; on each surface 5 points were measured. The data reported for **TCNAQ** powder (Figure S5 and Figure 2 in main text) were measured for a drop cast film on Au^{TS} (from 0.5 mM toluene solution): the blue and the black curves in the deconvolution of the N 1s spectra were ascribed to the cyano groups (399.5 eV) and the molecular 'shake-up' peak (402.4 eV) respectively;[S7] the S 2p spectra shows a single peak at 163.6 eV from the two simmetrically identical thioacetate groups. Similar data were obtained for SAMs of **TCNAQ** on Au-on-mica prepared by deprotection with NEt_3 (reported in the main text, Figure 2): again, in the N 1s spectra we can find evidence of the cyano groups (399.6 eV) and the molecular 'shake-up' peak (402.6 eV) respectively. Anyway, compared to the powder spectra, an additional peak was observed at 398.5 eV that we attributed to an electron rich nitrogen species formed due to the charge transfer from the Au substrate to the **TCNAQ** SAM.[S7] In Figure S6 we show that a single peak fit would necessarily leave a shoulder on the lower energy side while the use of the second peak allows a better fit of the data. The peak at 398.5 eV covers 14% of the total area of the signal. The S 2p spectra of the SAM is characterized by peaks at 164.0 eV and 161.8 eV (with an intensity ratio of 3:2) which characterize the unbound top sulfur and the S-Au bond respectively and that confirms that the molecules are indeed standing upright and bound to the metal.[S4] Presence of unbound thiol and/or disulfide on the surface may contribute to the broad 164.0 eV peak.[S8,S9] In Figure S7 we report the XPS data for SAMs of **TCNAQ** on Au^{TS}: in the N 1s spectra we found all the peaks that we reported previously in the case of the SAM on Au-on-mica with comparable binding energy and an extra peak at 400.8 eV that indicates the existence

of N^+ group that we attributed to residual DBU in the SAMs.[S8] The S 2p spectra shows peaks at 162.2 eV and 161.3 eV for the gold-sulfur bonds at the regular sites and step edges respectively,[S8,S10] and at 164.2 eV and 163.5 eV for disulfide in the SAM and the unbound top thioacetate. **TCNAQ** SAMs on Au^{TS} are of lower quality compared to those on Au-on-mica but did not influence the memory effect.

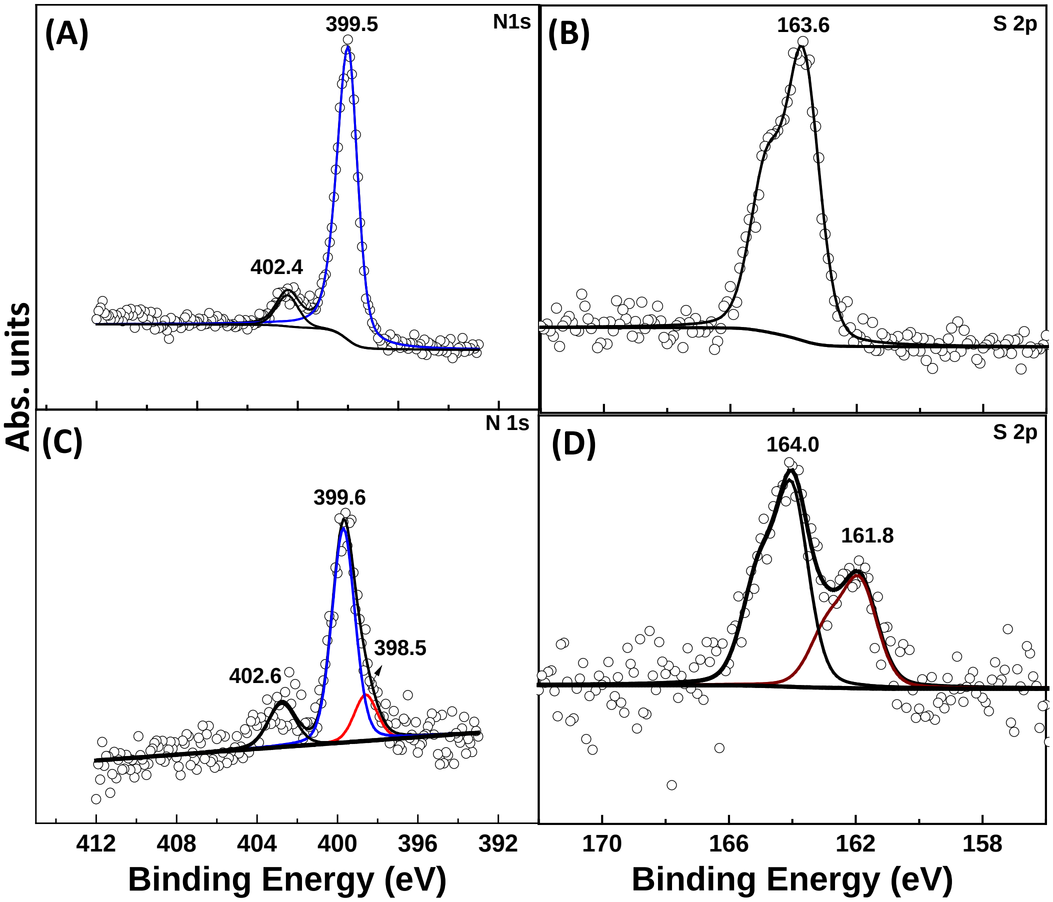

Figure S5: XPS spectra of N 1s (left) and S 2p2/3 (right) core levels for powder samples of **TCNAQ** (top) and SAMs of **TCNAQ** on Au-on-mica (bottom) presented as Figure 2 in the Main Text but showing the raw data points as open circles instead of being connected by a line.

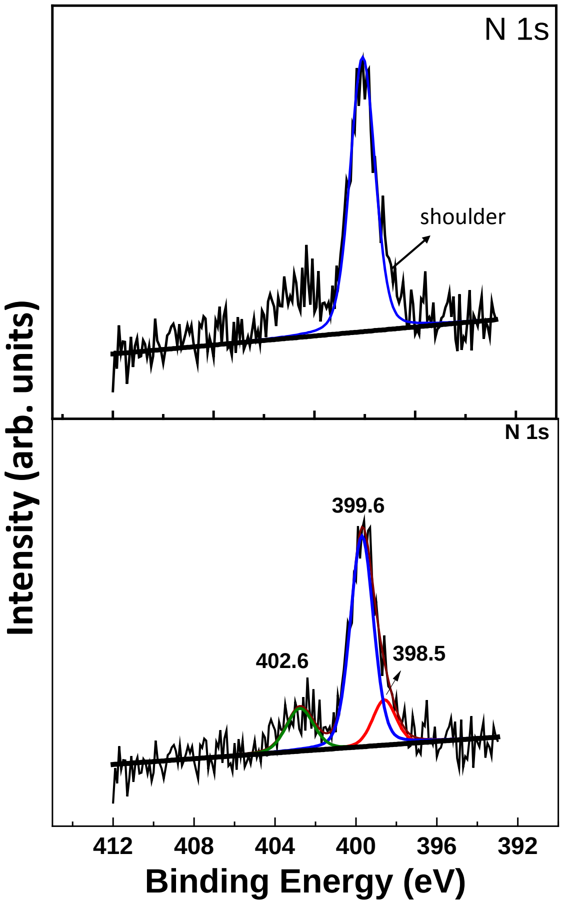

Figure S6: XPS spectra of N 1s core levels for a SAM of **TCNAQ** on Au-on-mica fitted by a different number of peaks: in the top image we fitted the signal with only one peak (blue line), while in the bottom image we used two peaks (blue and red lines; the brown line represents the resulting fit).

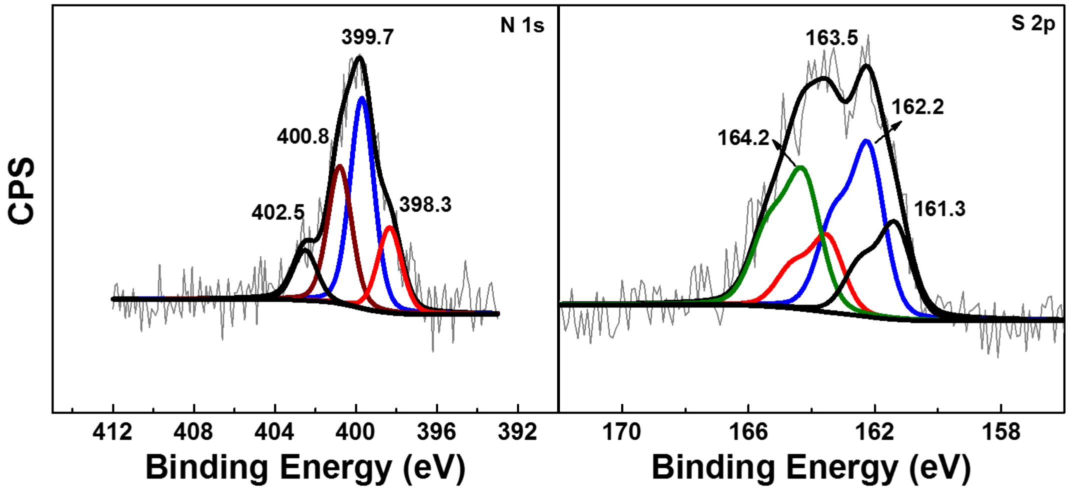

Figure S7: XPS spectra of N 1s and Sr 2p2/3 core levels for SAMs of **TCNAQ** on Au^{TS} deprotected by DBU. N 1s spectra: All other peaks have similar binding energy as shown in Figure 2 of the main text.

2.2 Experiments at the synchrotron

In addition to the laboratory characterization, synchrotron-based XPS and near-edge X-ray fine structure spectroscopy (NEXAFS) measurements were performed on the SAMs of **TCNAQ**; the monolayers were stored in containers for transport to the synchrotron and exposed to ambient before and after the storage so that some degradation cannot be excluded. The experiments were carried out at the HE-SGM beamline (bending magnet) of the synchrotron storage ring BESSY II in Berlin, using a custom-made experimental station. The acquisition of the XPS spectra was conducted with a Scienta R3000 electron energy analyzer, in normal emission geometry, and at an excitation energy of 50 eV. The energy resolution was ~0.3 eV. The binding energy (BE) scale of the spectra was referenced to the Au $4f_{7/2}$ emission at 84.0 eV.[S6] The acquisition of the NEXAFS spectra was carried out at the C K-edges in the partial electron yield mode with a retarding voltage of −50 V. As the primary X-ray source, linearly polarized synchrotron light with a polarization factor of ~88 % was used. The incidence angle of the X-rays, Θ was varied between the normal (Θ =90°) and grazing (Θ =20°) incidence geometry, to monitor the linear dichroism effects reflecting molecular orientation in the SAMs.[S11] The photon energy (PE) scale was referenced to the pronounced $\pi*$ resonance of highly oriented pyrolytic graphite at 285.38 eV.[S12] Raw NEXAFS spectra were normalized to the incident photon flux by division through a spectrum of a clean, freshly sputtered gold sample and subsequently reduced to the standard form.[S11]

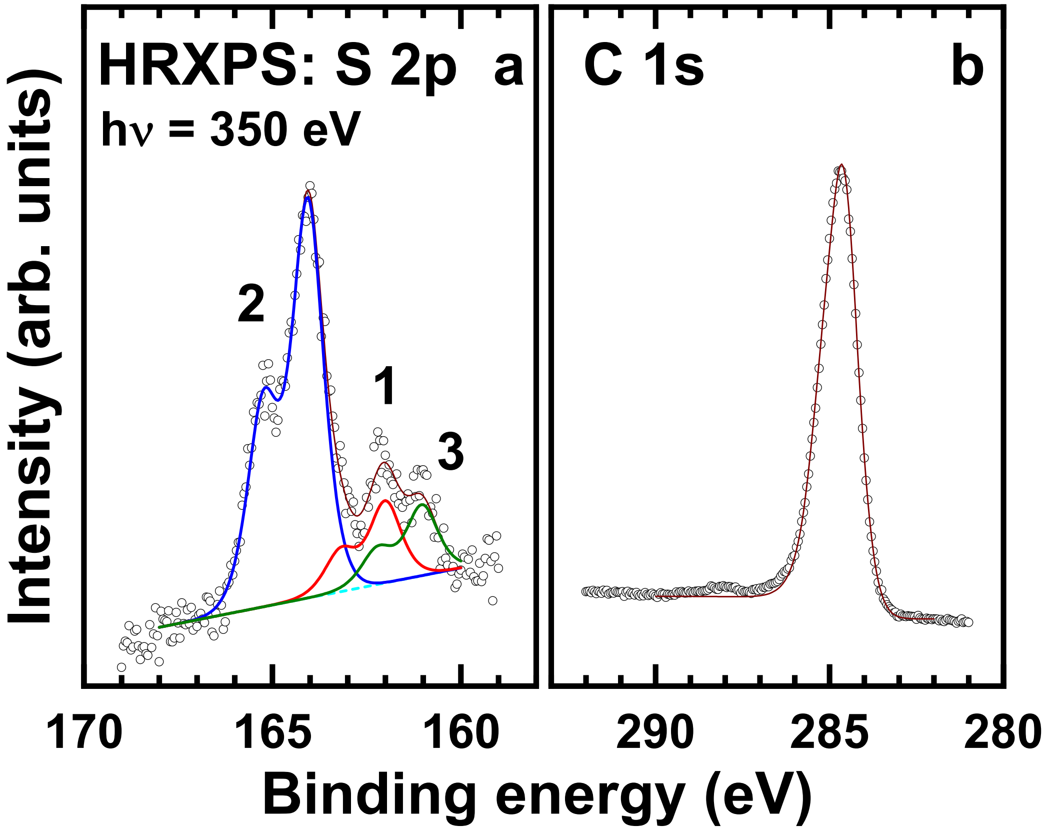

Figure S8: Synchrotron-based S 2p (a) and C 1s (b) XPS spectra of **TCNAQ** on Au. The S 2p spectrum is decomposed into individual doublets, drawn by different colors and marked by numbers (see text for details).

The S 2p (a) and C 1s (b) XPS spectra of **TCNAQ** on Au are presented in Figure S8. The S 2p spectrum in Figure S8a exhibits three doublets associated with thiolate bound to Au (at 162.0 eV; **1**), unbound S (at 164.0 eV; **2**), and atomically bound S (at 161.0 eV; **3**).[S13] The presence of doublet **1** confirms the bonding and SAM-like assembly of **TCNAQ** on Au. The much lower intensity of this doublet as compared to **2** is related to the strong attenuation of the photoelectron signal for the "buried" thiolate groups at the given kinetic energy of photoelectrons (∼180 eV),[S14] suggesting, at the same time, an upright molecular orientation. The presence of doublet **3** is presumably related to contamination or breakage of S-C bonds in some of the adsorbed molecules. The C 1s spectrum in Figure S8b exhibits a slightly asymmetric single peak associated with the molecular backbone of **TCNAQ**. The slight asymmetry is presumably related to the contribution of the nitrile group.[S15,S16] The

N 1s spectrum (not shown) exhibits a single peak at 399.5 eV, associated most likely with the contribution of the nitrile group.

Based on the C1s/Au4f and S2p(thiolate)/Au4f intensity ratios, the effective thickness and the packing densities of the **TCNAQ** monolayer on Au were calculated using standard procedures.[S17,S18] For the thickness evaluation, a standard expression for the attenuation of the photoemission signal was assumed[S14] and the literature values for the attenuation lengths were used.[S19] The spectrometer-specific coefficients were determined by using an octadecanethiolate (C18) SAM on Au as a reference, relying on the well-known thickness of this monolayer (20.9(1) Å). In a similar fashion, this SAM (4.63×10^{14} moleculescm^{-2})[S20] served as a reference for the packing densities evaluation. According to the above evaluation, the effective thickness and the packing densities of the **TCNAQ** monolayer on Au were estimated at 18.7(4) Å and 2.3×10^{14} moleculescm^{-2}, respectively.

The C K-edge NEXAFS spectra of the **TCNAQ** monolayer on Au acquired at X-ray incidence angles of 90°, 55°, and 20° are shown in Figure S9. These spectra exhibit a variety of characteristic absorption resonances. A π_1* resonance **1** at ~285.0 eV is related to the molecular backbone of **TCNAQ** which is, presumably, also the case for π_2* resonance **3** at 287.4 eV.[S11,S15,S16] The $\sigma*$-like resonances **4-6** at 289.0 eV, 293.0 eV and 303.0 eV are related to the molecular backbone, exhibiting a typical pattern for monomolecular films.[S15,S16] Most interesting is however resonance **2** at 286.5 eV, which can be unequivocally ascribed to the nitrile groups of **TCNAQ**,[S15,S16] supporting thus the intact character of the adsorbed molecules. Significantly, the intensity of this $\pi*$ resonance is higher at the grazing incidence as compared to the normal one, which, in view of the orientation of the respective $\pi*$ orbitals (perpendicular to the C-N bond), suggests that the nitrile groups of **TCNAQ** are predominantly oriented parallel to the substrate. Accordingly, the molecular backbones should be oriented upright. Since the other resonances in Figure S9, and especially the most pronounced resonance **2**, do not reveal much dichroism, the average molecular inclination in the **TCNAQ** film is presumably close to 35°, which is 90° to 55° (magic angle).

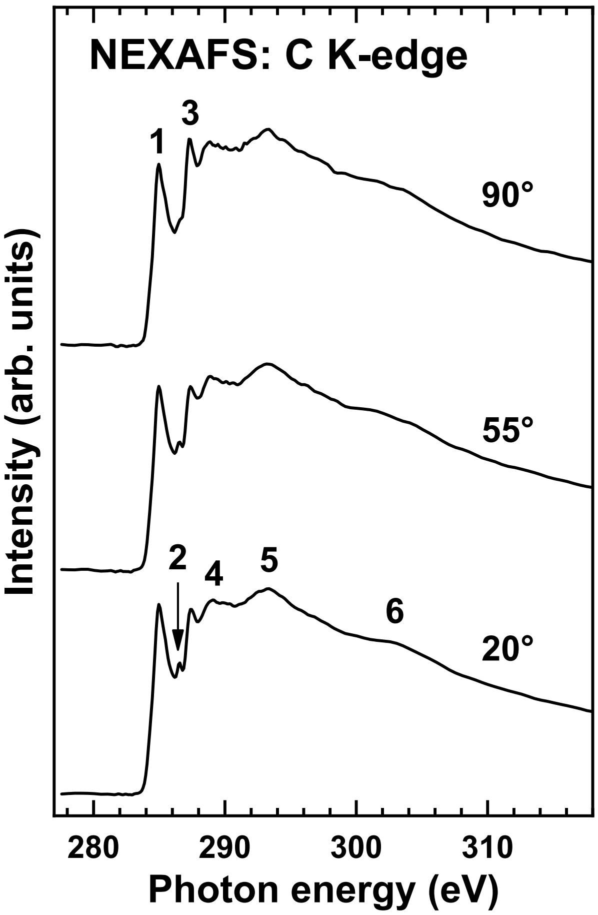

Figure S9: C K-edge NEXAFS spectra of the **TCNAQ** monolayer on Au acquired at X-ray incidence angles of 90°, 55°, and 20°. The spectra are shifted vertically for comparison. Characteristic absorption resonances are marked by numbers (see text for details).

3 Acquisition and processing of data

3.1 $J-V$ curves

All the electrical characterizations were performed in a controlled nitrogen atmosphere containing 1-3% O_2 and RH< 15%. The details of such EGaIn setup are described elsewhere.[S3] For SAMs of **TCNAQ** on Au^{TS} or Au-on-mica, 3 and 4 substrates were prepared respectively, and at least 5 AuTS/SAM//Ga_2O_3/EGaIn junctions per sample were measured (5 scans from $0V \rightarrow 1V \rightarrow -1V \rightarrow 0V$, steps of 0.05 V, 0.1 s delay between steps) for a total of at least 25 traces per sample. A new EGaIn tip was prepared every 3-5 junctions and flattened by gently pushing it on a Si wafer a few times according to the procedure reported by Simeone et al.[S21] A similar procedure was used for all the other systems: for **TCNAQ** SAMs on Ag^{TS} or Pt^{TS}, 2 samples were measured with 10 junction per sample; for SAMs of **AQ** and **AC** on Au-on-mica, 3 sample were prepared and at least 15 junctions per sample were measured; for SAMs of **C16SH** on Au^{TS}, 2 sample were prepared and at least 5 junctions per sample were measured. Data for **AC** on Au^{TS}were collected and analyzed somewhere else.[S4] These data were parsed in a "hands-off" manner using Scientific Python to produce histograms of J for each value of V , the associated Gaussian fits (using a least-squares fitting routine) as described elsewhere.[S4] Data for different molecule/metal system are summarized in Figure S10 and in Table S1; Figures S11-S25 show characteristic traces obtained during the above mentioned bias sweeps for the different systems. As we can observe, the hysteresis loop is present only in the case of junctions comprising SAMs of **TCNAQ**. The yield was calculated as number of junctions that did not produce a short circuit during the complete scanning cycles over the total number of probed junctions. We prepared mixed monolayers of **C16SH/TCNAQ** on Au^{TS} by immersing a freshly prepared sample of **C16SH** in 3 mL of a 50 µmol solution of **TCNAQ** in dry toluene followed by addition of 0.05 mL of 17 mM diazabicycloundec-7-ene (DBU) solution in dry toluene 1.5 hours prior the measurement as reported previously for SAMs of pure **TCNAQ**. When

mixed monolayers **C16SH/TCNAQ** were prepared, the resulting junctions appeared to be more conductive compared to SAMs of pure **C16SH** but no hysteresis or memory effect were recorded (Figure S26)

To produce forward trace over reverse trace ratios (F/R, Fig. 4 in the main text), 10 junctions per system (5 traces per junction) were randomly selected and each forward trace was divided by the previous reverse trace to produce an array of values of F/R for each potential point that was used to compute the mean values. The error was presented as confidence interval ($\alpha = 0.05$), calculated from the standard deviation of these averages using 9 as degrees of freedom. The reason why we chose to take 10 random junctions for each system despite the fact that more were measured has to do with the comparison of the data sets between different molecules/substrates: increasing the number of junctions doesn't affect much the mean but reduces the error bars considerably, by plotting the same number of junctions for each examined system we can visually show the distribution of data in the set of a particular molecule/substrate system.

Table S1: Summary of EGaIn measurements data.

	Yield	N of junct.	Traces	LogJ(0.5 V)	V_{trans}+	V_{trans}-
**TCNAQ** Au^{TS}	93%	15	75	-2.2 ± 0.2	0.53 ± 0.03	
**TCNAQ** Au mica	62%	45	225	-2.7 ± 0.2	0.51 ± 0.12	
**TCNAQ** Ag^{TS}	100%	15	75	-2.4 ± 0.2	0.31 ± 0.05	
**TCNAQ** Pt^{TS}	58%	15	75	-2.4 ± 0.2	0.56 ± 0.10	-0.50 ± 0.07
**AC** Au mica	61%	68	340	-2.5 ± 0.1	0.63 ± 0.14	-0.84 ± 0.12
**C16SH** Au^{TS}	94%	17	85	-4.1 ± 0.2	0.60 ± 0.11	-0.75 ± 0.18
**AQ** Au mica	97%	34	170	-4.10 ± 0.2	0.21 ± 0.09	-0.28 ± 0.09

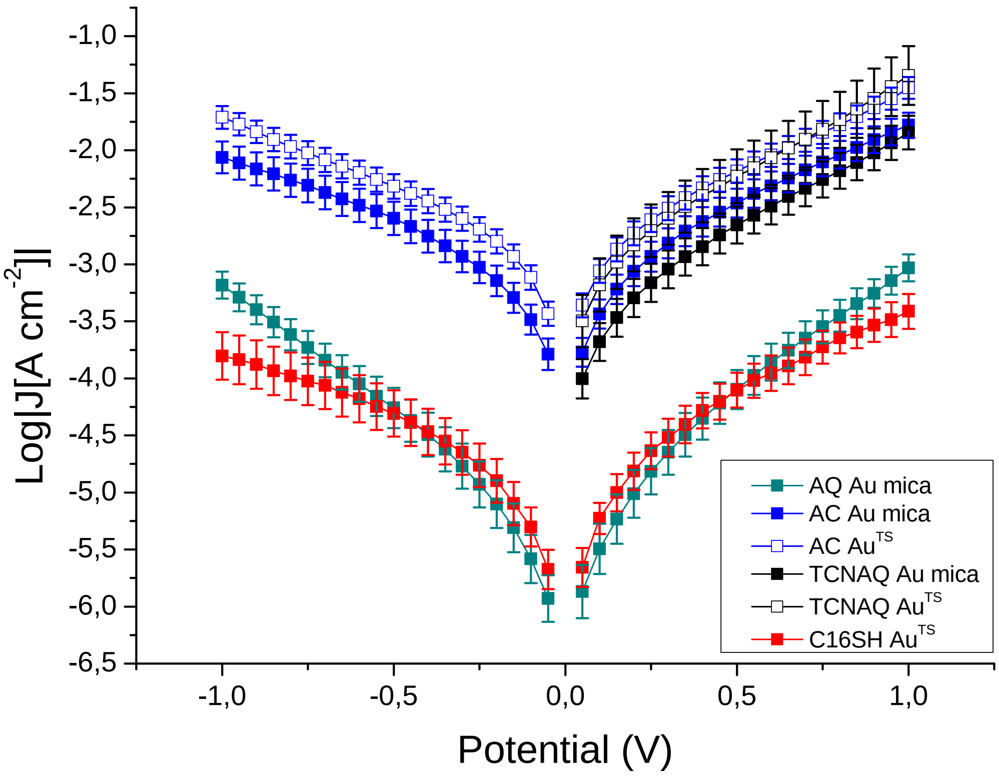

Figure S10: Gaussian fit of Log—J— vs. V plots for M/SAM//EGaIn junctions comprising SAMs of: **TCNAQ** on Au-on-mica (solid black), **TCNAQ** on Au^{TS}(hollow black), **AC** on Au-on-mica (solid blue), **AC** on Au^{TS} (hollow blue), **AQ** on Au-on-mica (cyan), and **C16SH** on Au^{TS}. For **TCNAQ** only the data points collected at positive bias are reported because of the hysteresis characterizing the electrical properties at negative bias. The error bars represents confidence intervals with $\alpha = 0.05$.

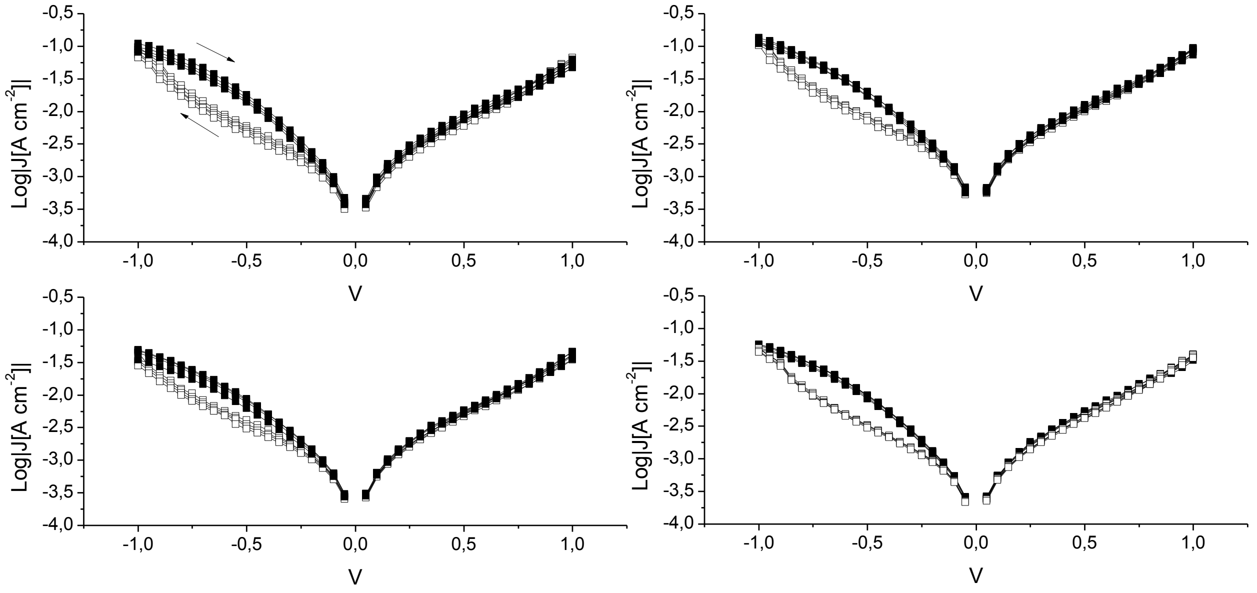

Figure S11: Examples of $Log|J|$ vs. V traces for $\mathrm{Au}^{\mathrm{TS}}$/**TCNAQ**//EGaIn junctions. Solid symbols represent forward traces from $-1V$ to $+1V$, hollow symbols reverse traces from $+1V$ to $-1V$.

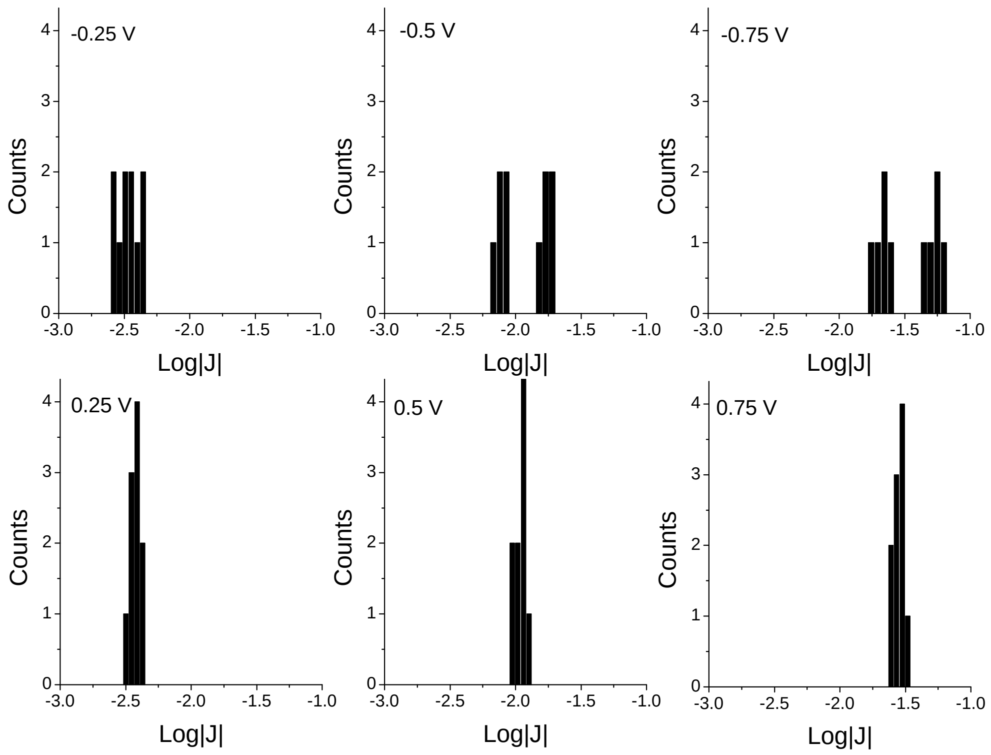

Figure S12: Examples of $Log|J|$ histograms at -0.75 V, -0.5 V, -0.25V, 0.25 V, 0.5 V, and 0.75 V for $\mathrm{Au}^{\mathrm{TS}}$/**TCNAQ**//EGaIn junctions.

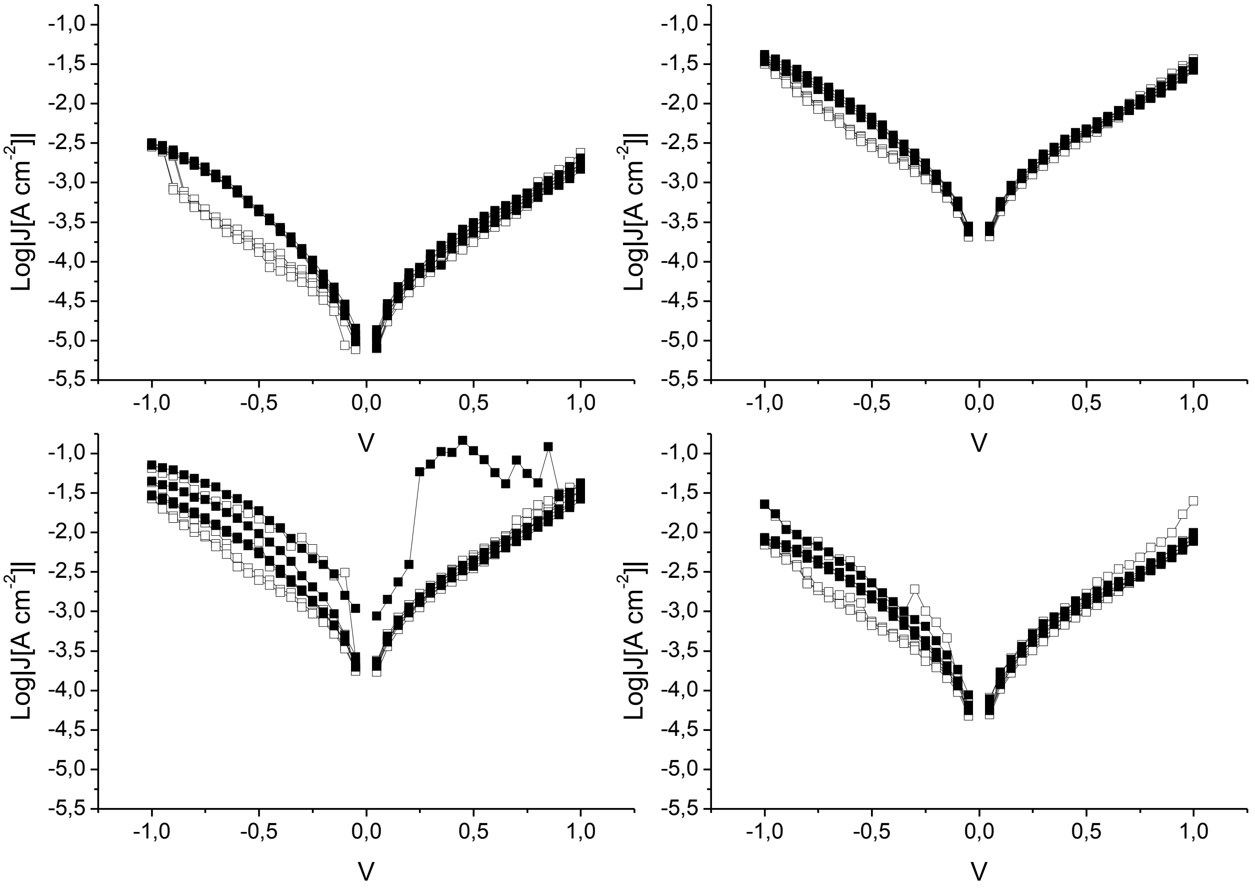

Figure S13: Examples of $Log|J|$ vs. V traces for Au-on-mica/**TCNAQ**//EGaIn junctions. Solid symbols represent forward traces from $-1V$ to $+1V$, hollow symbols reverse traces from $+1V$ to $-1V$.

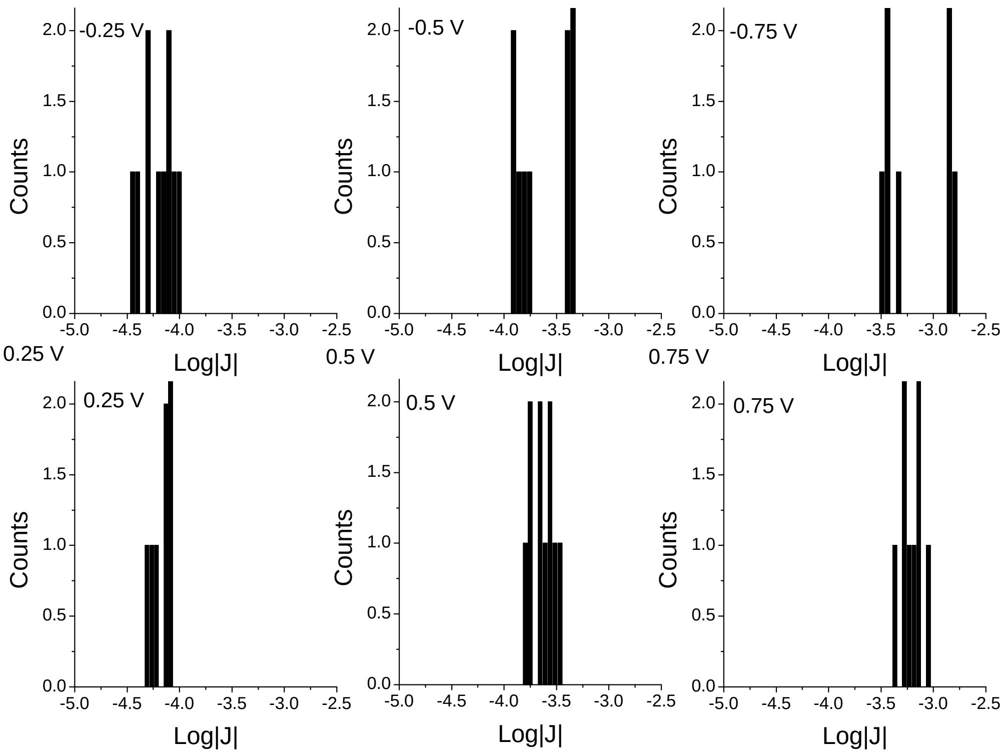

Figure S14: Examples of $Log|J|$ histograms at -0.75 V, -0.5 V, -0.25V, 0.25 V, 0.5 V, and 0.75 V for Au-on-mica/**TCNAQ**//EGaIn junctions.

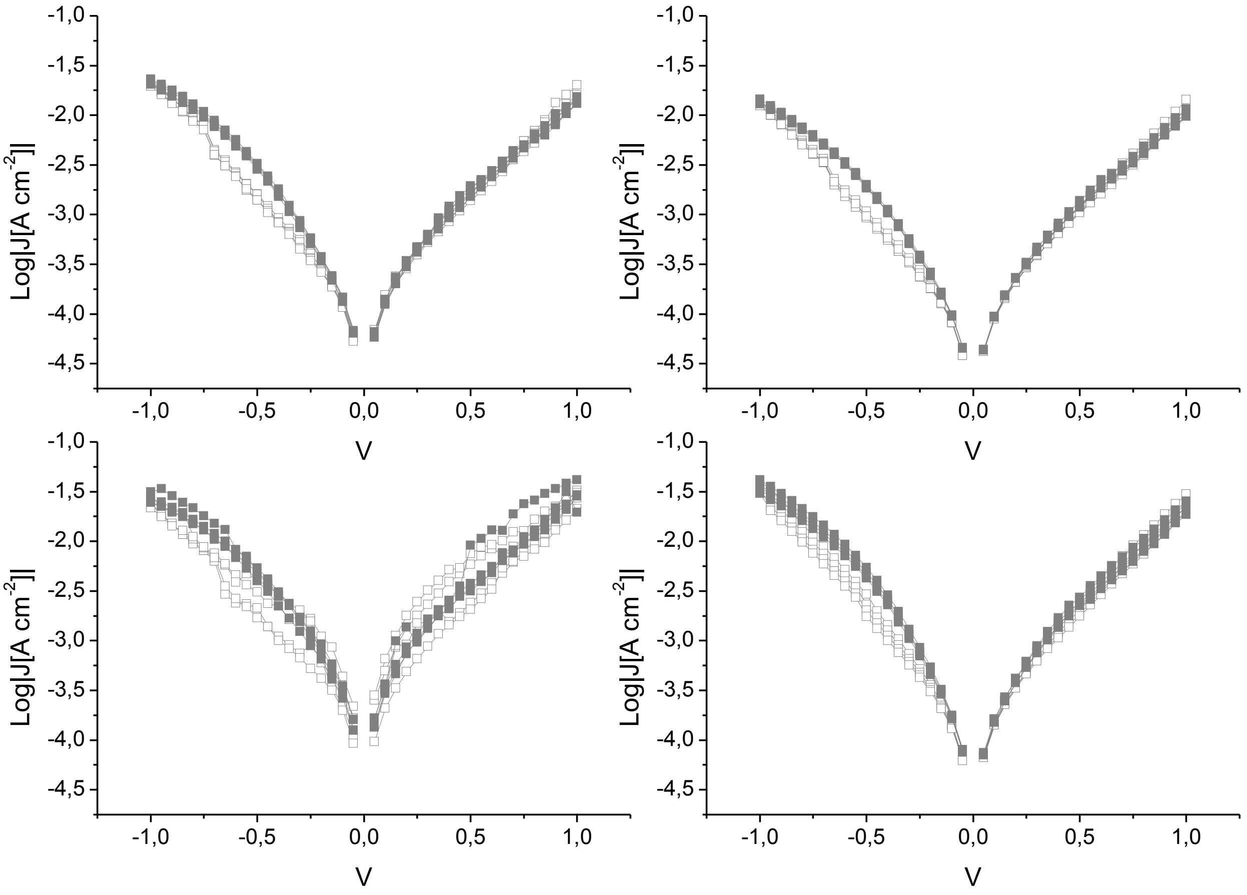

Figure S15: Examples of $Log|J|$ vs. V traces for Ag^{TS}/**TCNAQ**//EGaIn junctions. Solid symbols represent forward traces from $-1V$ to $+1V$, hollow symbols reverse traces from $+1V$ to $-1V$.

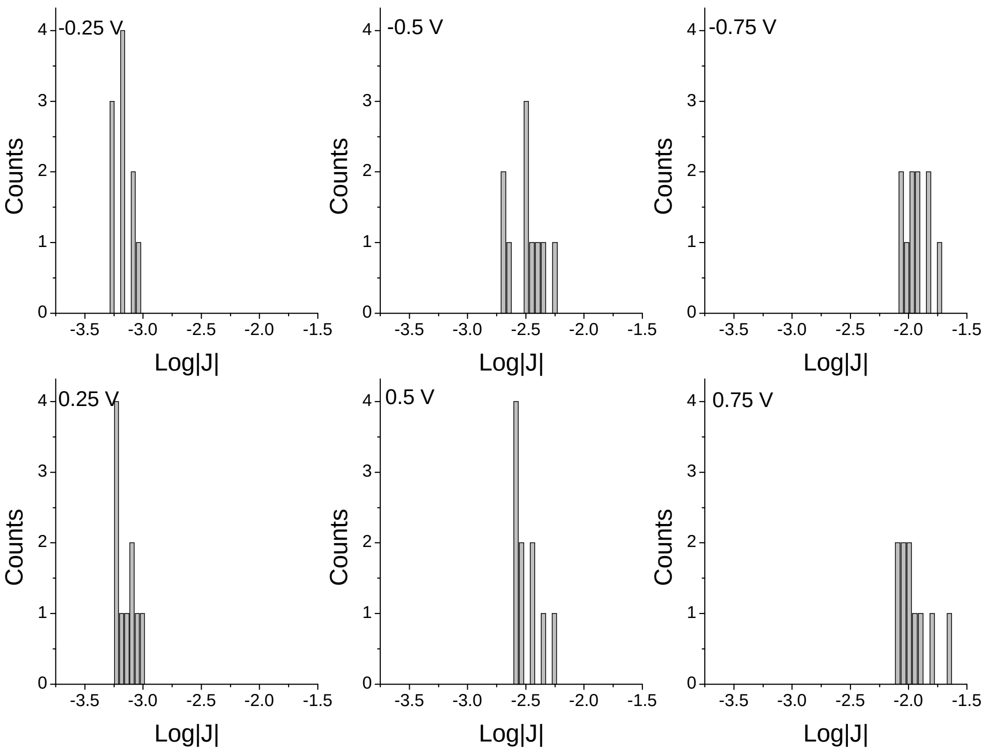

Figure S16: Examples of $Log|J|$ histograms at -0.75 V, -0.5 V, -0.25V, 0.25 V, 0.5 V, and 0.75 V for Ag^{TS}/**TCNAQ**//EGaIn junctions.

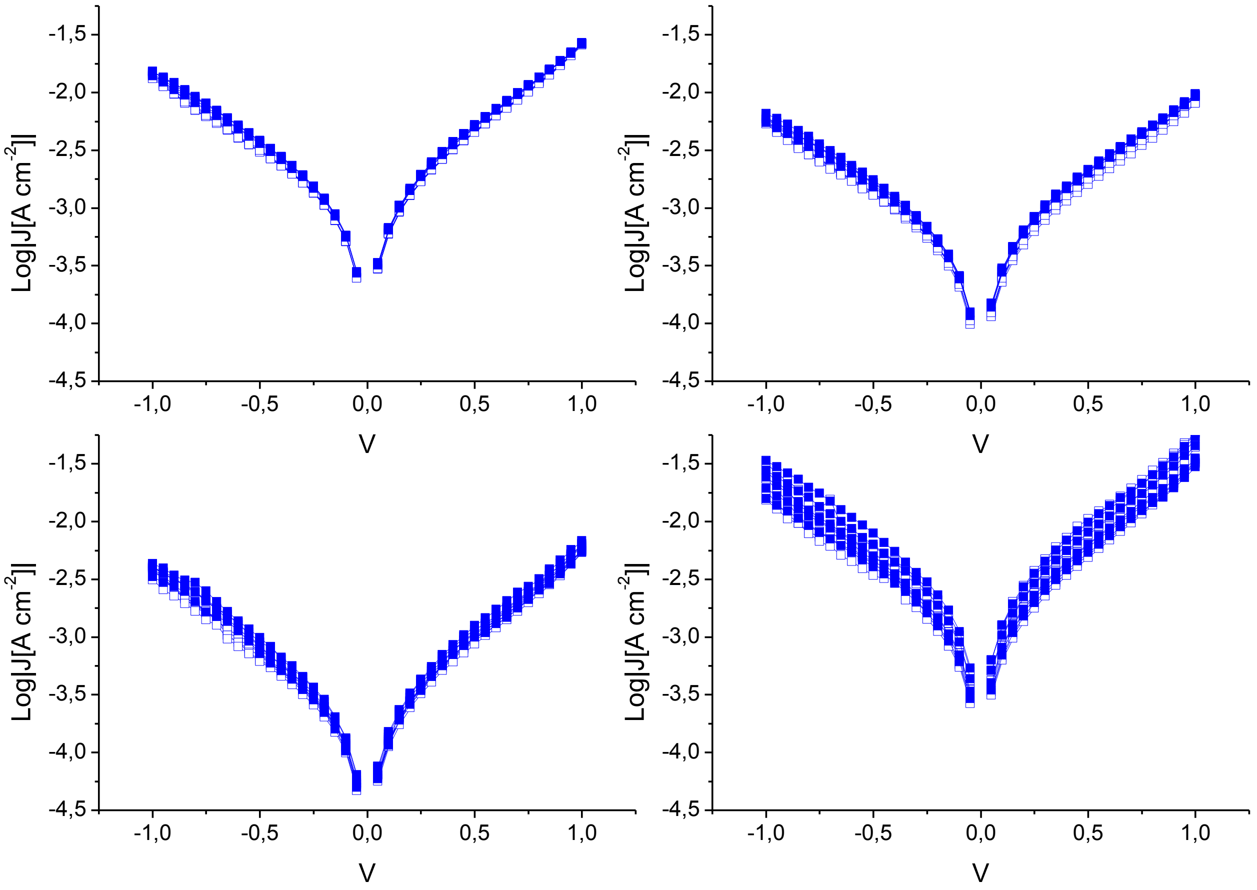

Figure S17: Examples of $Log|J|$ vs. V traces for Au^{TS}/**AC**//EGaIn junctions. Solid symbols represent forward traces from $-1V$ to $+1V$, hollow symbols reverse traces from $+1V$ to $-1V$.

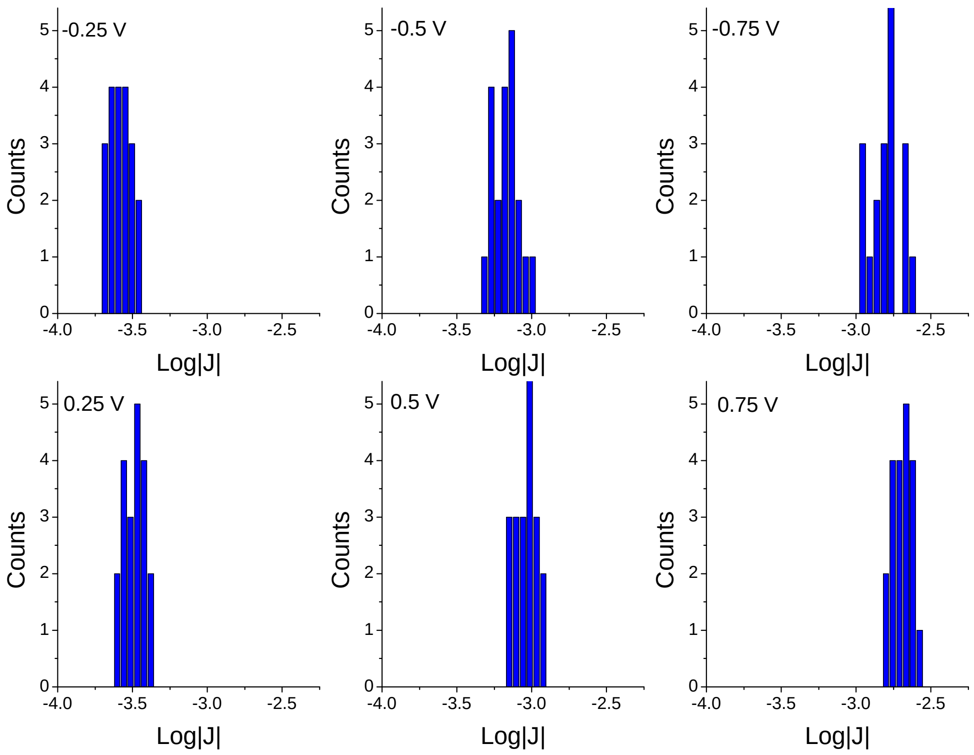

Figure S18: Examples of $Log|J|$ histograms at -0.75 V, -0.5 V, -0.25V, 0.25 V, 0.5 V, and 0.75 V for $\mathrm{Au}^{\mathrm{TS}}$/**AC**//EGaIn junctions.

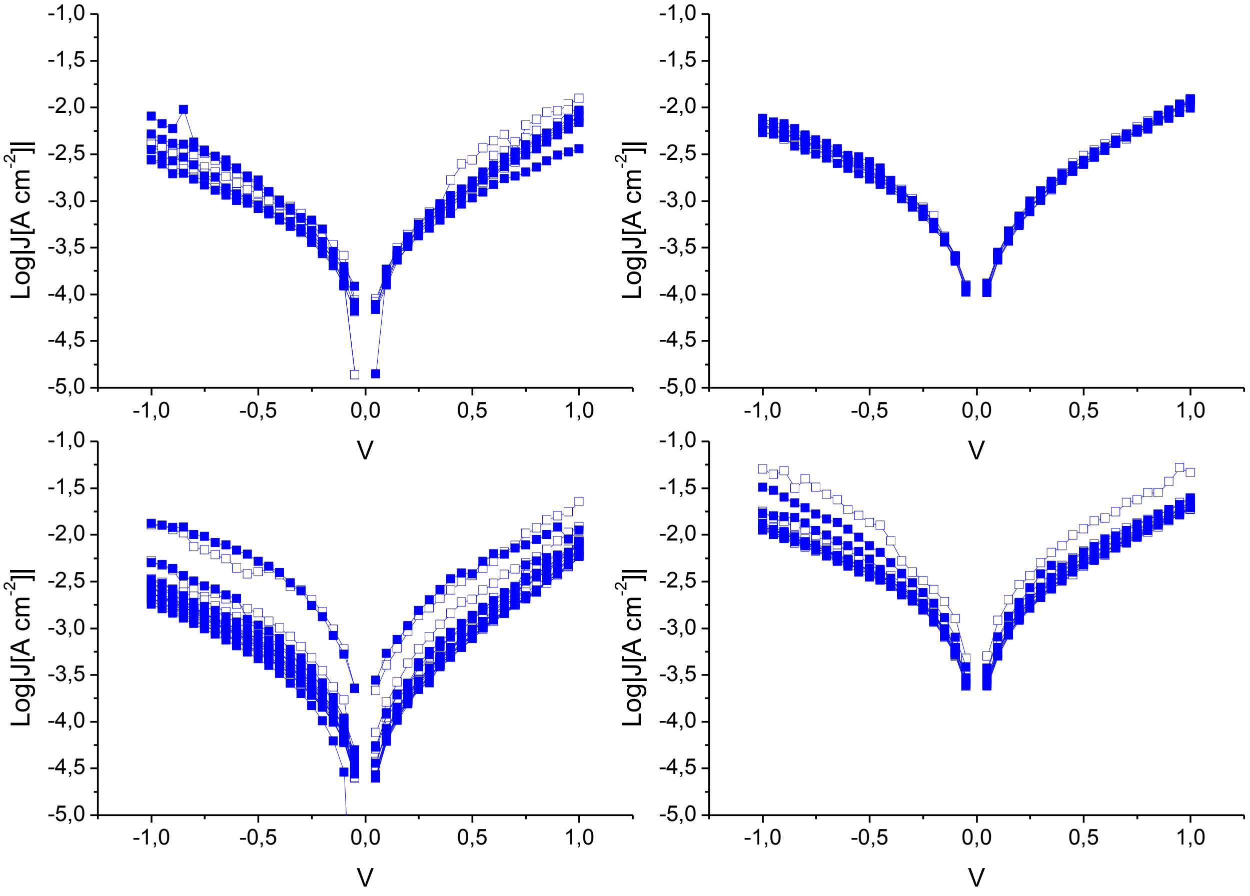

Figure S19: Examples of $Log|J|$ vs. V traces for Au-on-mica/**AC**//EGaIn junctions. Solid symbols represent forward traces from $-1V$ to $+1V$, hollow symbols reverse traces from $+1V$ to $-1V$.

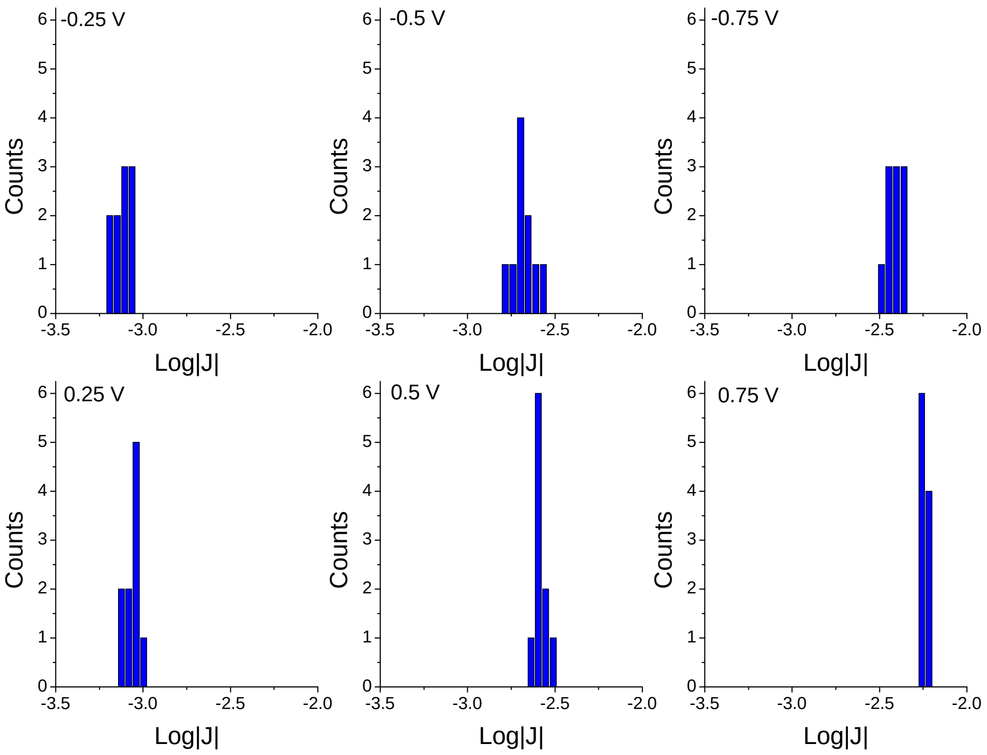

Figure S20: Examples of $Log|J|$ histograms at -0.75 V, -0.5 V, -0.25V, 0.25 V, 0.5 V, and 0.75 V for Au-on-mica/**AC**//EGaIn junctions.

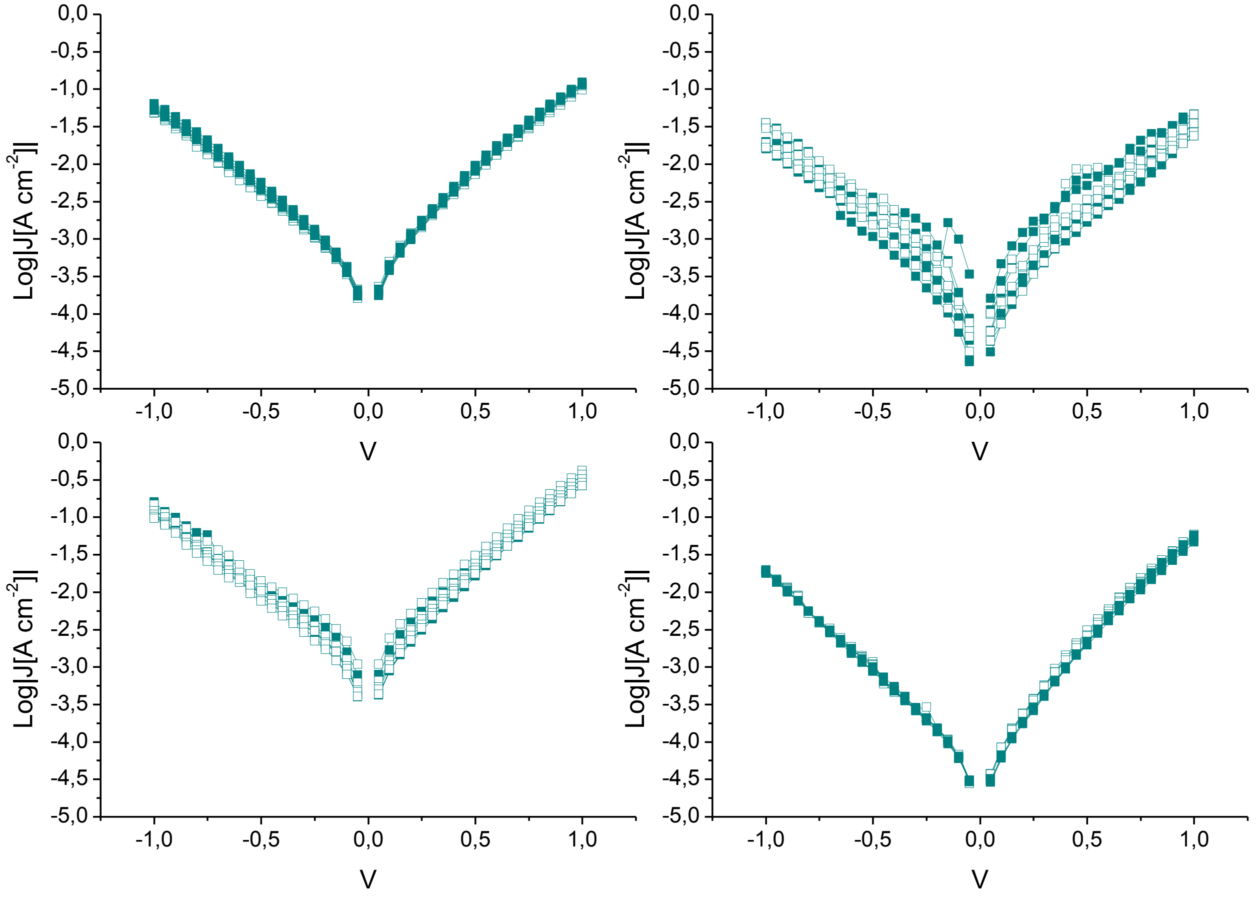

Figure S21: Examples of $Log|J|$ vs. V traces for Au-on-mica/**AQ**//EGaIn junctions. Solid symbols represent forward traces from $-1V$ to $+1V$, hollow symbols reverse traces from $+1V$ to $-1V$.

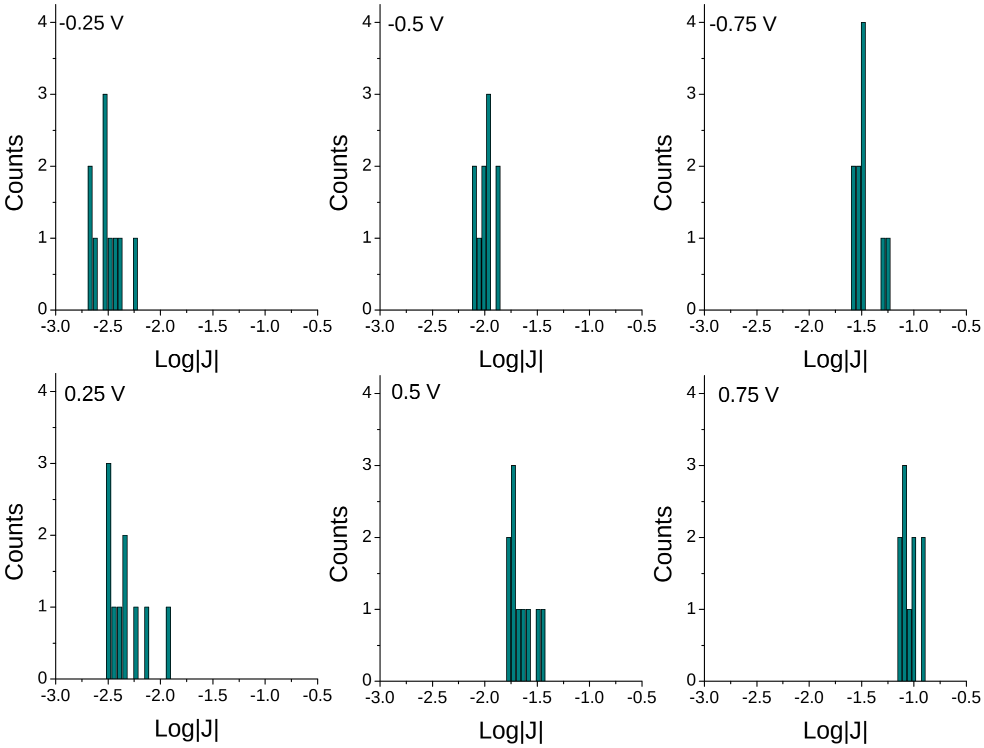

Figure S22: Examples of $Log|J|$ histograms at -0.75 V, -0.5 V, -0.25V, 0.25 V, 0.5 V, and 0.75 V for Au-on-mica/**AQ**//EGaIn junctions.

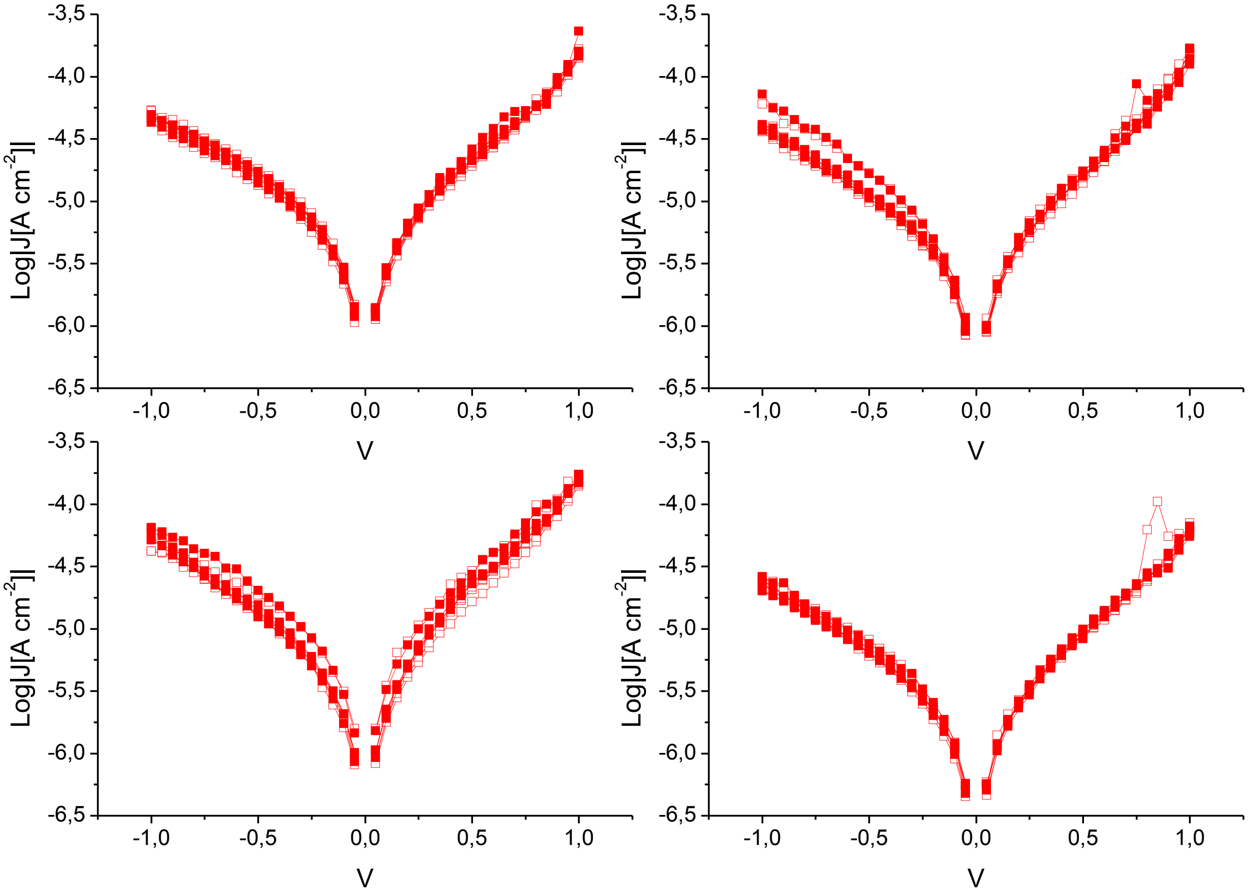

Figure S23: Examples of $Log|J|$ vs. V traces for Au^{TS}/**C16SH**//EGaIn junctions. Solid symbols represent forward traces from $-1V$ to $+1V$, hollow symbols reverse traces from $+1V$ to $-1V$.

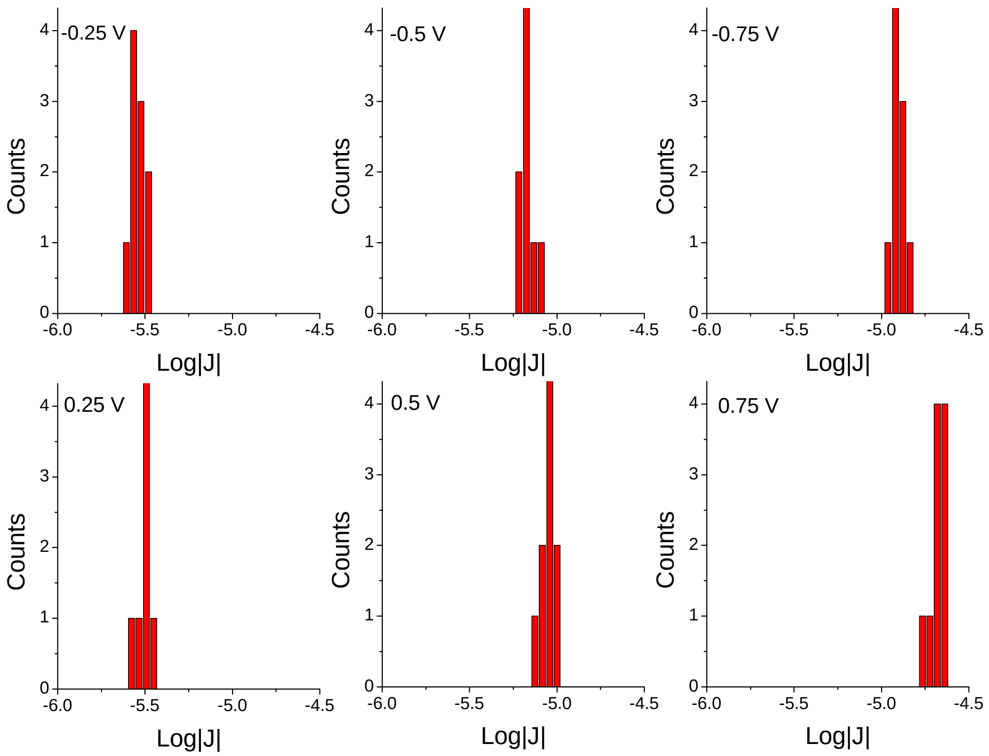

Figure S24: Examples of $Log|J|$ histograms at -0.75 V, -0.5 V, -0.25V, 0.25 V, 0.5 V, and 0.75 V for Au^{TS}/**C16SH**//EGaIn junctions.

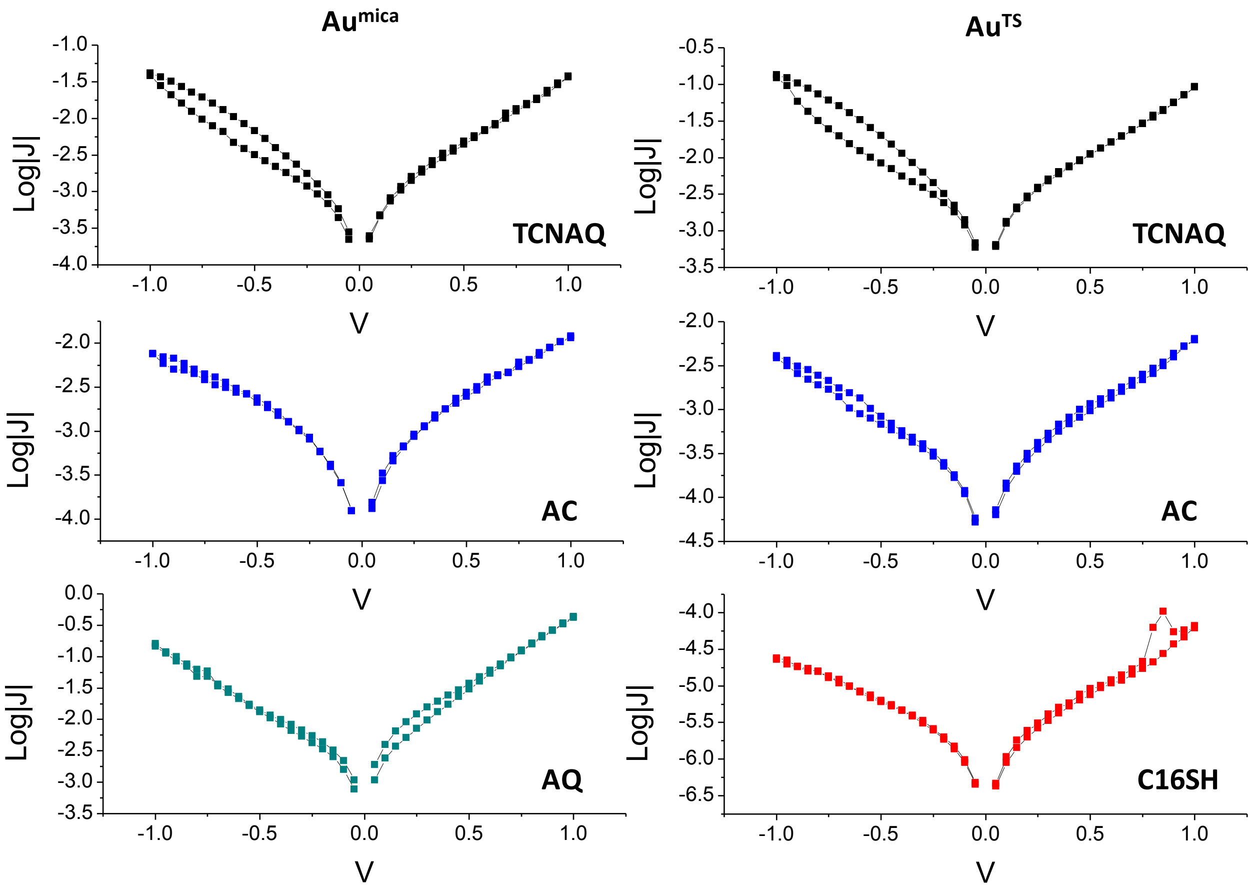

Figure S25: Examples of single $Log|J|$-V traces for different Au/SAM//EGaIn junctions acquired from $0V \rightarrow 1V \rightarrow -1V \rightarrow 0V$ with steps of 0.05 V.

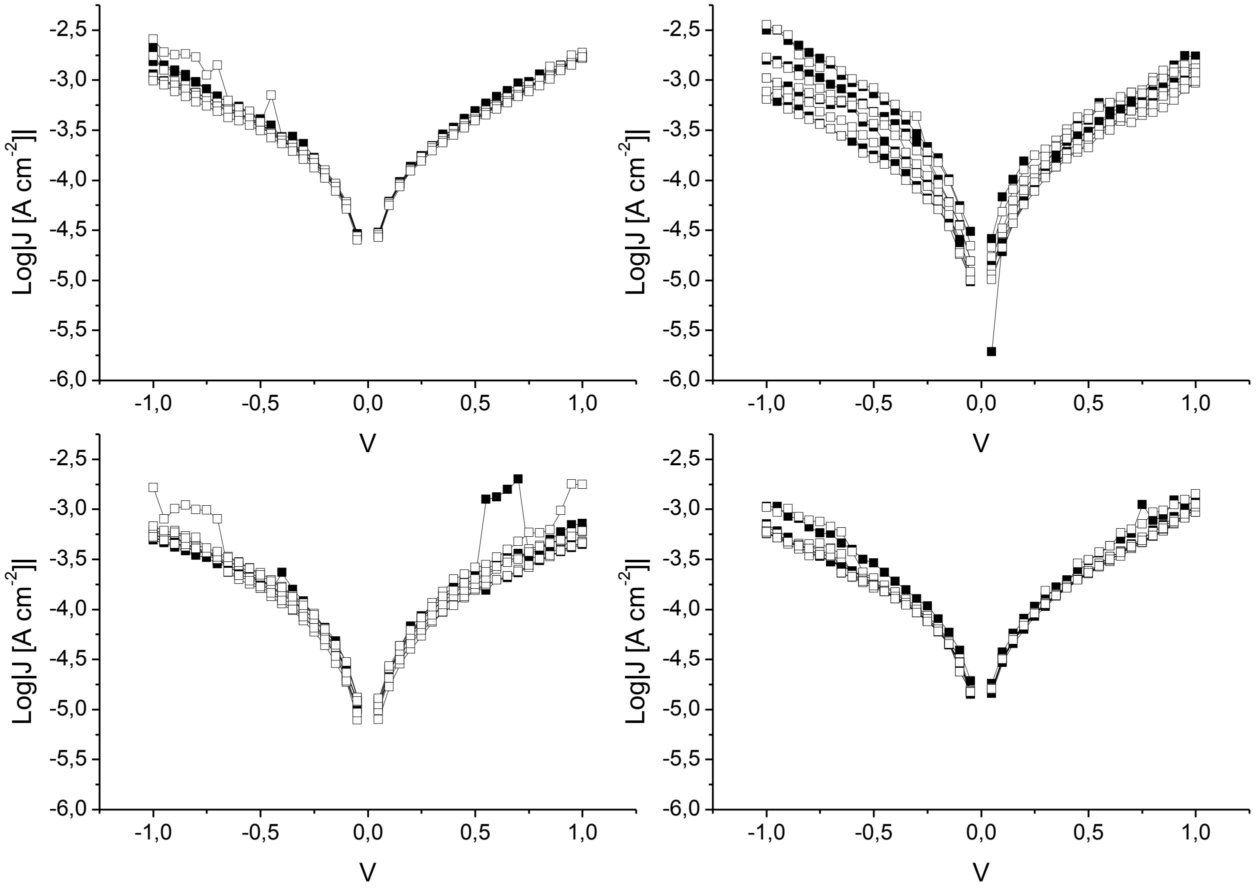

Figure S26: Examples of $Log|J|$ vs. V traces for Au^{TS}/SAM//EGaIn junctions comprising mixed-monolayers of **C16SH** and **TCNAQ**. Solid symbols represent forward traces from $-1V$ to $+1V$, hollow symbols reverse traces from $+1V$ to $-1V$.

3.2 Write-Read-Erase-Read (WRER) cycles

The WRER cycles measurements were carried on the same setup introduced in the previous subsection. A flattened EGaIn tip was brought in contact with the SAM and a bias of +1 V was applied for 30 seconds. This 'burning' operation was necessary in order to produce comparable results between different junctions: when not 'burned', the junctions gave extremely high but not reproducible ON/OFF ratios during the first 3-4 cycles (with values that spanned from 10 to more than 100); after 'burning', the junctions behaved in a much more similar manner and those were used to produce the data. This points to the fact that in principle **TCNAQ** SAMs could be used to prepare devices with higher ON/OFF ratios than the ones reported in this study, but in this study we aim to characterize this effect using elements of statistical analysis. The WRER operations were performed by applying specific biases for certain periods of time and measuring the current: Read (R_{OFF}, R_{ON}; -0.5 V, 5 s); Write (W; -1.5 V, 10 s); Erase (E; +1 V, 10 s). After the initial 'burning', the junctions were found in the low conductance (OFF) state and cycles of WRER operations were carried on the same junction in the order $R_{OFF} \rightarrow W \rightarrow R_{ON} \rightarrow E$. Reads were carried on for 5 seconds, taking one point every 0.1 seconds, to ensure that the current was stable and not drifting with time; the reading bias was chosen observing the hysteresis loops obtained in the J-V plots. Erase and Write pulses were carried on for 10 seconds to allow the current at +1 V and -1.5 V to stabilize: surprisingly, changing the duration of the pulses down to 2.5 seconds did not affect the ON/OFF ratio, but the chances of shorting the junction in the latter scenario increased. The junctions were cycled for 20 times, after which the ON/OFF ratio usually dropped below 1.7, a value that we considered unsatisfactory. Examples of the obtained R_{ON} and R_{OFF} data are reported in Figure S27 - S32: as it is possible to observe, both the ON and OFF currents tend to decrease with the number of cycles, the stability and variation of the absolute values of current in the junctions during the WRER cycles will be discussed in the next section. Junctions that produced a short before the 5th cycle (including during the burning) were considered non-working junctions and not included in the

analyzed data. A summary of the yields and the number of junction measured per different molecule/substrate system are summarized in Table S2. A new EGaIn tip was formed every 3 working junctions or 3 shorts. Only 5 junctions (on 2 samples) were collected for **AC**, **AQ**, and **C16SH** as these measurements are time consuming and no effect was expected by looking at $J - V$ curves.

Table S2: Summary of EGaIn junctions used for the WRER experiments.

	WRER Yield	Number of junctions
**TCNAQ** Au^{TS}	44%	31
**TCNAQ** Au mica	39%	17
**TCNAQ** Ag^{TS}	20%	10
**TCNAQ** Pt^{TS}	56%	5
**AC**	71%	5
**C16SH**	83%	5
**AQ**	60%	6

For every junction measured, to calculate the ON/OFF ratio for each n cycle, an array of values was created by dividing each single value of current recorded during the nth R_{ON} (50 values in 5 seconds) by every single value of current during the nth R_{OFF} (*i.e.* for each n cycle of a junction the array contained 50x50=2500 ON/OFF values). For each n cycle measured on different junctions of the same SAM/substrate system, we combined the aforementioned arrays and computed the mean and the standard deviation to obtain the final ON/OFF ratio. The error was presented as confidence interval ($\alpha = 0.05$), calculated from the standard deviation of these averages using the number of junctions - 1 as degrees of freedom. The ON/OFF ratio values obtained with the method mentioned above are comparable with those that can be more simply obtained by dividing for every n^{th} cycle the mean values of all the R_{ON} operations by the mean value of all the R_{OFF} operations but the aforementioned treatment is more rigorous. In Figure S33, we report the value of the ON/OFF ratio for 4 junctions in which a bias of $-1V$ was used to perform the W operation.

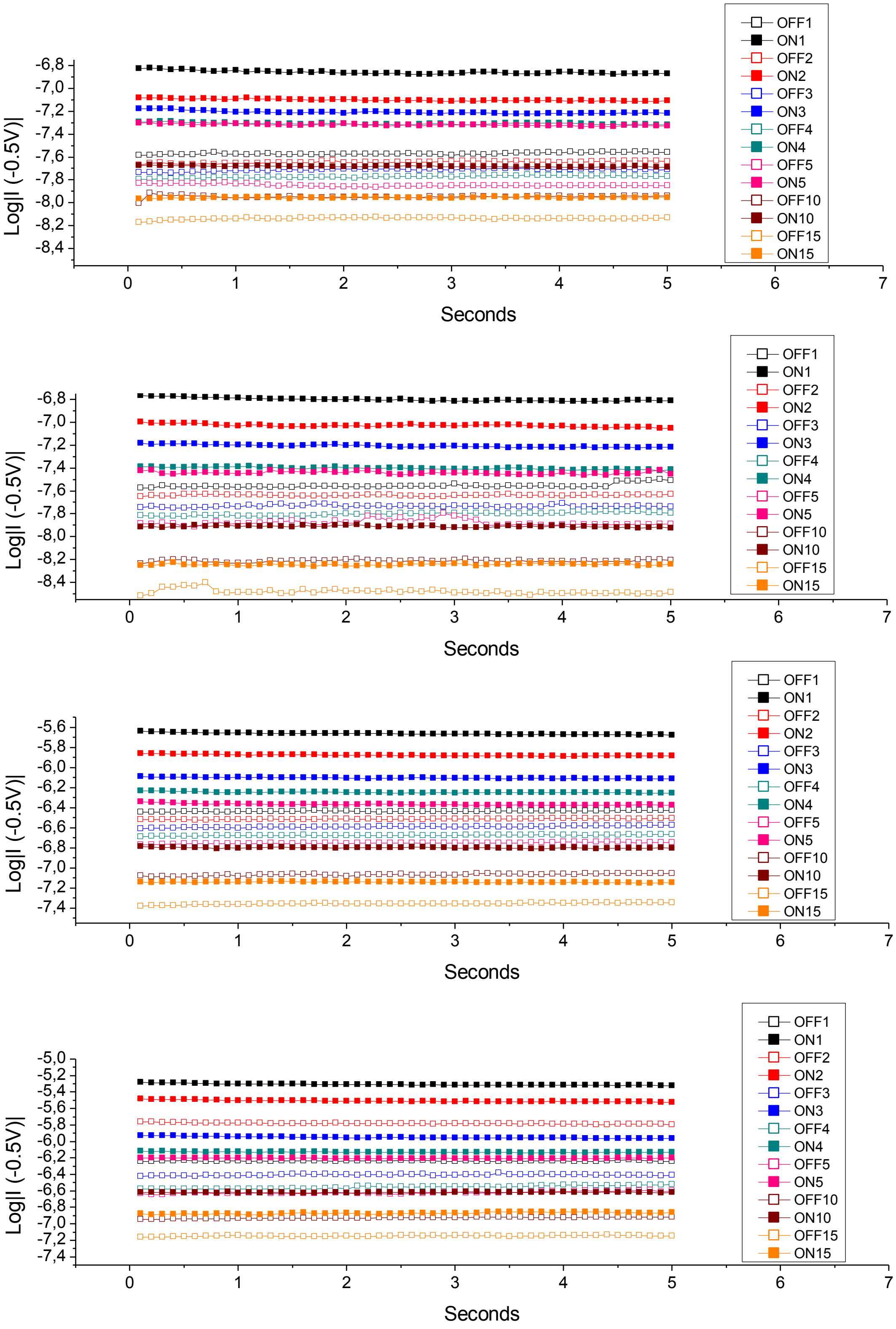

Figure S27: Examples of R_{ON} (solid symbols) and R_{OFF} (hollow symbols) for different Au-on-mica/**TCNAQ**//EGaIn for different cycles as reported in the legend.

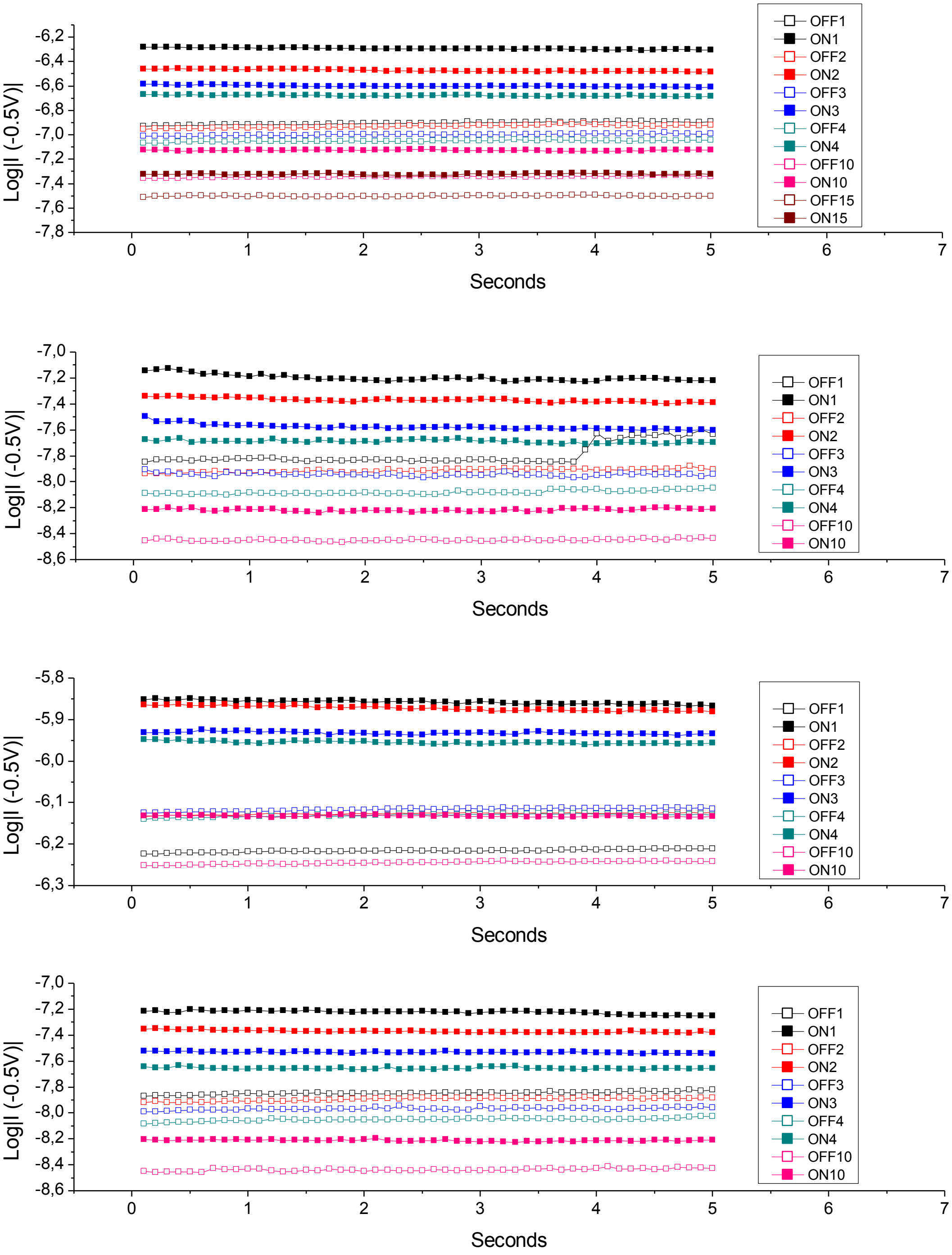

Figure S28: Examples of R_{ON} (solid symbols) and R_{OFF} (hollow symbols) for different Au^{TS}/**TCNAQ**//EGaIn for different cycles as reported in the legend.

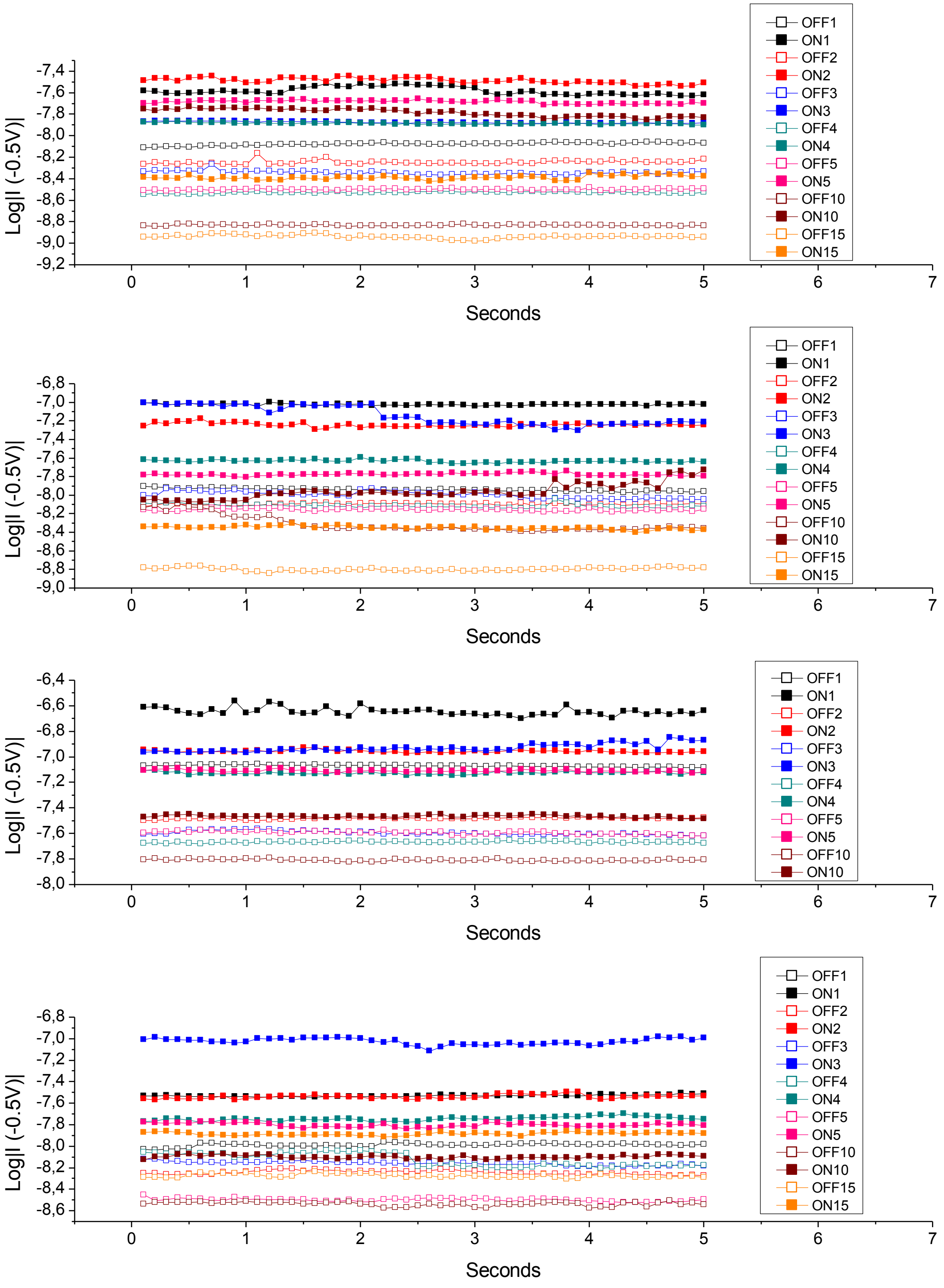

Figure S29: Examples of R_{ON} (solid symbols) and R_{OFF} (hollow symbols) for different Ag^{TS}/**TCNAQ**//EGaIn for different cycles as reported in the legend.

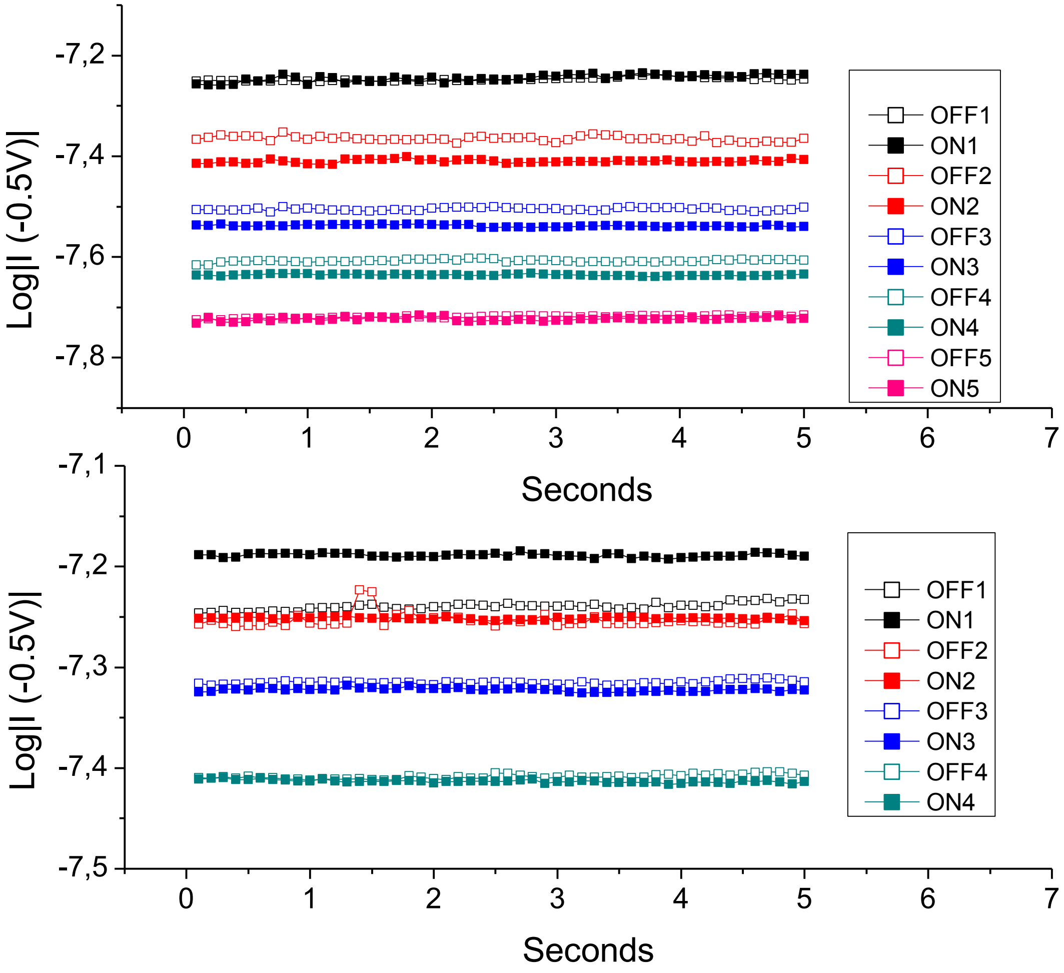

Figure S30: Examples of R_{ON} (solid symbols) and R_{OFF} (hollow symbols) for different Au^{TS}/**AC**//EGaIn for different cycles as reported in the legend.

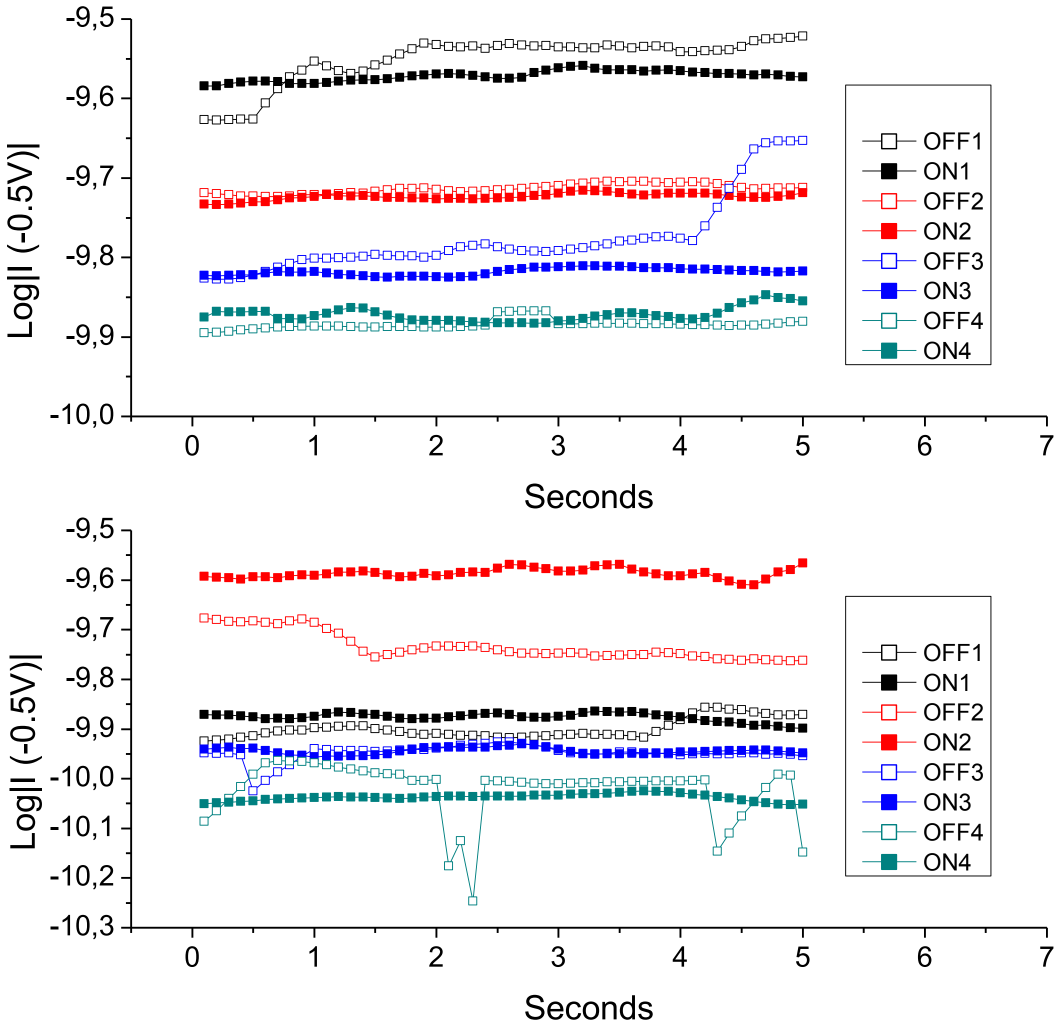

Figure S31: Examples of R_{ON} (solid symbols) and R_{OFF} (hollow symbols) for different Au^{TS}/**C16SH** //EGaIn for different cycles as reported in the legend.

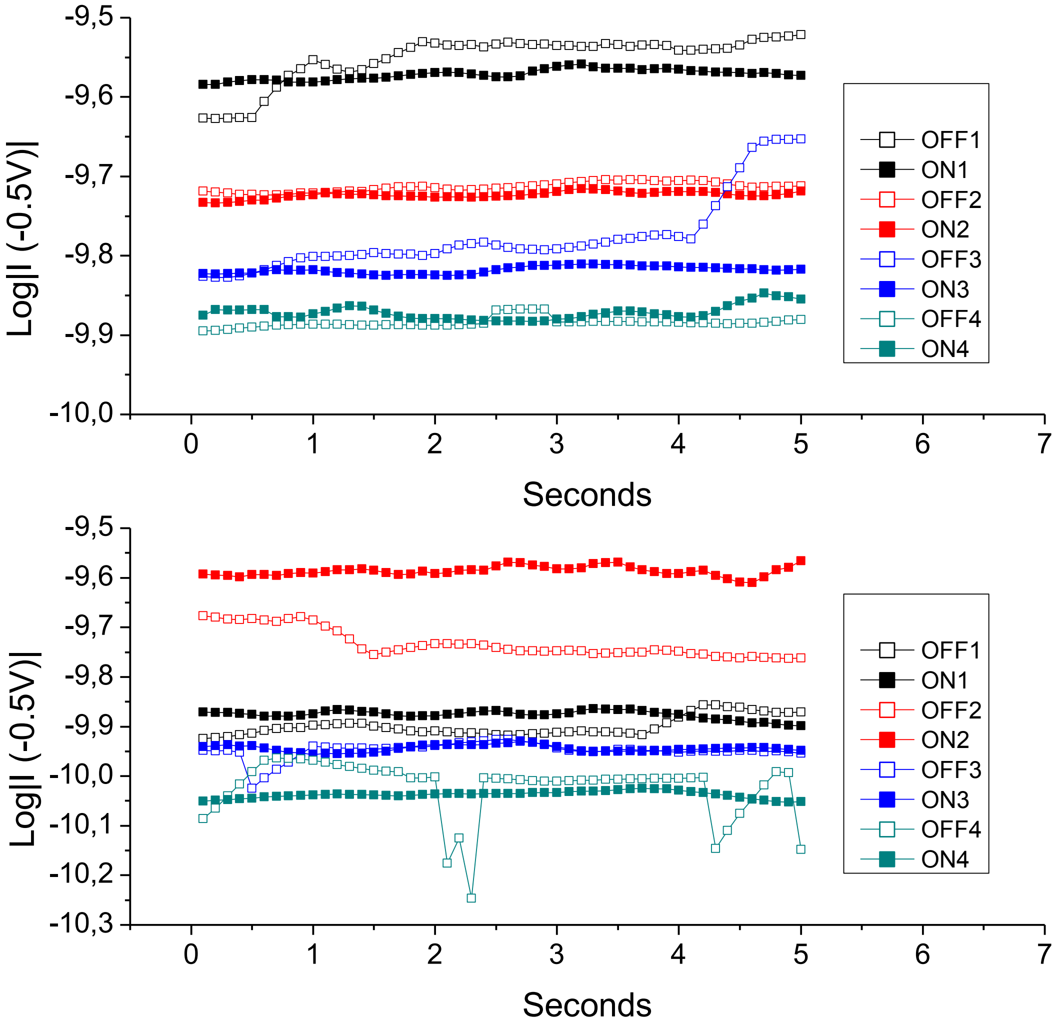

Figure S32: Examples of R_{ON} (solid symbols) and R_{OFF} (hollow symbols) for different Au-on-mica/**AQ**//EGaIn for different cycles as reported in the legend.

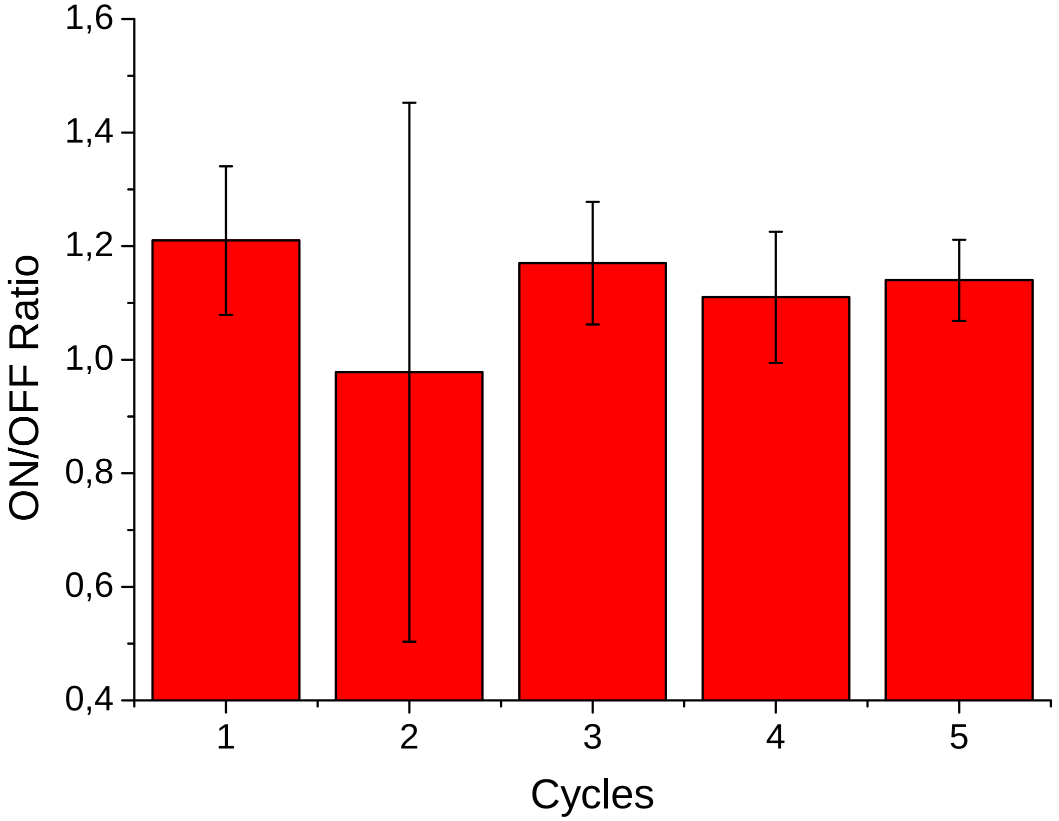

Figure S33: ON/OFF Ratio per n^{th} Write-Erase cycle, calculated as ratio of the current after and before the nth Write operation for SAMs of **TCNAQ** on Au^{TS} using $-1V$ for the Write operation. The error is calculated as confidence interval on 4 junctions with $\alpha = 0.05$.

4 Stability of TCNAQ junctions during WRER operations

Metal/SAM//EGaIn junctions are generally stable unless a bad contact is formed. This is also the case of the $J - V$ traces recorded in this work for the various system as can be observed in Figures S11-S23. On the other hand, the scenario is different when it comes to the junctions used for the WRER operations: as we can observe in Figures S27-S32, the values of current - both in the hihg and low conductance states - tend to decrease constantly with the number of WRER cycles. These data are summarized in Table S3. Although the rate can differ from one system to the other, a general decrease per cycles between 10% and 20% is found among all the systems, from **TCNAQ**, to other conjugated molecules, to the saturated **C16SH**. The cause of this phenomenon is unknown, but, bearing in mind its ubiquity among the different SAMs and substrates, a viable hypothesis involves a role of the EGaIn top electrode. It has been shown that, when applying a bias larger than ± 1 V for prolonged period of time, electrochemistry at the EGaIn oxide interface might not be negligible and play a role in the conductance of the junction:[S22] when performing the WRER cycles experiments, the EGaIn electrode is suddenly exposed to relatively large opposite biases which may affect the contact (*i.e.* wetting, coupling, shape, stress, etc.) between the top electrode and the SAM, thus decreasing the current. A similar decrease was also found when a bias of -1 V was used in the W operation (Table S4).
It is worth noting that applying a -2 V bias after 30 cycles restored an ON/OFF value close to the initial one (Figure S34). The chances of shorting the junction also increased.

We also studied for how long the **TCNAQ** SAM was capable to retain the ON and the OFF states while not being constantly addressed by R operations. To do so, the junctions was pulsed W (or E) and the current read (R) immediately after, after 5 minutes, and after 15 additional minutes. No other bias was applied to the junction neither was the EGaIn tip moved during the time between reads. This study was performed on 6 different junctions

Table S3: Variation in the OFF state current between one cycle and the following calculated as percentage.

Cycles	**TCNAQ** Au^{TS}	**TCNAQ** Au mica	**TCNAQ** Ag^{TS}	**AQ**	**AC**	**C16SH**
1st	-21%	+1%	-44%	-21%	-12%	-10%
2nd	-17%	-30%	-5%	-16%	-18%	-17%
3rd	-15%	-20%	-17%	-9%	-18%	-11%
4th	-14%	-18%	-9%	-14%	-16%	-11%
5th	-11%	-11%	-12%	-11%	-13%	-10%
10th	-7%					
15th	-10%					
20th	-6%					

Table S4: Variation in the OFF state current between one cycle and the following calculated as percentage in the case of Au^{TS}/**TCNAQ**/EGAIn junctions for which a bias of $-1V$ was used for Write operations.

n of cycle	variation in OFF current
1	-5%
2	-13%
3	-4%
4	-11%

(on 2 different samples) comprising SAMs of **TCNAQ** on Au^{TS}. The obtained results are summarized in Table S5. As we can see in the latter, the current measured during a Read of the ON state decreases to about 60% of its inital value after 20 minutes; the same is not observed for the OFF state, for which no trend in the Read current with time can be extrapolated. With an ON/OFF ratio of 3, the ON state current had to decrease to 30% of its initial value in order to be indistinguishable from the OFF state: in the case of our system it took 5 minutes to get to 69% and 20 minutes to get to 59%, meaning that it would take hours - if not days - for the ON state to go back to the OFF state.

Table S5: Incremental percentage variation of current during Read operations repeated after 5 and 20 minutes from the standard Read after a Write (ON) or Erase (OFF) operations.

	5'	+15'
ON	-31 ± 3 %	-14 ± 4 %
OFF	+4 ± 10 %	-7 ± 9 %

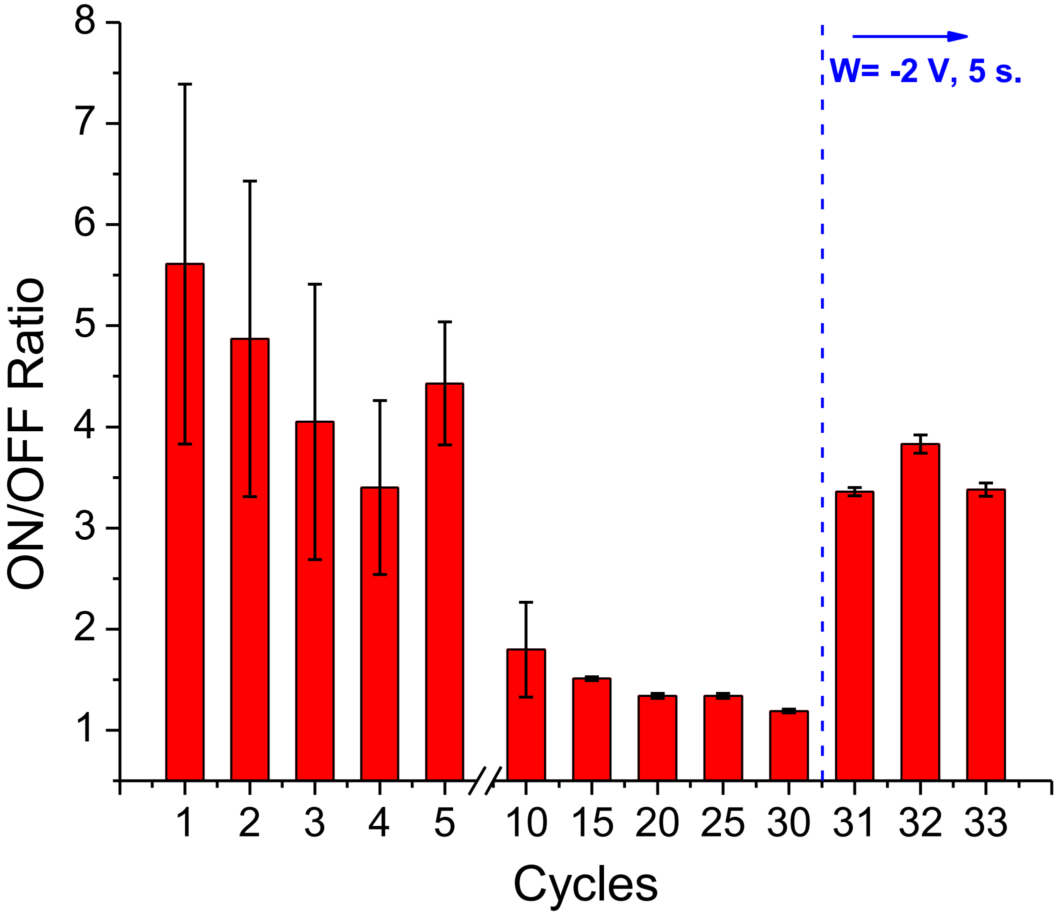

Figure S34: ON/OFF Ratio per n^{th} Write-Erase cycle, calculated as ratio of the current after and before the nth Write operation for SAMs of **TCNAQ** on Au^{TS}. The blue line highlights the 30th cycle after which a 5 s pulse of -2 V was use to perform the Write operation. The error is calculated as confidence interval with $\alpha = 0.05$.

5 Relations between the fraction of reduced molecules and the increase in conductance

In our earlier work we showed that only 1-2% of molecules in a SAM need be in a high-conductance state to modulate the conductance of a junction by orders of magnitude.[S23] In the current work, we have no way of estimating what fraction of molecules might be in the high/low conductance states in an assembled junction, under bias. We can however estimate the percentage of reduced nitrogen species that were present in the SAM during the XPS measurement, which is about 14%.

Perhaps coincidentally, that is the almost the exact percentage at which we predict the conductance to match the high-conductance of the hysteresis loop according to a model provided by Weiss *et al.* (Eq. 1) for estimating the total current flowing through a junction as a function of the fraction of molecules in the high-conductance state.[S24]

$$J = \chi * J_0 e^{-\beta d} + (1 - \chi) J_i \tag{1}$$

The model uses an estimate of the value of β (the tunneling decay coefficient) for the reduced form of **TCNAQ**, which we took from the experimental value of oligo(phenylene ethylene) wires in EGaIn junctions,[S3] the molecular length, injection current (J_0) and the experimental value, J_i, which is the current in the low-conductance state.

This estimate shows the height of the hysteresis loop is commensurate with the observed fraction of reduced nitrogen species that we assign to reduced **TCNAQ**. It does not, of course, explain why **TCNAQ** is as conductive as **AC**. We do expect **TCNAQ** to be more conductive than **AQ** because the latter is completely planar and, indeed, that is the case both in single-molecule and SAM junctions. But **AC** is still about 5x more conductive than **TCNAQ** in single-molecule junctions. One possibility is that **TCNAQ** adopts a more planar geometry in the SAM, which can have dramatic effects on conductance.[S4] Another is that the low bandgap of reduced **TCNAQ** makes it far more conductive[S25] than the

model above predicts (i.e., J_0 is much larger or β is much smaller) and that a small fraction of it persists even in the low-conductance state. As Fig. S35 shows, the influence of high-conductance molecules in a SAM is exponential and a very small amount of residual **TCNAQ** in the reduced form would be sufficient to shift the conductance to overlap with **AC**.

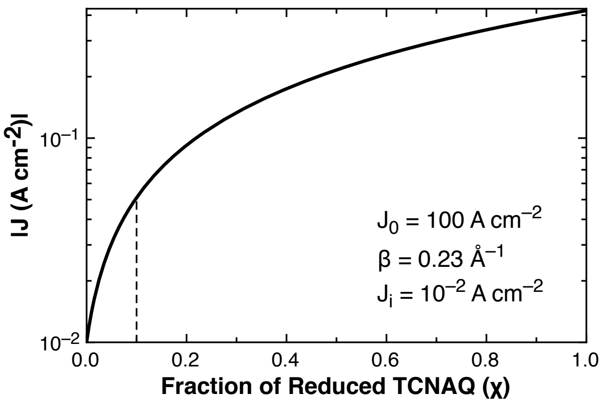

Figure S35: Effect on total current density J when increasing the fraction of high-conductance channels J_i according to Eq. 1, which was taken from Ref. S24. The dashed line corresponds to the fraction of high-conductance molecules required to give the experimentally-observed value of J ($\chi \approx 0.1$).

6 Stability of TCNAQ SAMs in different environments

In the main text, we proposed that the high conductance of molecular junctions comprising **TCNAQ** and their ability to show different conductance states, are arising from the contribution to the total current of reduced **TCNAQ** molecules in the assembly. These latter are generally stable in the SAM environment and are formed under thermodynamic conditions (as discussed in Section 2.1). Yet, by considering the previous WRER measurements (Section 3.2), it is clear how the ON/OFF Ratio and the OFF current are decreasing with the number of cycles. We argue here that this loss in performance has to do with oxidative damage due to the presence of oxygen in the measurement environment and not with the intrinsic mechanism discussed. Unfortunately, oxygen is mandatory to form the sharp EGaIn tips used to form the junctions and cannot be completely removed during the experiments. Yet, by performing the measurements in an atmosphere with a level of O_2 between 1-3% (as described in Section 3), we can slow down the kinetics of the oxidation process thus limiting the damage to the system.[S3]

When SAMs of **TCNAQ** are measured in ambient environment ($[O_2]= 21\%$), their properties change drastically: as we can see in Figure S36, the current density is two order of magnitude lower than what was measured in the controlled atmosphere and the hysteresis is minimal (if at all present). When WRER operations are applied to these junctions (Figure S37), no appreciable memory effect can be observed. These observation suggests that the level of oxygen found in air is enough to scavenge the reduced molecules and affect the properties of the SAM on the time-scale of the measurement.

If a sample is kept in the flowbox, at an oxygen concentration of 1-3%, the performances of **TCNAQ** SAMs on Au decrease more slowly. As we can see from Table S6, over a period of a week, both the ON/OFF ratio and the yield of working junctions tend to decrease with time, hinting to the limited stability to oxygen of the SAM more than the measurement conditions (we assume that these latter speed up the degradation process during WRER cycles).

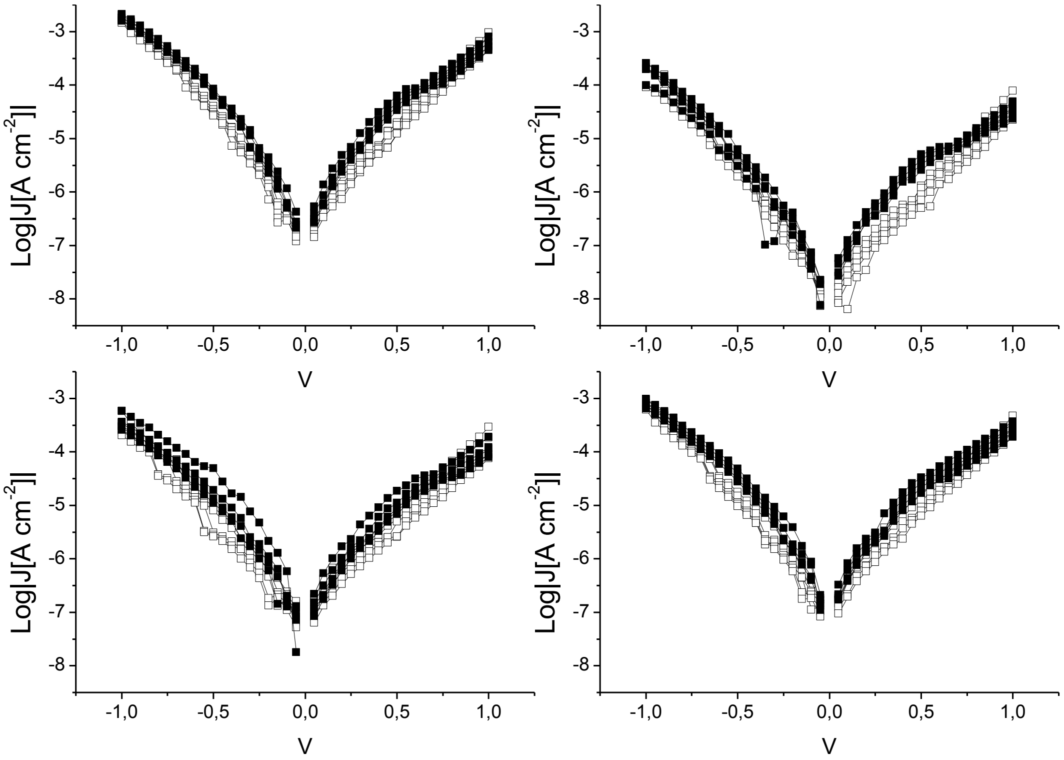

Figure S36: Examples of $Log|J|$ vs. V traces for Au^{TS}/**TCNAQ**//EGaIn junctions measured in ambient conditions ($[O_2]$= 21%; RH= 26%). Solid symbols represent forward traces from $-1V$ to $+1V$, hollow symbols reverse traces from $+1V$ to $-1V$.

Table S6: Summary of yield of working junctions and ON/OFF Ratios against aging time for **TCNAQ** SAMs stored in 1-3% oxygen.

	Yield (%)	ON/OFF Ratio per n^{th} cycle				
		1st	2nd	3rd	4th	5th
Day 1	100	7.3 ± 4.1	6.8 ± 2.6	6.0 ± 2.3	4.7 ± 1.0	4.5 ± 1.0
Day 2	75	4.8 ± 0.5	5.8 ± 0.26	5.1 ± 0.8	4.9 ± 0.45	4.5 ± 0.6
Day 3	50	3.6 ± 2.0	4.2 ± 0.5	5.0 ± 1.1	5.0 ± 0.7	4.3 ± 0.7
Day 6[1]	—	2.7	5.0	3.3	3.3	4.6

[1] We were able to measure only one working junction.

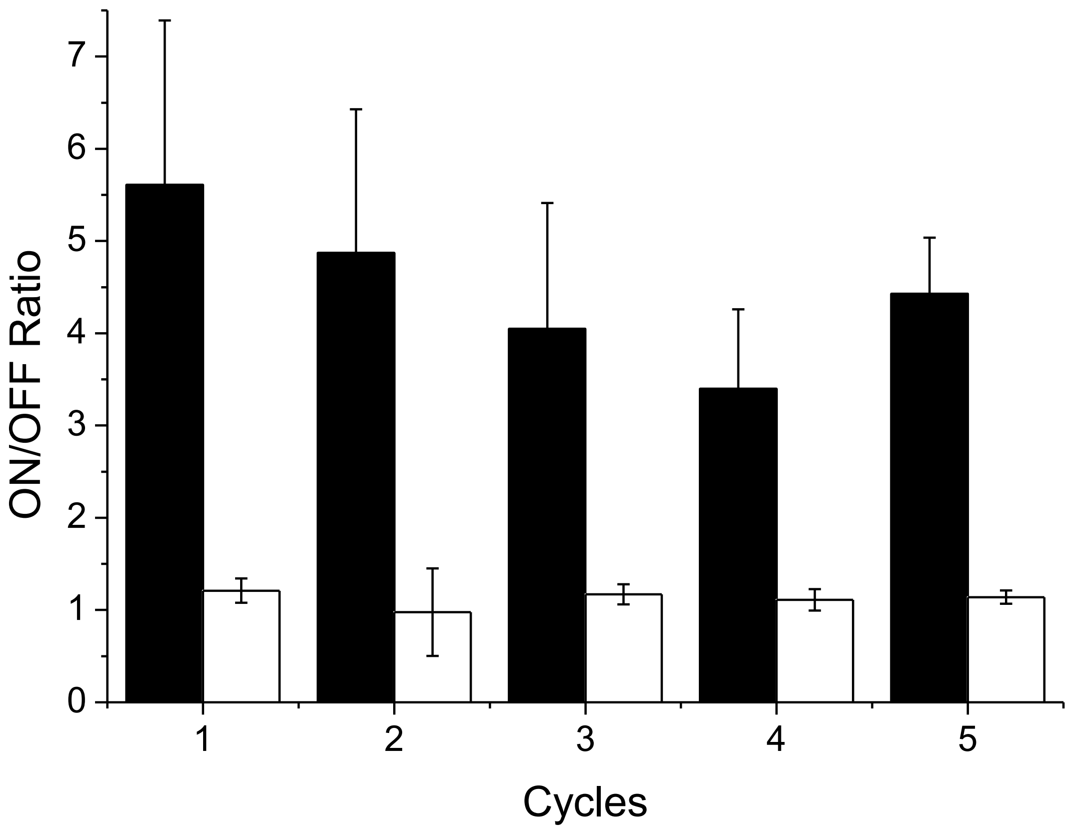

Figure S37: ON/OFF Ratio per n^{th} Write-Erase cycle, calculated as ratio of the current after and before the nth Write operation for SAMs of **TCNAQ** on Au^{TS} measured in the flowbox (black) and in ambient (white) conditions. The error for the latter is calculated as confidence interval on 4 junctions with $\alpha = 0.05$.

7 Properties of junctions comprising SAMs of TCNAQ on Pt^{TS}

We investigated also the electric properties of tunneling junctions comprising SAMs of **TC-NAQ** on Pt^{TS}. During $J - V$ sweeps characterization (measured as explained previously), we found that the current density of such system was similar to those of **TCNAQ** on the other metal substrates but no appreciable hysteresis loop was found (Figure S38). In order to see any effect, a larger bias window might be needed. The magnitude of hysteresis loop is bias dependent and a larger bias can indeed enhance small effects: in the case of **TCNAQ** SAMs on Pt^{TS} the hysteresis is noticeable if we extend the bias window until $-1.5V$ as we show in Figure S39. When performing WRER cycles (as described earlier), a small memory effect is present but its magnitude is drastically small when compared to the SAMs of the same compound on Au or Ag (Table S7). Seeing the limits of this system, we did not carry any further investigation.

Table S7: ON/OFF Ratio per n^{th} Write-Erase cycle, calculated as ratio of the current after and before the nth Write operation for SAMs of **TCNAQ** on Pt^{TS}. The error is calculated as confidence interval with $\alpha = 0.05$

n of cycle	ON/OFF ratio
1	1.77 ± 0.35
2	1.52 ± 0.12
3	1.55 ± 0.16
4	1.42 ± 0.07

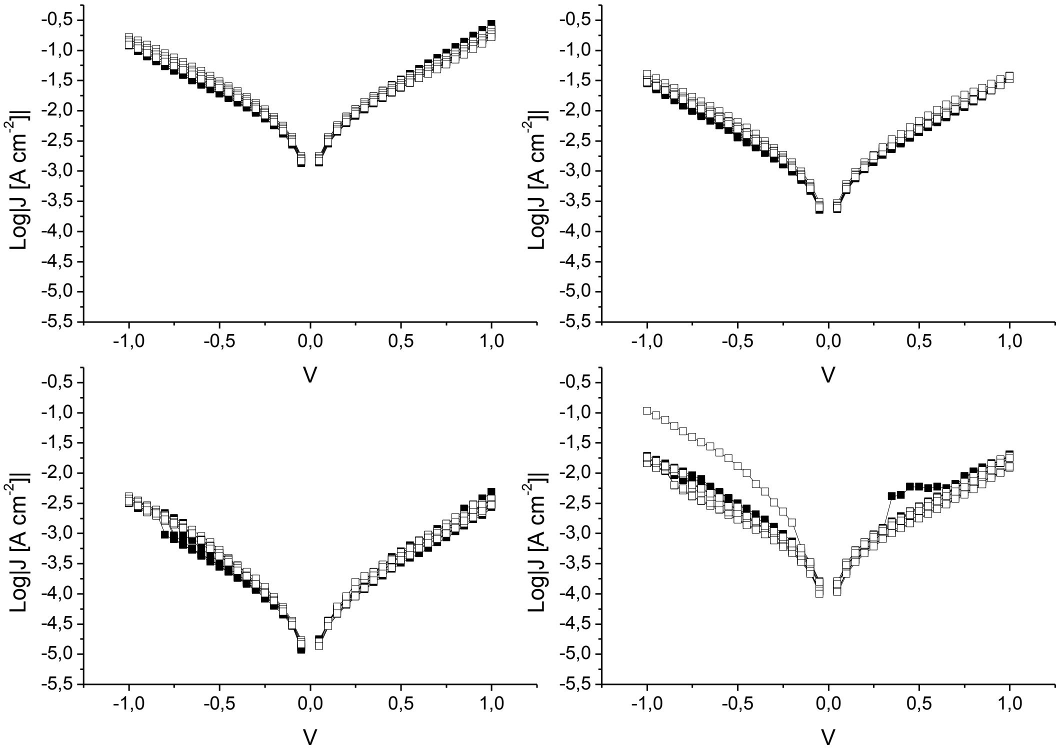

Figure S38: Examples of Log—J— vs. V traces for Pt^{TS}/**TCNAQ**//EGaIn junctions. Hollow symbols represent forward traces from $-1V$ to $+1V$, solid symbols reverse traces from $+1V$ to $-1V$.

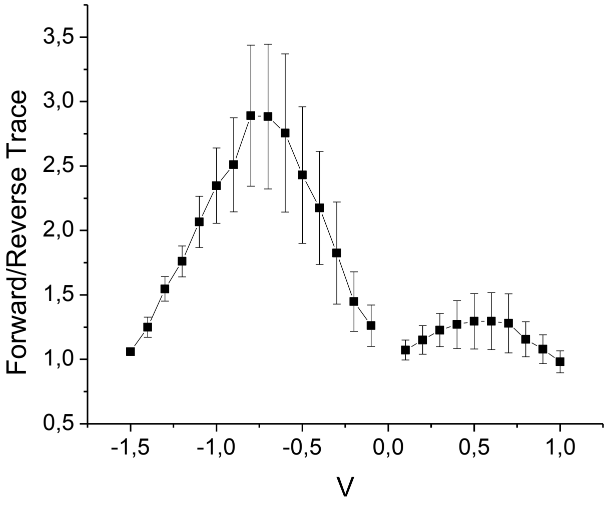

Figure S39: Plot of trace over retrace ratio for junctions comprising SAMs of **TCNAQ** on Pt^{TS}. please not that compared to Figure S38 here the bias window spans down to $-1.5V$. Error bars represent confidence intervals with $\alpha = 0.05$.

8 Single molecule conductance measurement

The STM break-junction experiments were performed with a Molecular imaging system using PicoScan software. We used a commercial Au-on-mica substrate (1x1 cm, Phasys, Switzerland) and a gold tip similar to that described in elsewhere.[S26] Prior to each experiment, the substrate was briefly annealed using a hydrogen flame. The STM tip was prepared by cutting a 0.25 mm gold wire (99.999%). The STM cell was cleaned with piranha solution (98% H_2SO_4:30% H_2O_2=3:1 v/v) and then sonicated three times in MilliQ water. We carried out the conductance measurements in a 0.1 mM mesitylene solution of the different compounds. Prior to the measurement, a small amount of NEt_3 was added into solution in order to cleave the thioacetate group.[S27]

Prior to the STM break junction measurement, the quality of the substrate was checked by scanning its surface in STM mode. Clear images and sharp atomic steps usually indicates a clean substrate and a sharp tip. After ensuring the tip and Au-on-mica substrate were in good conditions, the STM feedback loop was turned off. Then the tip was precisely driven by the piezo, leading to a repeatedly in and out of contact with the substrate. During such movements, the molecules may bridge both the tip and the substrate electrodes. Meanwhile, current versus tip travel time ($I(s)$) curves are recorded. Single molecular conductance is determined by the current plateau as well as the conductance histogram constructed from large number of individual events.

Figure S41 shows the results of STM break-junction studies for **AC**, **AQ** and **TCNAQ**. All the traces are recorded when the STM tip was pulled away from the substrate and under the bias voltage of $0.1V$. The $I(s)$ traces are presented in figure S41 a-c-e with arbitrary x-axis offsets. The dramatic conductance drop associated with the tip stretching suggests a decrease in the number of molecules involved in the junctions. The last conductance plateau is usually taken as the single molecular conductance. The higher conductance step in figure S41a suggests that double molecules May contribute to the conductance of the junctions. To determine the conductance of the single molecules, 5000 current-distance traces are acquired

for statistical studies. The conductance histogram in Figure S41 b-d-f was constructed using 710, 775 and 350 individual traces selected from the acquired traces, respectively. They show a conductance maximum at 4.7×10^{-5}, 1.9×10^{-6} and 9.0×10^{-6} corresponding to **AC**, **AQ** and **TCNAQ**. It gives an conductance order: **AQ**<**TCNAQ**<**AC**. In saying this we assume **TCNAQ** to be contacted through the two terminal S, but we cannot exclude the possibility that this molecule binds to the electrodes via one (or even both) cyano groups. From the 2D conductance plots (Figure S40) we can observe how the plateaus of **TCNAQ** compared to those of **AC**are about 0.1 nm to 0.2 nm shorter; however, compared to **AC**, **TCNAQ** has a bent structure that shortens the S-S distance. According to DFT calculations, the differences in the S-S distances between **AC** and **TCNAQ** is 0.2 nm, while the distance between the cyano groups is 0.7 nm shorter than the S-S distance of **AC**.

This order of single-molecule conductance is strikingly different from what was observed in the case of EGaIn (for which **TCNAQ** was found as conductive as **AC**). This reflects the role of the SAM in determining the electrical characteristics of the junctions: in the case of **TCNAQ**, the strong dipoles present in the monolayer stabilize the highly conductive form of the molecule which translates to high conductance; this stabilization is lost in single molecule measurements, where **TCNAQ** behave like a cross conjugated system, more similar to **AQ**.

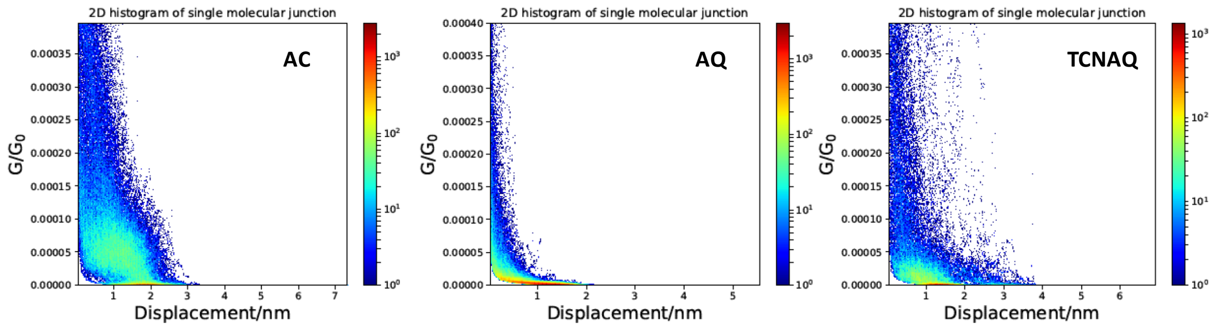

Figure S40: 2D-conductance histograms of **AC**, **AQ**, and **TCNAQ** obtained from the previous traces.

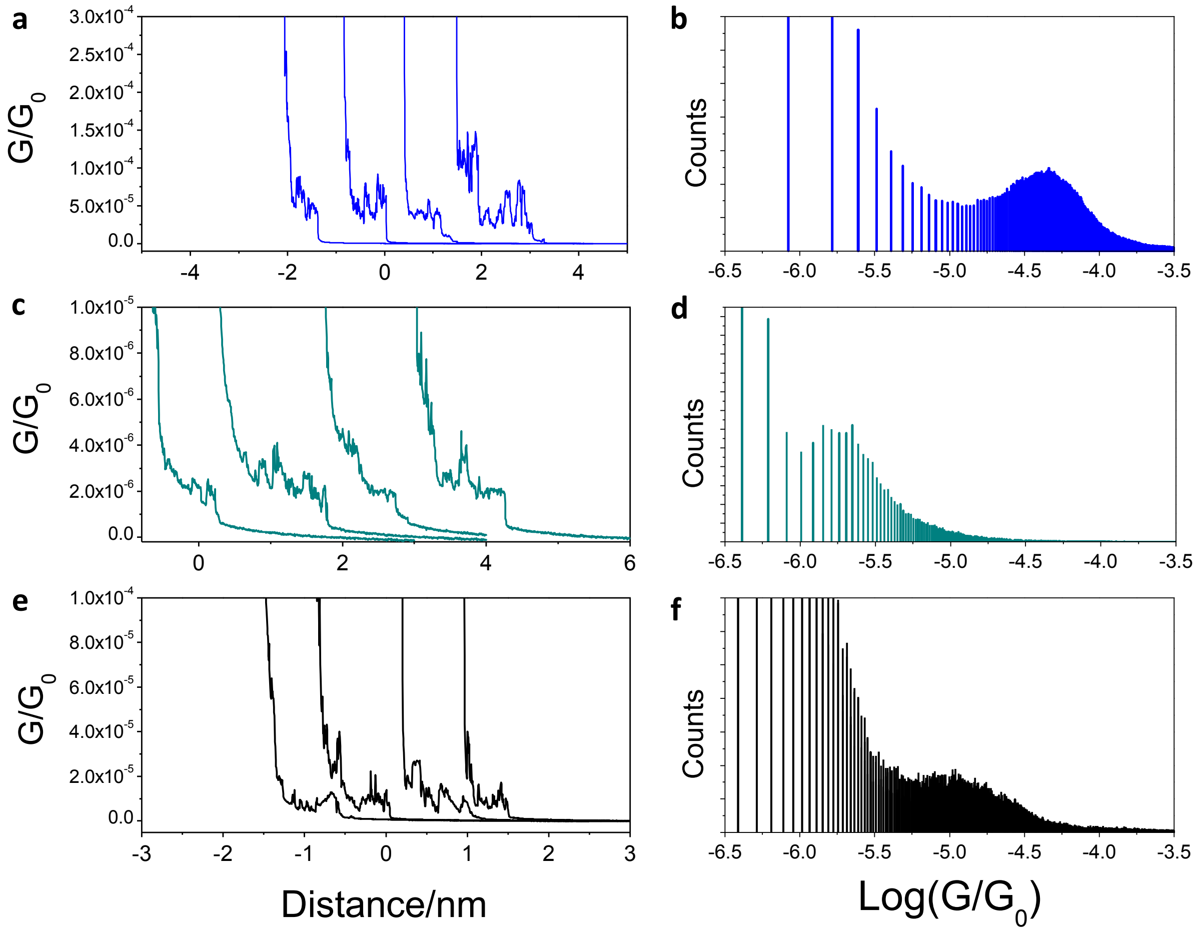

Figure S41: Typical conductance traces of the **AC** (a) **AQ** (c) and **TCNAQ** (e) using STM-BJ plotted against an arbitrary x-axis offset. Conductance histograms of **AC** (b) **AQ** (d) and **TCNAQ** (f) obtained from the previous traces. The banding at low conductance is due to compliance limits of the current amplifier.

9 Calculations

The calculations were performed using the *Orca 4* software package.[S28,S29] The molecules were first minimized to find the gas-phase geometry and then single-point energy calculations were performed on both the gas-phase molecules and the molecules terminating with Sulphur atoms (*i.e.*, no terminal H atoms). This was also repeated for the reduced **TCNAQ** molecule. The structures are shown in Fig. S42 and S43. Transmission curves were generated using the *Artaios-030417* software package on the molecules terminating with S atoms.[S30]

9.0.1 Geometry Optimization

We optimized the neutral and the reduced forms of **TCNAQ** molecules with the hydrogens removed from the terminal thiol groups using the *Orca* software package[S28,S29] (Figure S42 and S43). We minimized the geometries using BP/def2-SVP (BP functional, Ahlrichs split-valence basis set) with tight SCF and geometry convergence criteria, which can be accessed with the '! Acc-Opt' command.[S31] Note that the removal of the hydrogen atoms broadens the terminal sulfurs compared to thiols.

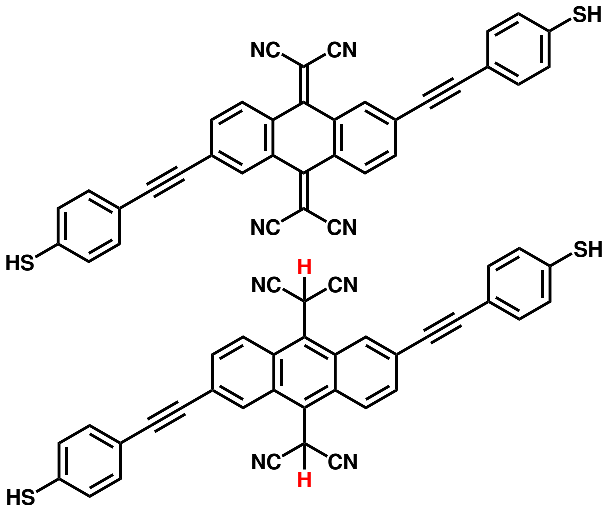

Figure S42: Structures of neutral **TCNAQ** (top) and the neutral, formally reduced form, **TCNAQ**H_2 (bottom).

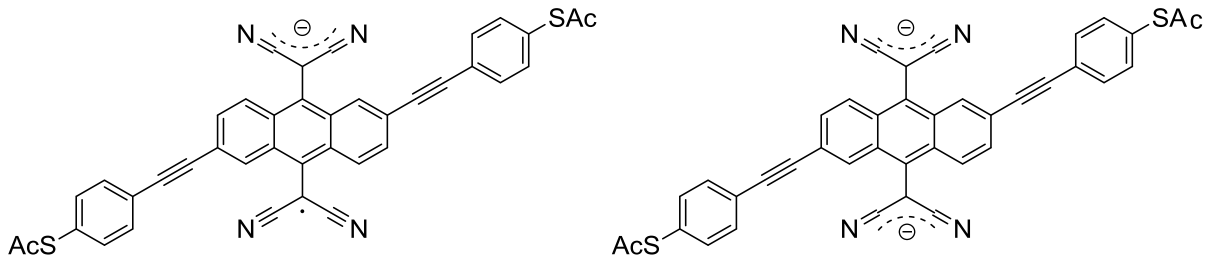

Figure S43: Structures of mono- and di-anion reduced **TCNAQ**.

9.0.2 Single Point Energy Calculations

We computed the single-point energies of the optimized geometries using B3LYP/LANL2DZ (B3LYP functional, Los Alamos double-valence basis set). We used the Gaussian-style B3LYP functional and tight SCF convergence criteria, which can be accessed with the '! DFT B3LYP/G LANL2DZ TIGHTSCF' command. We chose this functional/basis-set combination because it is recommended by the authors of the software used for transport calculations.[S32]

9.1 Transport Properties

We followed the same procedures as described by Zhang *et al.*.[S33] For computing the electron transmission probability plots as function of the energy of electron, we first ran single point energy calculations on molecules without hydrogen atoms on terminal sulphur groups as described in Section 9.0.2. The Hamiltonian (Fock) and overlap matrices were generated from the output of these energy calculations with the commands 'Print[P_Iter_F] 1', 'Print[P_Overlap] 1', 'Print[P_Mos] 1', 'Print[P_InputFile] 1' in the '%output' section. In the case of the radical anions, Hamiltonian matrices were generated for the α and β spin states. The Hamiltonian and overlap matrices were then used as inputs for *Artaios-030417* to generate the transmission curves using the non-equilibrium Green's function[S30,S32] This method separates the finite cluster system into a bulk calculation / approximation for the electrodes and a central subsystem that may or may not include some of the atoms from the

electrodes. We omitted the electrodes and computed the transmission between the terminal sulfur atoms. We chose E_f value of -4.3 eV [S34,S35] to scale the $E - E_\mathrm{f}$ energy axis shown in the Main Text. This value is both an approximation of the work function of Ga-In and the value of Au modified with a thiol-SAM. [S36]

We omitted the electrodes because we could not capture the collective effects of the SAM, particularly in a mixed-state of neutral and reduced molecules. Thus, the energies of the orbitals for the two anionic forms, $\mathbf{TCNAQ}^{\bullet-}$ and $\mathbf{TCNAQ}^{2-}$ were pushed very close to vacuum due to the absence of electrodes, counterions, solvation and the near-by molecules that would be present in a SAM. Figure S44 shows the physically unrealistic result of these calculations, which places unoccupied orbitals below E_f in some calculations and occupied above E_f in others. The doubly-reduced species, $\mathbf{TCNAQ}^{2-}$ is particularly illustrative, placing both frontier orbitals just below 0 eV.

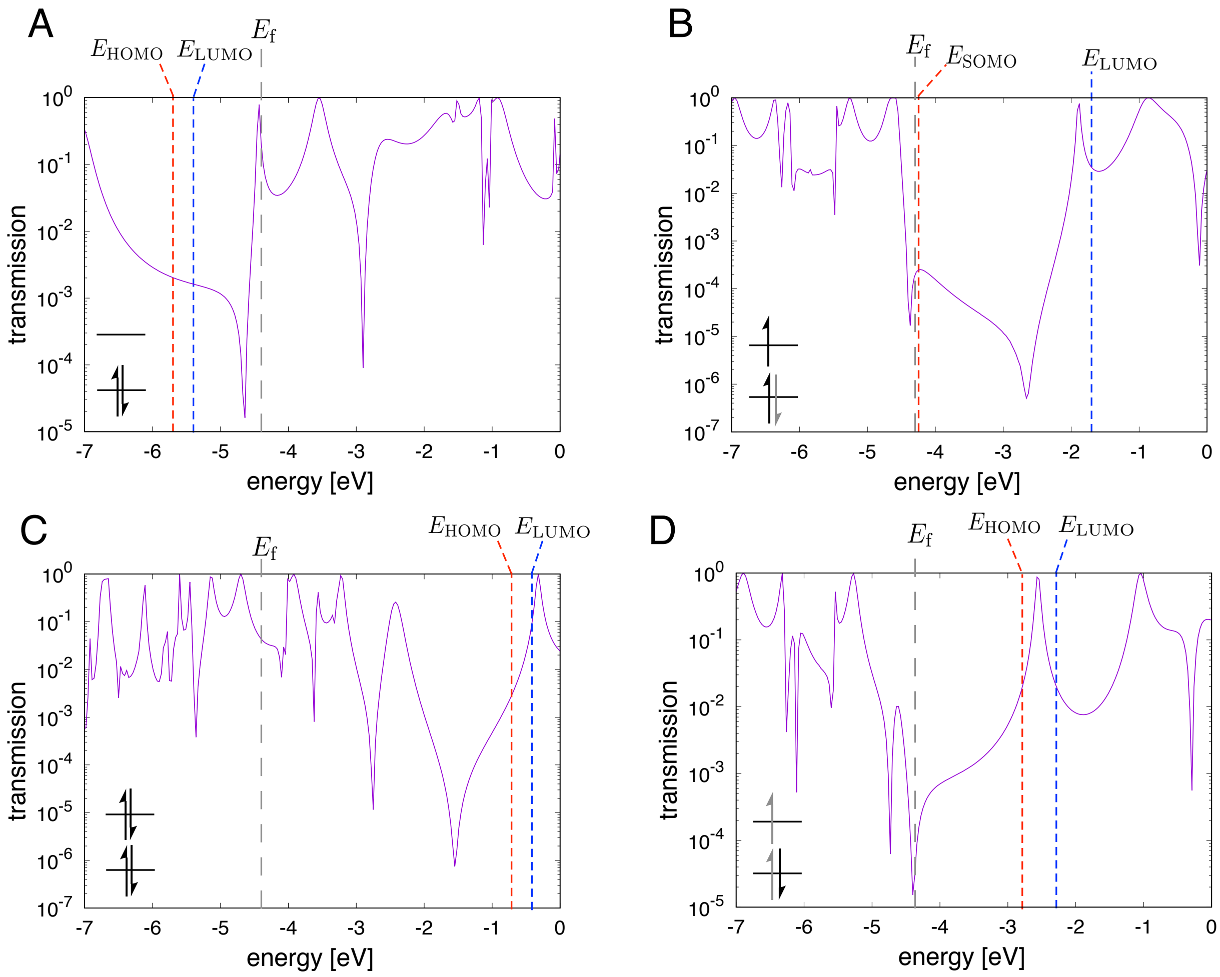

Figure S44: Transmission plots from uncorrected DFT calculations. A) Neutral **TCNAQ**. B) The α spin channel of **TCNAQ**$^{\bullet -}$. C) **TCNAQ**$^{2-}$. D) The β spin channel of **TCNAQ**$^{\bullet -}$. The gray, vertical dashed line corresponds to $E_{\rm f} = -4.3\,{\rm eV}$. The electron configuration of the frontier orbitals relative to neutral **TCNAQ** is shown in the insets. The uncorrected orbital energies under-estimate the HOMO/LUMO gap of **TCNAQ** and, due to the lack of counterions and solvation, shift the orbitals of the reduced species close to vacuum.

To try to compensate for the unknowables and assumptions in the DFT calculations, we re-plotted the transmission curves using experimental values taken from CV (Fig. S4) and UV-Vis (Fig. S3) data, which provide the energies of the LUMO and SOMO of **TCNAQ** and **TCNAQ**$^{\bullet -}$ (from CV) and the frontier orbital gap of **TCNAQ** (from UV-Vis), from which the HOMO can be estimated. Note that this is a linear shift that does not correct the under-estimated $E_{\rm g}$, which would broaden the dip near $E_{\rm f}$, but would not affect the

interpretation of the results. Figure S45 shows the results of these calculations, which place the LUMO and SOMO orbtials very close in energy to $E_{\mathrm{f}} = -4.3\,\mathrm{eV}$,[S34–S36] which is both the estimated work function of EGaIn and of Au accounting for the vacuum-level shift imposed by thiol-SAMs. The loss of the suppressed transmission and sharp dip near $E_{\mathrm{f}} = -4.3\,\mathrm{eV}$ for **TCNAQ** is evident in both the α and β spin channels of **TCNAQ**$^{\bullet -}$. We interpret this difference as the loss of QI when the cross-conjugated core of **TCNAQ** is replaced by the linearly-conjugated core that is expected to be (by far) the dominant resonance structure of **TCNAQ**$^{\bullet -}$, however, the introduction of unpaired spins strongly affects the features of the transmission plots. Thus, we also plotted the formally reduced, but neutral hydroquinoid form, **TCNAQ**H_2, in which the cross-conjugation is deliberately removed by the addition of H_2. The comparison between **TCNAQ** and **TCNAQ**H_2 clearly highlights the role of conjugation patterns. Taken together, the data in Fig. S45 support our proposed mechanism, which localizes spin and charge on the central carbons of the malononitrile substituents, favoring the rearomatization of the anthracene core.

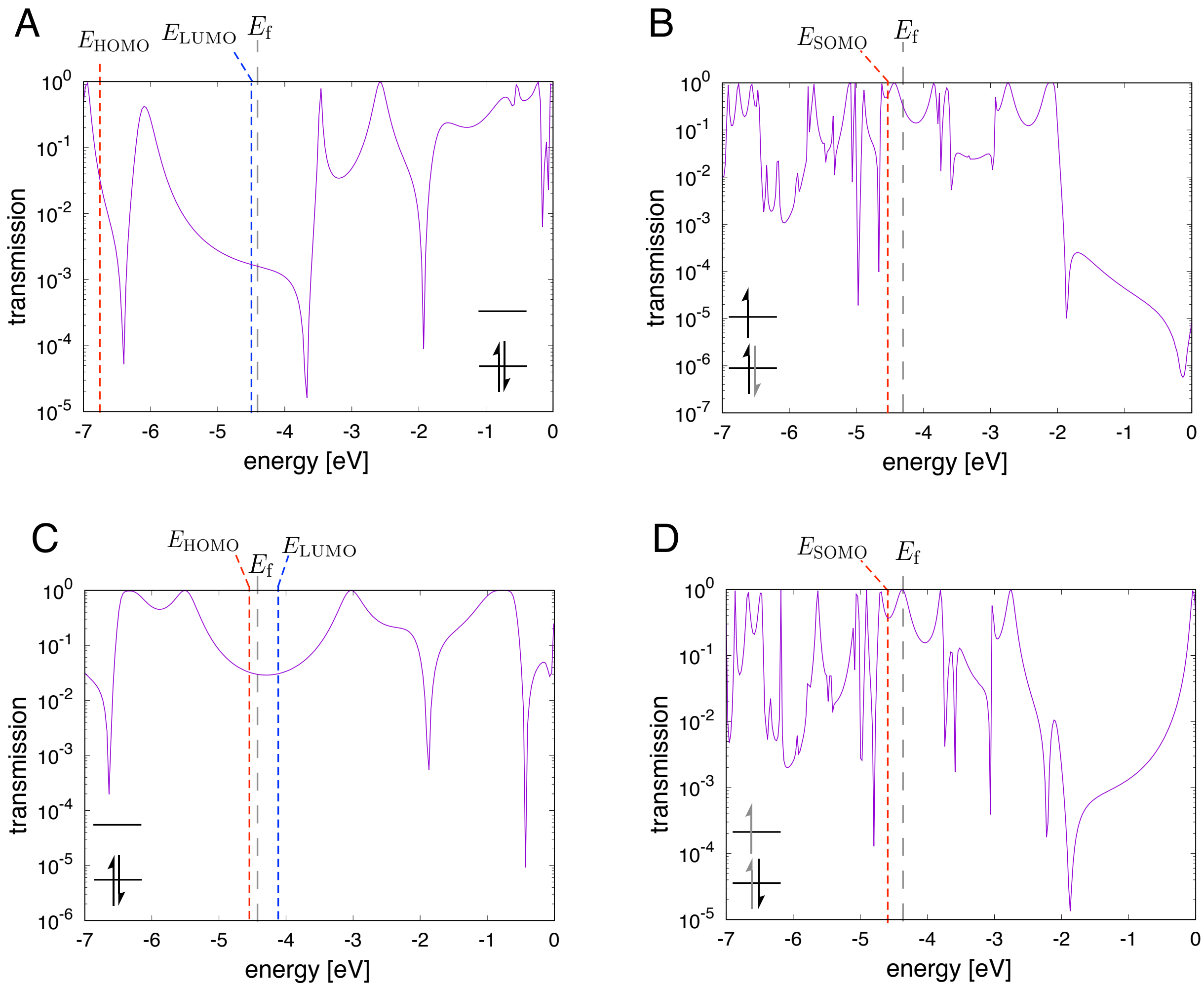

Figure S45: Transmission plots from DFT calculations corrected using experimental values. The energies E_{LUMO} and E_{SOMO} are shown using vertical dashed lines obtained using the experimental values from CV (Fig. S4). E_{HOMO} for **TCNAQ** was estimated by subtracting the onset of absorption (525 nm from E_{LUMO} from CV, Fig. S3) and taken from DFT for **TCNAQ**H_2, hence the large difference in E_{g} between the two. A) Neutral **TCNAQ**. B) The α spin channel of **TCNAQ**$^{\bullet-}$. C) Neutral **TCNAQ**H_2, the formally-reduced hydroquinoid form of **TCNAQ** in which the cross-conjugation has been deliberately broken by the addition of H_2. D) The β spin channel of **TCNAQ**$^{\bullet-}$. The gray, vertical dashed line corresponds to $E_{\mathrm{f}} = -4.3\,\mathrm{eV}$. The electron configuration of the frontier orbitals relative to neutral **TCNAQ** is shown in the insets. The corrected values place the energies of E_{LUMO} and E_{SOMO} very close to E_{f}, in agreement with our proposed mechanism of switching. The sharp dip and depressed transmission near E_{f} for **TCNAQ** are absent for both spin channels of **TCNAQ**$^{\bullet-}$, but the influence of the conjugation patterns is more clearly resolved in the comparison between the two neutral species, **TCNAQ** and **TCNAQ**H_2.

9.1.1 XYZ Coordinates

All values are in units of Å.

Parent TCNAQ

S 0.03083379 0.18278209 -0.09458313

C -0.51863676 0.28723312 1.58812574

H -0.25876895 2.45625372 1.60700365

H -0.88897268 -1.84459304 1.90117151

C -0.56035042 1.57021032 2.18681287

C -0.91212770 -0.83903488 2.34887853

C -0.98334152 1.72145946 3.50799378

C -1.33471694 -0.68755008 3.67128418

H -1.01295064 2.72299508 3.96175132

H -1.64173934 -1.57010871 4.25157228

C -1.38006309 0.59576680 4.28239404

C -1.81303502 0.74595831 5.62759193

C -2.19383590 0.86730398 6.79594644

N -4.97650421 -2.75461492 8.08841642

C -2.64168170 1.00103025 8.13632982

H -2.55600429 3.18258014 8.15884386

H -2.78810531 -1.13511700 8.45612354

C -2.79445760 2.28012474 8.73952126

C -2.93541428 -0.14948653 8.91492041

C -4.62102887 -2.55281992 9.19114932

C -3.22294695 2.39438926 10.06077805

C -3.36748776 -0.04495861 10.24834936

H -3.29963320 3.39529552 10.50537011

C -4.22755614 -2.39609152 10.56316665

C -3.51377210 1.25148727 10.84472139

C -3.64962432 -1.23339824 11.08258463

N -5.76263936 4.28518389 11.33409718

C -4.58440234 -3.53235960 11.36565936

C -5.27254489 3.40578929 11.94179195

N -4.90981927 -4.49238966 11.96172833

C -3.91584771 1.33250192 12.25586310

C -3.30847181 -1.07974107 12.50367043

C -4.72270123 2.35891077 12.75671274

H -2.69398006 -3.14282224 12.85465621

C -3.43088091 0.21908176 13.10030895

C -2.82364328 -2.14697554 13.29824815

C -5.17630688 2.43141308 14.11716271

C -3.06204641 0.40207485 14.44420126

C -2.46043269 -1.95530264 14.63000168

H -3.11995312 1.39660524 14.90379894

H -2.06787247 -2.79621041 15.21927707

C -2.57247569 -0.67202873 15.23334954

N -5.59426354 2.55561786 15.20937772

C -2.19266644 -0.45836240 16.58430982

C -1.87108853 -0.27143557 17.76180188

C -1.50617908 -0.04986368 19.11727655

H -2.11913595 2.04461437 19.13117367

H -0.80500239 -2.08784878 19.45746821

C -1.68774382 1.22457420 19.72377066

C -0.95203513 -1.09460172 19.90672028

C -1.33089465 1.44245611 21.05504162

C -0.59435565 -0.87662860 21.23902732

H -1.48275159 2.43667310 21.50296506

H -0.16751704 -1.70420119 21.82651969

C -0.77841323 0.39487440 21.83190568

S -0.35183784 0.75606894 23.51469804

TCNAQ$^{\bullet-}$

S -0.12479021 0.14178329 -0.17778839

C -0.65109046 0.26714105 1.52143272

H -0.37616834 2.43297646 1.52413290

H -1.03170752 -1.85842575 1.85438165

C -0.67411551 1.55114341 2.11310202

C -1.04026458 -0.84904497 2.29544563

C -1.07420674 1.71272183 3.44264051

C -1.44181265 -0.68647805 3.62498303

H -1.08808803 2.71736366 3.89083158

H -1.74756330 -1.56294946 4.21538021

C -1.46852551 0.59856087 4.23496647

C -1.87771677 0.75681254 5.58808742

C -2.23934096 0.87383091 6.76321228

N -4.64463099 -2.93379054 8.04156667

C -2.66490638 1.00660844 8.11439289

H -2.60623017 3.19661927 8.12494109

H -2.76098738 -1.11923725 8.44415341

C -2.82031506 2.29353946 8.71492738

C -2.91884502 -0.13558737 8.90235725

C -4.32956063 -2.68661048 9.15186682

C -3.21030488 2.40128553 10.04150258

C -3.31982473 -0.04979301 10.25699160

H -3.28292036 3.40231554 10.48470134

C -3.98373617 -2.48592858 10.52399455

C -3.46917242 1.26036382 10.85544243

C -3.53306192 -1.24595038 11.07360994

N -5.49399051 4.49506585 11.42973407

C -4.28075084 -3.64969720 11.29879998

C -5.03598973 3.57529799 12.01045926

N -4.55736010 -4.64560249 11.86864459

C -3.81868220 1.37522425 12.26416876

C -3.25507379 -1.06666069 12.49134884

C -4.52977877 2.49435332 12.79678330

H -2.65260371 -3.12840831 12.87459548

C -3.39021933 0.24497939 13.09010050

C -2.79539314 -2.13421949 13.31615738

C -4.95197689 2.61268941 14.15709973

C -3.05247684 0.40696705 14.45498355

C -2.47283103 -1.95326789 14.65268303

H -3.10906877 1.40121615 14.91412320

H -2.09809712 -2.79619874 15.25146429

C -2.59554855 -0.66263119 15.25321739

N -5.34580579 2.78348213 15.25651145

C -2.24034054 -0.45313287 16.61490374

C -1.93765716 -0.27831316 17.79952810

C -1.59590320 -0.06486559 19.16370284

H -2.22397948 2.02241455 19.18898121

H -0.88363667 -2.09647610 19.50545564

C -1.79428820 1.20171998 19.78220638

C -1.04536392 -1.10735773 19.95929983

C -1.45840626 1.41328106 21.12216593

C -0.70882120 -0.89633815 21.30031676

H -1.62578418 2.40402478 21.57333243

H -0.28398905 -1.72584860 21.88779275

C -0.91024820 0.36698046 21.89973864

S -0.50566052 0.71628293 23.60104268

TCNAQ^{-2}

S -0.12479021 0.14178329 -0.17778839

C -0.65109046 0.26714105 1.52143272

H -0.37616834 2.43297646 1.52413290

H -1.03170752 -1.85842575 1.85438165

C -0.67411551 1.55114341 2.11310202

C -1.04026458 -0.84904497 2.29544563

C -1.07420674 1.71272183 3.44264051

C -1.44181265 -0.68647805 3.62498303

H -1.08808803 2.71736366 3.89083158

H -1.74756330 -1.56294946 4.21538021

C -1.46852551 0.59856087 4.23496647

C -1.87771677 0.75681254 5.58808742

C -2.23934096 0.87383091 6.76321228

N -4.64463099 -2.93379054 8.04156667

C -2.66490638 1.00660844 8.11439289

H -2.60623017 3.19661927 8.12494109

H -2.76098738 -1.11923725 8.44415341

C -2.82031506 2.29353946 8.71492738

C -2.91884502 -0.13558737 8.90235725

C -4.32956063 -2.68661048 9.15186682

C -3.21030488 2.40128553 10.04150258

C -3.31982473 -0.04979301 10.25699160

H -3.28292036 3.40231554 10.48470134

C -3.98373617 -2.48592858 10.52399455

C -3.46917242 1.26036382 10.85544243

C -3.53306192 -1.24595038 11.07360994

N -5.49399051 4.49506585 11.42973407

C -4.28075084 -3.64969720 11.29879998

C -5.03598973 3.57529799 12.01045926

N -4.55736010 -4.64560249 11.86864459

C -3.81868220 1.37522425 12.26416876

C -3.25507379 -1.06666069 12.49134884

C -4.52977877 2.49435332 12.79678330

H -2.65260371 -3.12840831 12.87459548

C -3.39021933 0.24497939 13.09010050

C -2.79539314 -2.13421949 13.31615738

C -4.95197689 2.61268941 14.15709973

C -3.05247684 0.40696705 14.45498355

C -2.47283103 -1.95326789 14.65268303

H -3.10906877 1.40121615 14.91412320

H -2.09809712 -2.79619874 15.25146429

C -2.59554855 -0.66263119 15.25321739

N -5.34580579 2.78348213 15.25651145

C -2.24034054 -0.45313287 16.61490374

C -1.93765716 -0.27831316 17.79952810

C -1.59590320 -0.06486559 19.16370284

H -2.22397948 2.02241455 19.18898121

H -0.88363667 -2.09647610 19.50545564

C -1.79428820 1.20171998 19.78220638

C -1.04536392 -1.10735773 19.95929983

C -1.45840626 1.41328106 21.12216593

C -0.70882120 -0.89633815 21.30031676

H -1.62578418 2.40402478 21.57333243

H -0.28398905 -1.72584860 21.88779275

C -0.91024820 0.36698046 21.89973864

S -0.50566052 0.71628293 23.60104268

$TCNAQH_2$

S -2.27814005222275 0.07302656862768 -0.66369565244986

C -2.31925488719353 0.25055443819606 1.02523015529954

H -2.45661880871192 2.43021007204687 0.98128351149409

H -2.19226903539896 -1.89005095393003 1.44247980056551

C -2.41219407249742 1.55245745241814 1.64330215836135

C -2.26259024195108 -0.89387942634888 1.90430093667358

C -2.44474672954675 1.69963088071626 3.01763820651420

C -2.29488867542216 -0.75172976420762 3.27918594333703

H -2.51558610175702 2.69936129322442 3.47149346459421

H -2.25051652947877 -1.63507091427368 3.93352179724198

C -2.38693074815064 0.55076674429416 3.87893830229743

C -2.41941743053858 0.69878852329224 5.27239147709855

C -2.45144167361624 0.83074292953111 6.50802184160642

N -4.43532147977399 -4.03416554723094 10.92976125709351

C -2.48936071035473 0.99707974666955 7.90346368534799

H -2.62661911979211 3.18867614026023 7.82019492732107

H -2.36739801035536 -1.09666286205127 8.29980854913906

C -2.58569785377390 2.31341943515702 8.48405002241615

C -2.43743732674736 -0.10977133102811 8.77583575196807

C -3.48121506888732 -3.38147433708547 10.74510428711936

C -2.62807319503257 2.47081401553278 9.84717074869898

C -2.47690465101657 0.01319589187940 10.18894171864795

H -2.70462177536199 3.49841548864508 10.22859921409813

C -2.29790702613801 -2.53545144299276 10.47513672811417

C -2.58003974958657 1.35884246094333 10.76723741213303

C -2.42334064732895 -1.11703189670234 11.07040169027586

N -5.02096545930902 3.33188081156549 14.05489843485903

C -1.04166302936676 -3.21442985519052 10.86249639360413

C -3.99950266580301 3.14672534414871 13.51370775001736

N -0.02976878091393 -3.73370184263049 11.13970187139616

C -2.62949666380892 1.51279183016346 12.17023622853027

C -2.48346597811203 -0.95826348324721 12.47130003775855

C -2.73181466018282 2.92437509934006 12.78276446468695

H -2.36899005996686 -3.10048893297041 12.98571567978876

C -2.58918731873488 0.37935474049945 13.04585572754587

C -2.44596019686748 -2.07861587069139 13.37894378563281

C -1.55711839160903 3.28534331286140 13.60733531814972

C -2.64844395038672 0.51317708377714 14.45569600238383

C -2.50698265562535 -1.91799906835677 14.74195012752481

H -2.72848638584619 1.50325174384909 14.92113964122465

H -2.47763853182110 -2.79288673748891 15.40672484929148

C -2.61058682481408 -0.60108928445592 15.32105281224093

N -0.60961637954040 3.58701726481248 14.22517021021836

C -2.67249237604120 -0.43587001705583 16.71559518569367

C -2.72825178953942 -0.30077943294751 17.94989931074864

C -2.79186435639942 -0.13768431282645 19.33983390040990

H -2.91068168839169 2.04830951092409 19.25374210588998

H -2.68901255635609 -2.28269171888090 19.77338591699246

C -2.88841510185137 1.17365756259923 19.92063588111712

C -2.76289813799734 -1.27761662425811 20.21475346147833

C -2.95228424670212 1.33212193062071 21.29216775900366

C -2.82759830079897 -1.11399891295291 21.58561321630087

H -3.02631490532663 2.33392760100829 21.74102592199335

H -2.80630072295030 -1.98380219687549 22.25890745921973

C -2.92461560757652 0.19674871278891 22.18513644164073

S -3.00316541509056 0.39420902323449 23.86942672383374

H -2.74153135447061 3.66830526299206 11.96029275844599

H -2.24813390716398 -2.45492214993949 9.37008668737029

References

(S1) Fracasso, D.; Valkenier, H.; Hummelen, J. C.; Solomon, G. C.; Chiechi, R. C. Evidence for Quantum Interference in SAMs of Arylethynylene Thiolates in Tunneling Junctions With Eutectic Ga–In (EGaIn) Top-Contacts. *J. Am. Chem. Soc.* **2011**, *133*, 9556–9563.

(S2) Huo, L.; Zhou, Y.; Li, Y. Alkylthio-Substituted Polythiophene: Absorption and Photovoltaic Properties. *Macromol. Rapid Commun.* **2009**, *30*, 925–931.

(S3) Carlotti, M.; Degen, M.; Zhang, Y.; Chiechi, R. C. Pronounced Environmental Effects on Injection Currents in EGaIn Tunneling Junctions Comprising Self-Assembled Monolayers. *J. Phys. Chem. C* **2016**, *120*, 20437–20445.

(S4) Carlotti, M.; Kovalchuk, A.; Wächter, T.; Qiu, X.; Zharnikov, M.; Chiechi, R. C. Conformation-Driven Quantum Interference Effects Mediated by Through-Space Conjugation in Self-Assembled Monolayers. *Nat. Commun.* **2016**, *7*, 13904.

(S5) Shirley, D. A. High-Resolution X-Ray Photoemission Spectrum of the Valence Bands of Gold. *Phys. Rev. B* **1972**, *5*, 4709–4714.

(S6) Moulder, J. F.; Stickle, W. F.; Sodhi, P. E., R. N. S.bol; Bomben, K. D. In *Handbook of X-Ray Photoelectron Spectroscopy: A Reference Book of Standard Spectra for Identification & Interpretation of XPS Data*; Chastain, J., Ed.; Perkin-Elmer, Physical Electronics Division, 1993.

(S7) Tseng, T.-C.; Urban, C.; Wang, Y.; Otero, R.; Tait, S. L.; Alcamí, M.; Écija, D.; Trelka, M.; Gallego, J. M.; Lin, N.; Konuma, M.; Starke, U.; Nefedov, A.; Langner, A.; Wöll, C.; Herranz, M. A.; Mart in, F.; Martín, N.; Kern, K.; Miranda, R. Charge-Transfer-Induced Structural Rearrangements at Both Sides of Organic/Metal Interfaces. *Nat. Chem.* **2010**, *2*, 374–.

(S8) Kumar, S.; van Herpt, J. T.; Gengler, R. Y. N.; Feringa, B. L.; Rudolf, P.; Chiechi, R. C. Mixed Monolayers of Spiropyrans Maximize Tunneling Conductance Switching by Photoisomerization at the Molecule–Electrode Interface in EGaIn Junctions. *J. Am. Chem. Soc.* **2016**, *138*, 12519–12526.

(S9) Ishida, T.; Choi, N.; Mizutani, W.; Tokumoto, H.; Kojima, I.; Azehara, H.; Hokari, H.; Akiba, U.; Fujihira, M. High-Resolution X-Ray Photoelectron Spectra of Organosulfur Monolayers on Au(111): S(2p) Spectral Dependence on Molecular Species. *Langmuir* **1999**, *15*, 6799–6806.

(S10) Ishida, T.; Hara, M.; Kojima, I.; Tsuneda, S.; Nishida, N.; Sasabe, H.; Knoll, W. High Resolution X-Ray Photoelectron Spectroscopy Measurements of Octadecanethiol Self-Assembled Monolayers on Au(111). *Langmuir* **1998**, *14*, 2092–2096.

(S11) Stohr, J. *NEXAFS Spectroscopy*; Springer-Verlag Berlin Heidelberg, 1992.

(S12) Batson, P. E. Carbon 1s Near-Edge-Absorption Fine Structure in Graphite. *Phys. Rev. B* **1993**, *48*, 2608–2610.

(S13) Zharnikov, M. High-Resolution X-Ray Photoelectron Spectroscopy in Studies of Self-Assembled Organic Monolayers. *J. Electron Spectrosc. Relat. Phenom.* **2010**, *178–179*, 380–393.

(S14) Ratner, B. D.; Castner, D. G. *Surface Analysis - The Principal Techniques*; John Wiley & Sons, Ltd, 1997; pp 47–112.

(S15) Ballav, N.; Schüpbach, B.; Neppl, S.; Feulner, P.; Terfort, A.; Zharnikov, M. Biphenylnitrile-Based Self-Assembled Monolayers on Au(111): Spectroscopic Characterization and Resonant Excitation of the Nitrile Tail Group. *The Journal of Physical Chemistry C* **2010**, *114*, 12719–12727.

(S16) Hamoudi, H.; Kao, P.; Nefedov, A.; Allara, D. L.; Zharnikov, M. X-Ray Spectroscopy Characterization of Self-Assembled Monolayers of Nitrile-Substituted Oligo(phenylene Ethynylene)s With Variable Chain Length. *Beilstein J. Nanotechnol.* **2012**, *3*, 12–24.

(S17) Thome, J.; Himmelhaus, M.; Zharnikov, M.; Grunze, M. Increased Lateral Density in Alkanethiolate Films on Gold by Mercury Adsorption. *Langmuir* **1998**, *14*, 7435–7449.

(S18) Chesneau, F.; Schüpbach, B.; Szelagowska-Kunstman, K.; Ballav, N.; Cyganik, P.; Terfort, A.; Zharnikov, M. Self-assembled monolayers of perfluoroterphenyl-substituted alkanethiols: specific characteristics and odd-even effects. *Physical chemistry chemical physics : PCCP* **2010**, *12*, 12123–12137.

(S19) Lamont, C.; Langmuir, J. W.; 1999, Attenuation Length of Electrons in Self-Assembled Monolayers of n-Alkanethiols on Gold. *ACS Publications*

(S20) Matter, F. S. J. o. P. C.; 2004, Self-assembled monolayers: from 'simple'model systems to biofunctionalized interfaces. *iopscience.iop.org*

(S21) Simeone, F. C.; Yoon, H. J.; Thuo, M. M.; Barber, J. R.; Smith, B.; Whitesides, G. M. Defining the Value of Injection Current and Effective Electrical Contact Area for EGaIn-based Molecular Tunneling Junctions. *J. Am. Chem. Soc.* **2013**, *135*, 18131–18144.

(S22) Wimbush, K. S.; Fratila, R. M.; Wang, D.; Qi, D.; Liang, C.; Yuan, L.; Yakovlev, N.; Loh, K. P.; Reinhoudt, D. N.; Velders, A. H., et al. Bias Induced Transition From an Ohmic to a Non-Ohmic Interface in Supramolecular Tunneling Junctions With Ga 2 O 3/EGaIn Top Electrodes. *Nanoscale* **2014**, *6*, 11246–11258.

(S23) Pourhossein, P.; Vijayaraghavan, R. K.; Meskers, S. C. J.; Chiechi, R. C. Optical Modulation of Nano-Gap Tunnelling Junctions Comprising Self-Assembled Monolayers of Hemicyanine Dyes. *Nat. Commun.* **2016**, *7*, 11749–.

(S24) Weiss, E. A.; Chiechi, R. C.; Kaufman, G. K.; Kriebel, J. K.; Li, Z.; Duati, M.; Rampi, M. A.; Whitesides, G. M. Influence of Defects on the Electrical Characteristics of Mercury-Drop Junctions: Self-Assembled Monolayers of N-Alkanethiolates on Rough and Smooth Silver. *J. Am. Chem. Soc.* **2007**, *129*, 4336–4349.

(S25) Kaliginedi, V.; Moreno-García, P.; Valkenier, H.; Hong, W.; García-Suárez, V. M.; Buiter, P.; Otten, J. L. H.; Hummelen, J. C.; Lambert, C. J.; Wandlowski, T. Correlations Between Molecular Structure and Single-Junction Conductance: A Case Study With Oligo(phenylene-Ethynylene)-Type Wires. *J. Am. Chem. Soc.* **2012**, *134*, 5262–5275.

(S26) Zhao, A.; Tan, S.; Li, B.; Wang, B.; Yang, J.; Hou, J. G. STM tip-assisted single molecule chemistry. *Phys. Chem. Chem. Phys.* **2013**, *15*, 12428–12441.

(S27) Valkenier, H.; Huisman, E. H.; van Hal, P. A.; de Leeuw, D. M.; Chiechi, R. C.; Hummelen, J. C. Formation of High-Quality Self-Assembled Monolayers of Conjugated Dithiols on Gold: Base Matters. *J. Am. Chem. Soc.* **2011**, *133*, 4930–4939.

(S28) Neese, F. The ORCA Program System. *Wiley Interdiscip. Rev.: Comput. Mol. Sci.* **2012**, *2*, 73–78.

(S29) Neese, F. Software update: the ORCA program system, version 4.0. *Wiley Interdisciplinary Reviews: Computational Molecular Science*

(S30) Herrmann, C.; Gross, L.; Steenbock, T.; Deffner, M.; Voigt, B. A.; Solomon, G. C. ARTAIOS - A Transport Code for Postprocessing Quantum Chemical Electronic Structure Calculations, Available From https://www.chemie.uni-hamburg.de/ac/herrmann/software/index.html. 2016.

(S31) Weigend, F.; Ahlrichs, R. Balanced Basis Sets of Split Valence{,} Triple Zeta Valence and Quadruple Zeta Valence Quality for H to Rn: Design and Assessment of Accuracy. *Phys. Chem. Chem. Phys.* **2005**, *7*, 3297–3305.

(S32) Herrmann, C.; Solomon, G. C.; Subotnik, J. E.; Mujica, V.; Ratner, M. A. Ghost Transmission: How Large Basis Sets Can Make Electron Transport Calculations Worse. *J. Chem. Phys.* **2010**, *132*, 024103.

(S33) Zhang, Y.; Ye, G.; Soni, S.; Qiu, X.; Krijger, T. L.; Jonkman, H. T.; Carlotti, M.; Sauter, E.; Zharnikov, M.; Chiechi, R. C. Controlling destructive quantum interference in tunneling junctions comprising self-assembled monolayers via bond topology and functional groups. *Chem. Sci.* **2018**, *9*, 4414–4423.

(S34) Abu-Husein, T.; Schuster, S.; Egger, D. A.; Kind, M.; Santowski, T.; Wiesner, A.; Chiechi, R.; Zojer, E.; Terfort, A.; Zharnikov, M. The Effects of Embedded Dipoles in Aromatic Self-Assembled Monolayers. *Advanced Functional Materials 25*, 3943–3957.

(S35) Kovalchuk, A.; Abu-Husein, T.; Fracasso, D.; Egger, D. A.; Zojer, E.; Zharnikov, M.; Terfort, A.; Chiechi, R. C. Transition voltages respond to synthetic reorientation of embedded dipoles in self-assembled monolayers. *Chem. Sci.* **2016**, *7*, 781–787.

(S36) Cabarcos, O. M.; Schuster, S.; Hehn, I.; Zhang, P. P.; Maitani, M. M.; Sullivan, N.; Guigère, J.-B.; Morin, J.-F.; Weiss, P. S.; Zojer, E.; Zharnikov, M.; Allara, D. L. Effects of Embedded Dipole Layers on Electrostatic Properties of Alkanethiolate Self-Assembled Monolayers. *The Journal of Physical Chemistry C* **2017**, *121*, 15815–15830.
